# Bacterial MgrB peptide activates chemoreceptor Fpr3 in mouse accessory olfactory system and drives avoidance behaviour

**DOI:** 10.1038/s41467-019-12842-x

**Published:** 2019-10-25

**Authors:** Bernd Bufe, Yannick Teuchert, Andreas Schmid, Martina Pyrski, Anabel Pérez-Gómez, Janina Eisenbeis, Thomas Timm, Tomohiro Ishii, Günter Lochnit, Markus Bischoff, Peter Mombaerts, Trese Leinders-Zufall, Frank Zufall

**Affiliations:** 1 Center for Integrative Physiology and Molecular Medicine, Saarland University, 66424 Homburg, Germany; 2 Institute for Medical Microbiology and Hygiene, Saarland University, 66424 Homburg, Germany; 3 Protein Analytics, Institute of Biochemistry, Faculty of Medicine, Justus-Liebig-University Giessen, Friedrichstrasse 24, 35392 Giessen, Germany; 4 Max Planck Research Unit for Neurogenetics, Max-von-Laue-Strasse 4, 60438 Frankfurt, Germany; 5 Present Address: Molecular Immunology Section, Faculty of Computer Science and Microsystems Engineering, University of Applied Sciences Kaiserslautern, Amerikastrasse 1, 66482 Zweibrücken, Germany; 6 Present Address: Department of Molecular Medicine, The Scripps Research Institute, 10550 N Torrey Pines Road, La Jolla, CA 92037 USA; 7 Present Address: Department of Cell Biology, Graduate School of Medical and Dental Science, Tokyo Medical and Dental University, 1-5-45 Yushima, Bunkyo-ku, Tokyo, 113-8510 Japan

**Keywords:** Neuroscience, Olfactory system

## Abstract

Innate immune chemoreceptors of the formyl peptide receptor (Fpr) family are expressed by vomeronasal sensory neurons (VSNs) in the accessory olfactory system. Their biological function and coding mechanisms remain unknown. We show that mouse Fpr3 (Fpr-rs1) recognizes the core peptide motif f-MKKFRW that is predominantly present in the signal sequence of the bacterial protein MgrB, a highly conserved regulator of virulence and antibiotic resistance in *Enterobacteriaceae*. MgrB peptide can be produced and secreted by bacteria, and is selectively recognized by a subset of VSNs. Exposure to the peptide also stimulates VSNs in freely behaving mice and drives innate avoidance. Our data shows that Fpr3 is required for neuronal detection and avoidance of peptides derived from a conserved master virulence regulator of enteric bacteria.

## Abstract (web summary)

The role of chemoreceptors on vomeronasal neurons are not fully understood. Here the authors show that in mice, the vomeronasal chemoreceptor Fpr3 responds to peptides from the bacterial MgrB protein, and that exposure to these peptides drives an avoidance response.

## Introduction

A diversity of defence mechanisms have evolved to reduce the burden of infectious disease and to enable survival and reproduction in the face of tremendous pathogen challenges. These mechanisms are generally categorised into three basic strategies: avoidance of exposure to the pathogen (A), resistance to infection (R), and tolerance to the presence of the pathogen (T)^1,2^. Avoidance is likely to be the most cost-effective way of defence^1^, yet investigations into the role of the nervous system in mediating this function have only recently begun, and the cellular and molecular mechanisms for infection-avoidance behaviour remain largely unknown. Avoidance mechanisms are based on the remote sensing of pathogen-associated metabolites^1^ that activate the olfactory and/or other chemosensory systems in the body. In fact, there is now increasing evidence from nematodes^3^ and fruitflies^4^ to fishes^5^, rodents^6,7^ and humans^8,9^ that chemosensory cues associated with harmful microbes or inflammation and reduced fitness can be detected and avoided by conspecifics. In the mouse, interest has focused on the vomeronasal organ (VNO), a chemosensory organ that provides sensory input to the accessory olfactory bulb (AOB) in the olfactory forebrain^10–12^. Vomeronasal sensory neurons (VSNs) detect chemostimuli that result in instinctive decisions causing an individual either to be attracted to another individual, to avoid it, or even to attack and kill it^11–15^. VSNs are also implicated in social identification processes involved in the neural recognition of health status, immunological fitness and genetic compatibility^11,16^. The vomeronasal system mediates the detection and innate avoidance of sick conspecifics^17^.

Formyl peptide receptors (Fprs) are innate immune chemoreceptors of the seven transmembrane domain superfamily that recognise bacterial and mitochondrial formylated peptides as well as some other ligands^18–21^. In the immune system, Fprs play important roles in the initial sensing of infection through the detection of pathogen- and danger-associated molecular patterns that signal the presence of bacteria^18,19^. Subsets of mouse VSNs express several distinct immune-related molecules^22–24^ including five Fprs^25–29^. In analogy to their role in innate immunity, a chemosensory function associated with the identification of pathogens^26^ or an assessment of the bacterial flora of conspecifics^25^ has been hypothesised for vomeronasal Fprs as well. However, their precise function in olfaction has remained elusive; no Fpr knockout mice have been analysed in this regard; and there is no causal relationship between Fpr activation and odour-guided behaviour.

Here, we explore the role of Fpr3 (also known as Fpr-rs1, Fprl1, Lxa4r, or LXA4-R; see MGI gene ID 1194495) in olfaction. We find that Fpr3 functions as a pattern recognition receptor for a distinct subset of *N*-formyl methionine-containing (fMet) peptides that are predominantly present in the signal sequence of the bacterial protein MgrB, a highly conserved regulator of virulence and antibiotic resistance in *Enterobacteriaceae*. Native mouse VSNs detect such peptides with exquisite selectivity, and peptide-evoked cellular responses are abolished in a novel gene-targeted mouse strain carrying a knockout mutation in the *Fpr3* locus. MgrB peptide stimulates VSNs of freely behaving mice and drives a form of innate avoidance that requires Fpr3, the G protein Gαo, and the ion channel Trpc2. We conclude that the chemoreceptor Fpr3 is required in the accessory olfactory system for sensing specific MgrB and MgrB-like peptides and for enabling behavioural avoidance to these bacterial cues.

## Results

### Fpr3 detects peptides of bacterial virulence regulators

**Fig. 1:**
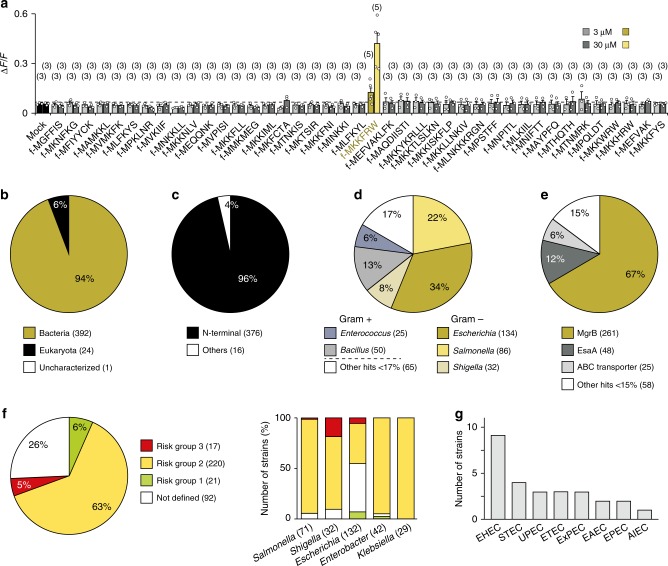
Fpr3 detects signal sequences from bacterial virulence-associated proteins. **a** Heterologous expression and high-throughput agonist screening reveals exquisite selectivity of Fpr3. Bars denote mean Ca^2+^ responses of Fpr3-transfected HEK293T cells to one of 41 different bacterial signal peptide sequences, at 3 or 30 µM. f-MKKFRW (mellow yellow) caused Fpr3 activation, but all other tested peptides (grey bars) did not. Numbers above each bar indicate the number of independent experiments, each one carried out in triplicates. The dashed line denotes the negative control signal obtained from application of assay buffer without an added peptide (black bars, mock). Amino acid sequences of the 6–9 residue-long peptides are displayed in one-letter code; (f-) indicates a formylation of the *N*-terminal methionine. Error bars indicate standard deviations (s.d.). **b**–**e** Bioinformatic analyses of MKKFRW-containing proteins as annotated in UniProt. Numbers in parentheses indicate the number of database entries. **b** Distribution of MKKFRW-containing proteins in different organisms. **c** Location of the MKKFRW motif within a given protein from all 392 UniProt entries found in bacteria. **d** Distribution of the MKKFRW motif between Gram-positive and Gram-negative bacteria. **e** Distribution of proteins that contain the MKKFRW motif. **f**, **g** Pathogenicity potential of *mgrB*+ bacterial strains. **f** Biosafety level classification of *mgrB*+ bacterial strains. Risk group 3, red; risk group 2, yellow; risk group 1, green; non-annotated strains, white. Numbers in parentheses indicate number of strains found in each genus (right panel). **g** The *mgrB*+ list comprises various obligate pathogenic *E. coli* variants such as enterohemorrhagic *E. coli* (EHEC), Shiga toxin producing *E. coli* (STEC), uropathogenic *E. coli* (UPEC), enterotoxigenic *E. coli* (ETEC), extraintestinal pathogenic *E. coli* (ExPEC), enteroaggregative *E. coli* (EAEC), enterophathogenic *E. coli* (EPEC), or adherent invasive *E. coli* (AIEC) that are listed as risk group 2 and 3 (EHEC) organisms, respectively. Source data are provided as a Source Data file

To define the agonist spectrum of mouse Fpr3, we performed high-throughput Ca^2+^ imaging using human HEK293T cells that were transiently transfected with an Fpr3 expression vector^21,30^ (Fig. 1). We challenged cells with individual members from a panel of 41 fMet peptides of 6–9 amino acids (at 3 or 30 µM), each contained within the *N*-terminus of bacterial signal peptides (Fig. 1a and Supplementary Data 1). This selection of peptides is representative for the *N*-termini of ~14,700 bacterial proteins (UniProt), and shows considerable variability in amino acid sequence. Strikingly, only a single peptide (f-MKKFRW, 3 µM) activated Fpr3-expressing HEK293T in these experiments (Fig. 1a). The other 40 peptides from the panel did not elicit a specific Ca^2+^ response, even when used at 10-fold higher concentrations. Thus, Fpr3 appears to be exquisitely selective, in that it is tuned to a peptide motif present in a subset of bacterial signal peptides (see also Methods).

Bioinformatic analyses of MKKFRW-containing proteins revealed four main observations. First, the MKKFRW motif is highly enriched in bacteria: 392 of all 417 database entries (94%, UniProt) comprising this sequence are from bacteria (Fig. 1b and Supplementary Table 2). Second, 96% of these 392 bacterial proteins carry the MKKFRW sequence at their *N*-terminus (Fig. 1c and Supplementary Data 2). Third, this sequence is predominantly present in membrane proteins of *Enterobacteriaceae*, a large family of Gram-negative bacteria including *Escherichia*, *Salmonella* and *Shigella* species (252/392, 64%), but is also found in some Gram-positive genera such as *Bacillus* and *Enterococcus* species (75/392, 19%) (Fig. 1d and Supplemementary Data 2). Fourth and most remarkably, 67% (261/392) of all hits in bacteria can be attributed to one bacterial gene, *mgrB* (Fig. 1e), which encodes a small virulence-associated protein that functions as a negative regulator of the two-component PhoP/PhoQ signalling system^31–33^. This motif is also present at the *N*-termini of some virulence-associated proteins from Gram-positive bacteria, i.e. the secretion accessory factor EsaA (48/392, 12%) and ABC transporter proteins (25/392, 6%) (Fig. 1e). Hence, 85% (334/392) of all database entries from bacterial MKKFRW-containing proteins represent virulence systems that promote the survival of bacterial cells within mammalian hosts^34–37^.

In silico analyses suggest a strong correlation between the pathogenicity of these bacteria and the presence of *mgrB* in their genomes. Screening of the UniProt database identified 350 bacterial genomes that encode annotated full-length MgrB amino-acid sequences (Supplementary Data 3), and we then assessed the pathogenicity of these *mgrB*-positive (+) strains (Fig. 1f, g). Most of these strains belong to only five genera of the family *Enterobacteriaceae*: *Enterobacter*, *Escherichia*, *Klebsiella*, *Salmonella* and *Shigella*, all known to comprise bacterial strains that may cause severe infectious diseases in mammals (Fig. 1f). Several of the *mgrB*+ strains are pan-species pathogens capable of infecting humans as well as cattle, mice and birds (Supplementary Data 3). When assigning the *mgrB*+ bacterial strains to risk groups using American and European biological safety databases (see Online Methods), we found 63% in risk group 2 (potentially pathogenic or infectious), 5% in risk group 3 (associated with serious or lethal human disease), 6% in risk group 1 (non-pathogenic), whereas the remaining 26% were not annotated (Fig. 1f). Furthermore, several of the *Escherichia coli* strains listed in the UniProt database belong to well-characterised classes or pathotypes of *E. coli* capable of causing disease in humans (Fig. 1g and Supplementary Data 4). These data link the presence of MgrB in bacteria with a high risk of pathogenicity. We note that the *mgrB* gene has also been recognised as a key target for acquired antibiotic resistance^38–41^.

### Fpr3 is a pattern recognition receptor for MgrB peptides

**Fig. 2:**
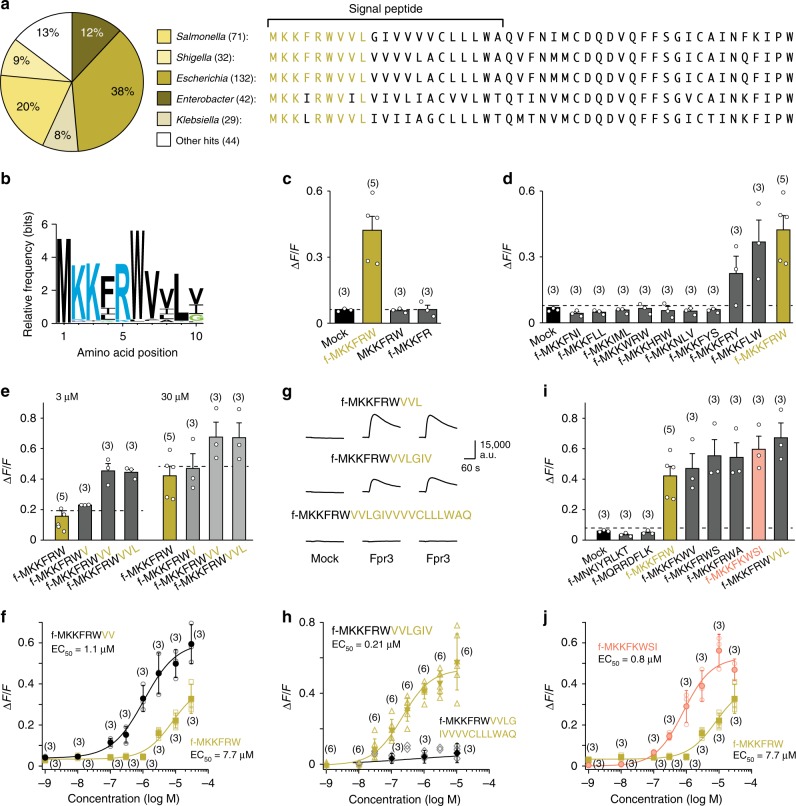
Fpr3 detects fMet peptides from the conserved MgrB signal sequence. **a** Distribution of *mgrB* in *Enterobacteriaceae* and amino acid (aa) sequences (one-letter code) of typical MgrB proteins. **b** Sequence logo displays the degree of aa conservation through letter size in the first 10 *N*-terminal residues of MgrB obtained from all 350 UniProt entries. **c**–**j** Mean Ca^2+^ responses of Fpr3-transfected HEK293T cells after exposure to fMet peptides structurally related to f-MKKFRW. **c** Fpr3 recognises the core agonist f-MKKFRW but not two truncated variants (30 µM each) that lack either the *N*-terminal formyl group (f-) or the *C*-terminal tryptophan (W). **d** Selectivity of Fpr3 to a panel of nine bacterial signal peptide fragments (30 µM each) with high sequence similarity to f-MKKFRW. **e** Effect of sequence elongation measured at 3 or 30 µM, respectively. Note that Ca^2+^ responses increased with longer peptides. **f** Concentration-response curves to stimulation with the core motif (gold metallic) or an elongated octapeptide (black). The EC_50_ value shifted to the left with the elongated sequence. **g** Fpr3 recognises *N*-terminal MgrB variants with 9 and 12 aa residues, respectively, but not a 21-residue variant. Shown are repeated Ca^2+^ responses of HEK293T cells transfected with an empty vector (mock) or Fpr3 after stimulation with MgrB peptides of 9, 12 and 21 residues (10 µM each). **h** Concentration-response curves to stimulation with a 12-mer (gold metallic) or 21-mer peptide (black). No signals were obtained for the 21-mer peptide. **i** Selectivity of Fpr3 to an additional panel of seven bacterial signal peptide fragments (30 µM each). Five of these display high sequence similarity to f-MKKFRW. Note that f-MKKFKWSI (salmon) found in some EsaA signal peptides produced similar response amplitudes as the elongated MgrB sequence. **j** Dose-response curve and EC_50_ value for stimulation with f-MKKFKWSI (salmon). **c**–**f**, **h**–**j** Numbers in parentheses indicate the number of independent experiments. Dashed line denotes the negative control signal obtained from mock application of assay buffer. Error bars, s.d. Source data are provided as a Source Data file

Pattern recognition receptors^42^ detect evolutionary conserved structures that are difficult to alter because they are essential for the microorganisms. Indeed, the MKKFRW sequence shows a high degree of conservation at the *N*-terminus of MgrB proteins of *Enterobacteriaceae*, also evident in sequence logo comparisons (Fig. 2a, b). The sequence logo of all 350 MgrB *N*-termini displays a much higher degree of conservation than that of two other bacterial proteins, EsaA and PotC, which also comprise an MKKFRW motif in a number of species (Supplementary Fig. 1). The first six residues containing the MKKRFW core motif show the highest degree of conservation. A certain degree of conservation is also evident for the next three residues (VVL) following the MKKRFW motif (Fig. 2a, b).

We thus hypothesised that f-MKKFRW serves as a minimal consensus sequence for recognition by Fpr3; that deviations from this consensus sequence diminish recognition capabilities by the receptor; and that extension to f-MKKFRWVVL may improve recognition. Indeed, removal of either the formyl group or the last amino acid completely abolished Fpr3 activation (Fig. 2c). Experiments employing nine sequence-related derivatives of f-MKKFRW confirmed that relatively small deviations from the consensus sequence diminish or abolish Fpr3 activation (Fig. 2d). By contrast, elongation of the core motif with V, VV, VVL or VVLGIV enhanced agonist efficacy and/or sensitivity (Fig. 2e-h). However, a 21-mer peptide comprising the entire MgrB signal sequence failed to activate Fpr3 (Fig. 2g, h). Together, these results identify fMet peptides derived from the MgrB signal sequence as a key agonist motif for Fpr3. These findings argue that the signal peptide has to be cleaved and secreted before appropriate fragments can be recognised by Fpr3, consistent with previous conclusions^21,43^.

Further analyses revealed that Fpr3 also responds to a limited number of additional, structurally related peptides not found in MgrB (Fig. 2i, j). Specifically, the EsaA protein of several *Bacillus* strains, associated with type VII protein secretion systems that are required for virulence and host-pathogen interactions^44,45^, contains a signal sequence (f-MKKFKWSI) that is an effective agonist of Fpr3 (Fig. 2i, j). Thus, Fpr3 could have a broader role in pathogen detection and may additionally recognise other specific, virulence-associated sequences from Gram-positive bacteria.

### Bacteria produce and secrete MgrB-derived fMet peptides

**Fig. 3:**
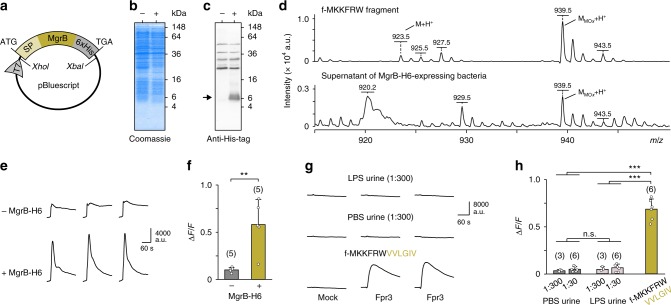
Fpr3 detects supernatant of MgrB-expressing bacteria but not urine from LPS-injected mice. **a** Scheme of the bacterial expression vector p*mgrB-H6* encoding a fusion protein consisting of MgrB together with its *N*-terminal signal peptide (SP) and a *C*-terminal His-tag (6 x His, H6) under control of a T7-RNA polymerase promoter (T7). Restriction sites for cloning were *Xba*I and *Xho*I, start and stop codon are indicated by ATG and TGA, respectively. **b** Commassie-stained SDS–PAGE from bacteria overexpressing MgrB-H6 (+) or a non-transfected control strain (−). **c** Western blot using an anti-His-tag antibody to visualise MgrB-H6 at the expected size of 6.5 kDa (black arrow). **d** MALDI-TOF mass spectrometry of the synthetic MgrB fragment f-MKKFRW (upper panel) and a sterile filtrated bacterial supernatant (lower panel) from MgrB-H6-expressing bacteria. The synthetic peptide exists in two forms: M + H^+^, which is non-oxidised and has a molecular mass of 923.5 Da, and M_MOx_ + H^+^, which is oxidised at the methionine and has a molecular mass of 939.5 Da. The M_MOx_ + H^+^ form was also detected in sterile filtrated supernatants from MgrB-H6-expressing bacteria. **e** Representative intracellular Ca^2+^ signals of Fpr3-transfected HEK293T cells to stimulation with bacterial supernatants (1:6 diluted) produced by a non-transfected control strain (- MgrB-H6) or MgrB-H6-expressing bacteria (+ MgrB-H6). Unpaired t-test: *t*(8) = 3.99, **P < 0.01. **f** Mean Ca^2+^ peak responses of Fpr3-transfected HEK293T cells after exposure to supernatant (1:6 diluted) from bacteria overexpressing MgrB-H6 (+) or a non-transfected control strain (−). Error bars, s.d. **g** Representative Ca^2+^ transients of Fpr3-transfected HEK293T cells to stimulation with urine from LPS- (upper panel) or PBS-injected (middle panel) mice at a dilution of 1:300. Stimulation with 10 µM of the 12-mer MgrB derivative (lower panel) served as positive control. **h** Mean Ca^2+^ responses of Fpr3-transfected HEK293T cells to a 1:30 dilution of the urine samples or the 12-mer MgrB derivative (10 µM). ANOVA: *F*(4,24) = 122.88 *P* < 0.0001; Tukey: ***P < 0.001, ns, *P* = 0.95–0.99. Error bars, s.d. Source data are provided as a Source Data file

Low molecular weight fMet peptides present in signal sequences of specific bacterial proteins are produced and secreted by intestinal bacteria in vitro and in vivo, and are found in intestinal luminal contents such as faecal dialysates at micromolar concentrations^46–49^. Whether natural MgrB-derived fMet peptides functioning as Fpr3 agonists are produced and secreted by bacteria is unknown. We expressed MgrB in bacteria and asked whether bacterial supernatants contain *N*-terminally formylated MgrB fragments that activate Fpr3. We subcloned a copy of the *mgrB* gene with an additional *C*-terminal His-tag (H6) into a bacterial expression vector (Fig. 3a and Supplementary Fig. 1d). Next, we transfected this construct into *E. coli* BL21 bacteria (Fig. 3b), which already contain an endogenous copy of *mgrB*. Western blot staining using an anti-His-tag antibody revealed MgrB-H6 expression by these bacteria (Fig. 3c). MALDI-TOF mass spectrometry of synthetic f-MKKFRW (Fig. 3d, upper panel) showed that this MgrB fragment exists in two forms: M_MOx_ + H^+^, which is oxidised at the methionine, and M + H^+^, which is not oxidised. Analysis of sterile filtrated supernatants produced by MgrB-H6-transfected bacteria showed that the oxidised M_MOx_ + H^+^ form of f-MKKFRW was also detectable in the supernatant of MgrB-H6-expressing bacteria (Fig. 3d, lower panel). Finally, we tested whether supernatants from MgrB-H6-expressing bacteria could activate Fpr3-expressing cells (Fig. 3e, f). Indeed, supernatants obtained from *E. coli* expressing His-tagged MgrB protein produced robust Ca^2+^ responses in Fpr3-expressing HEK293T cells, whereas supernatants from identically treated bacteria without plasmid-encoded MgrB showed only relatively small responses, which are likely to result from endogenous MgrB expression. Together, these results reveal that MgrB-derived fMet peptide can be produced and secreted by bacteria at sufficient concentrations to stimulate Fpr3.

### Fpr3 is not activated by urine from LPS-injected mice

Mice avoid conspecifics that are in an acute inflammatory state induced by injection of lipopolysaccharide (LPS)^17^, an endotoxin produced by Gram-negative bacteria that activates the immune system and mimics bacterial infection^17,50^. The VNO mediates this conspecific avoidance by detecting urinary cues from LPS-injected mice (LPS urine)^17^. Mouse urine contains numerous immune-related peptides that can stimulate VSNs^51–53^. Given that mutations in *mgrB* result in the upregulation of LPS modification under the control of PhoQ/PhoP signalling^38^, we asked whether LPS urine is detected by Fpr3 (Fig. 3g, h). We tested urine obtained from PBS-injected control mice versus that from LPS-injected mice in the in vitro Fpr3 expression assay. Fpr3-transfected HEK293T cells were exposed to two urine dilutions, but did not show Fpr3-dependent activation, either for PBS- or for LPS-injected mice, whereas a 12-mer MgrB derivative elicited robust responses (Fig. 3g, h). Thus, Fpr3 does not detect urine obtained from healthy mice or from mice with an LPS-induced inflammation. Together with subsequent behavioural experiments (see below), these results argue that chemoreceptors other than Fpr3 mediate the avoidance of LPS-treated mice or urine derived from these animals.

### MgrB peptides are detected by native VSNs

**Fig. 4:**
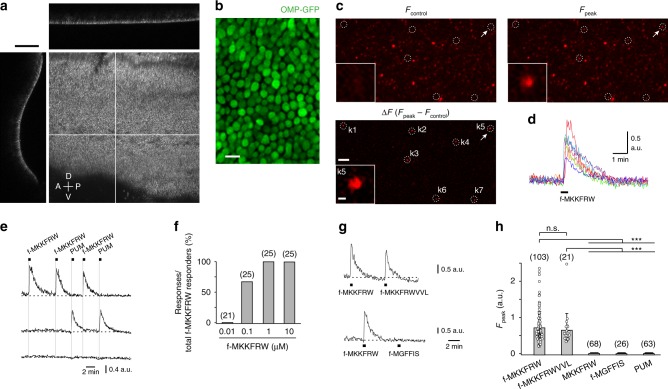
MgrB peptides are detected by native mouse VSNs. **a** Confocal image of an en face view of the VNE surface of a Trpc2-IRES-taumCherry mouse showing intrinsic mCherry fluorescence of VSN dendritic knobs. Orthogonal confocal section (top: xz or left: yz) demonstrates the crescent shape of the VNE surface. White lines indicate the position of the two orthogonal sections. Scale bar, 100 µm. A, anterior; P, posterior; D, dorsal; V, ventral. **b** High-resolution en face view on the VNE of an OMP-GFP mouse reveals tight packaging of VSN knobs expressing intrinsic GFP fluorescence from the *Omp* locus. **c** En face VNE confocal Ca^2+^ images of an adult B6 mouse showing Rhod-2 fluorescence at rest (Fcontrol), at the peak of the response (Fpeak) after stimulation with f-MKKFRW (1 µM), and as a ∆F image indicating responsive knobs. Inset, magnification of knob k5 (arrow). Scale bar, 20 µm; inset scale bar, 1 µm. **d** Time courses of f-MKKFRW-evoked Ca^2+^ transients from the seven knobs (k1-k7) shown in **c**. **e** (Top trace) Example of a Ca^2+^ response to f-MKKFRW that could be repeated multiple times. Note that PUM exposure failed to elicit a Ca^2+^ response in this knob. (Middle trace) Example of a knob that responded to PUM but not to f-MKKFRW. (Bottom trace) Example of a knob that responded to neither of the two stimuli. **f** The detection threshold for f-MKKFRW-evoked Ca^2+^ responses in VSNs of adult B6 mice was ≤0.1 µM. **g**, **h** Examples (**g**) and group data (**h**) showing the discriminative properties of f-MKKFRW-responsive VSN knobs to four different peptides and PUM stimulation. Data from individual knobs are plotted as open circles. Data in mean ± s.d. (*N*): f-MKKFRW, 0.71 ± 0.44 (103); f-MKKFRWVVL, 0.64 ± 0.45 (21); MKKFRW, 0.00 ± 0.00 (68); f-MGFFIS, 0.00 ± 0.00 (26); PUM, 0.00 ± 0.00 (63). Kruskal–Wallis ANOVA: *χ*^2^ (5) = 264.49; *P* < 0.0001; Mann–Whitney ***P < 0.001, ns, *P* = 0.39. Source data are provided as a Source Data file

Fpr3 is expressed in a small subset of VSNs^25,26^ but not in the main olfactory epithelium^25,26,28^. We used a VNO whole-mount preparation^26,54,55^ that enables large-scale mapping of neuronal activation evoked by molecular cues of defined concentrations at the level of an intact sensory organ. En face confocal imaging of the surface of the vomeronasal sensory epithelium (VNE) reveals a substantial fraction of the entire set of dendritic endings (knobs) extending from the ~150,000 VSNs^52^ to the lumen of the VNO (Fig. 4a, b; see also Supplementary Fig. 2). We loaded the VNE of adult C57BL/6N mice (either sex, referred to as B6; *n* = 54) with the Ca^2+^ indicator dye Rhod-2/AM, and performed dynamic Ca^2+^ mapping at a spatial resolution sufficient to image simultaneously the Ca^2+^ signals generated by thousands of dendritic knobs (Fig. 4c, d). This assay enabled us to analyse VNE areas of ~20,000–60,000 µm^2^ per experiment, corresponding to ~2250–6750 knobs. The results described below are based on analysing >170,000 individual knobs.

Exposure of the VNE to f-MKKFRW (1 µM) produced robust, transient Ca^2+^ elevations in a small subset of VSNs that could be repeated multiple times in a given knob (Fig. 4c–e). We estimate the fraction of knobs responding to f-MKKFRW as ~0.15%, which is similar to the fraction of cells in the VNE that express Fpr3^30^. Strikingly, the sensitivity and sequence-selectivity profile of these responses was nearly identical to the results that we obtained for heterologously expressed Fpr3. The detection threshold of native VSNs for f-MKKFRW was ≤0.1 µM (Fig. 4f), virtually the same as for Fpr3-expressing HEK293T cells (see Fig. 2f). VSNs that recognised f-MKKFRW responded also to the MgrB nonapeptide f-MKKFRWVVL (Fig. 4g, h). By contrast, both the non-formylated MKKFRW peptide and the fMet peptide f-MGFFIS, which activates Fpr1 and Fpr2 but not Fpr3^21^, failed to evoke Ca^2+^ responses in VSNs (Fig. 4g, h). A mix of diluted predator urine (PUM) known to stimulate a subset of VSNs^14^ also failed to activate f-MKKFRW-responsive cells but did activate other VSNs, providing an important control for the viability of the preparations (Fig. 4e, h). Taken together, these results identify a subset of mouse VSNs that recognise a peptide motif linked to a molecularly-defined bacterial virulence regulator with exquisite selectivity. The receptive properties of these native VSNs closely match those of Fpr3-expressing HEK293T cells.

### MgrB peptide is detected by B2m-expressing VSNs

**Fig. 5:**
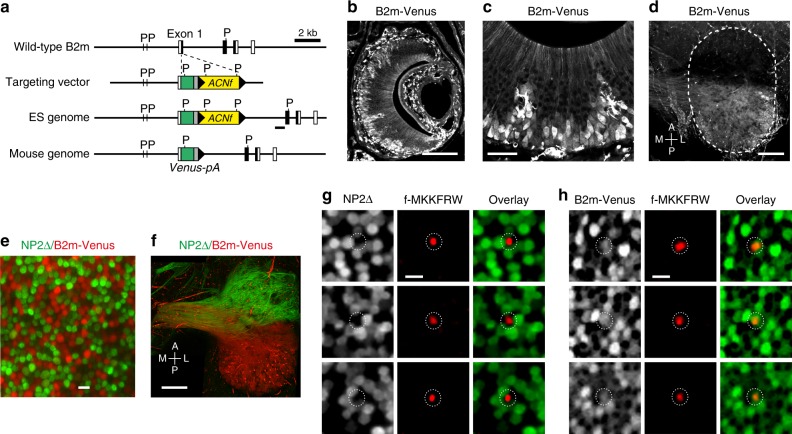
Recognition of MgrB peptide occurs in VSNs that express B2m. **a** Targeted mutagenesis of the *B2m* locus on Chromosome 2. Exon 1 of the *B2m* coding sequence is replaced with a *Venus-pA-ACNf* cassette by homologous recombination in ES cells. The *ACNf* cassette self-excises during transmission through the male germline, leaving a single *loxP* site (black triangle) behind in the mouse genome. *pA* represents the rabbit ß-globin poly-A signal region. The external probe used for ES colony screening by Southern blot hybridisation is shown as a thick black line under the ES genome. Filled and open boxes represent coding and noncoding exons, respectively. P, *Pst*I restriction enzyme site. **b**, **c** Coronal sections of the VNO of a 3-week-old B2m-Venus +/− mouse. Intrinsic Venus fluorescence is shown. **d** Dorsal view of whole-mounts of right AOB of an 8-week old B2m-Venus +/− mouse. The area of the AOB is outlined with a dashed line. A, anterior; P, posterior; M, medial L; lateral. **e** An en face view on the VNE of a 14-week old NP2∆/B2m-Venus mouse reveals tight packaging and mosaic organisation of VSN dendritic knobs expressing intrinsic fluorescence of GFP from the *Nrp2* locus (green) or Venus from the *B2m* locus (red). **f** Brain targets of axonal projections of NP2- or B2m-expressing VSNs in the anterior (green) or posterior (red) AOB (dorsal view), respectively. **g**, **h** Shown are examples of Ca^2+^ activation maps (F/F_o_, red) obtained at peak fluorescence to stimulation with f-MKKFRW (1 µM) and superimposed onto maps showing Venus+ or GFP+ knobs. Responses occurred in Venus+ but not in GFP+ knobs. Experiments are representative of recordings from four heterozygous NP2∆ mice (*n*=28 knobs) and three heterozygous mice B2m-Venus (*n*=36 knobs). Scale bars, 200 µm in (**b**, **d**, **f**), 50 µm in (**c**), 5 µm in (**e**, **g**, **h**)

Fpr3 is expressed in a subset of VSNs that are located in the basal layer of the VNE and coexpress the G protein Gαo^25,26^. VSNs of this layer are also characterised by expression of beta-2 microglobulin (B2m)^22^, the light chain of major histocompatibility complex class I molecules. To determine whether f-MKKFRW-responsive cells involve this basal subpopulation, we constructed a novel gene-targeted mouse strain in which part of the coding sequence of *B2m* is replaced by that of Venus, a yellow fluorescent protein (Fig. 5a). We observed strong intrinsic Venus fluorescence in the basal VNE layer in coronal sections of heterozygous B2m-Venus mice (Fig. 5b, c) and in the posterior AOB, the brain target for VSN axons projecting from the basal VNE (Fig. 5d). We next crossed B2m-Venus mice with NP2∆ mice^56^ in which tauGFP is knocked into the neuropilin-2 (NP2 or Npr2) locus and in which apical VSNs that coexpress the G protein Gαi2 can be visualised. In this cross, referred to as NP2∆/B2m-Venus mice (heterozygous for both mutations), an en face view of the VNE surface of our whole-mount preparation reveals a diffuse mosaic organisation of GFP + and Venus + VSNs, with no VSNs expressing both reporters (Fig. 5e, f and Supplementary Fig. 2). This genetic labelling thus enables us to identify a given cell as a VSN of either population (apical or basal) during en face imaging. We then performed Ca^2+^ imaging in heterozygous NP2∆ or heterozygous B2m-Venus mice. These experiments revealed that Ca^2+^ responses evoked by f-MKKFRW (1 µM) occurred in Venus + knobs but not in GFP + knobs (Fig. 5g, h). Thus, MgrB peptide is detected in B2m-expressing VSNs but not in NP2-expressing cells, consistent with the expression of Fpr3 in a subset of cells in the basal VNE layer.

### ΔFpr3-lacZ mice reveal monoallelic expression of Fpr3

**Fig. 6:**
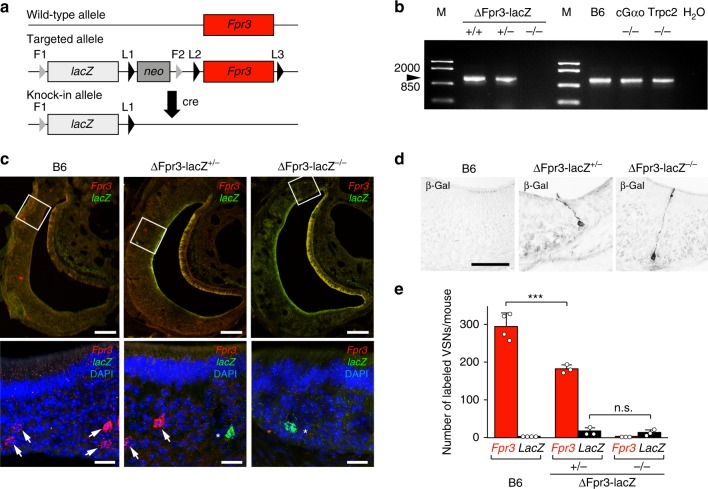
Generation and validation of ΔFpr3-lacZ mice. **a** The wild-type *Fpr3* allele (top) was mutated by replacement with an expression cassette carrying an *FRT* site (F1), the *lacZ* reporter sequence, a *loxP* site (L1), a neomycin resistance gene (*neo*), second *FRT* (F2) and *loxP* sites (L2), and a third *loxP* site (L3) downstream of the *Fpr3* coding exon. *Cre*-mediated recombination in mice carrying the targeted allele results in deletion of the coding region of *Fpr3* and replacement with *lacZ*. **b** RT-PCR analyses demonstrate that *Fpr3* mRNA of correct size (1023 nt) and sequence is present in the VNO of ΔFpr3-lacZ +/+ and +/− mice but absent in homozygous −/− mice (arrowhead). *Fpr3* mRNA is also detected in B6 mice and in mice that are −/− for Gαo or Trpc2. M, DNA size standard in base pairs; H_2_O, template water control. **c** RNAscope two-colour fluorescence in situ hybridisation for *Fpr3* (red) and *lacZ* (green) in 12-µm coronal sections of VNE. *Fpr3*-expressing VSNs are present in the basal VNE layer of B6 and ΔFpr3-lacZ +/− mice but absent in −/− mice. *LacZ*-labelled VSNs were detected only in +/− and −/− mice. Inset magnifications (bottom) illustrate that VSN somata express either *Fpr3* (arrows) or *lacZ* (asterisks) but not both. DAPI, nuclear counterstain. Scale bars, 100 µm (**c** top and **d**); 20 µm (**c** bottom). **d** Immunohistochemistry for the β-galactosidase (β-gal) reporter in VSNs of B6, ΔFpr3-lacZ +/− and −/− mice. **e** Quantification of *Fpr3*-positive (red columns) and *lacZ*-positive (black columns) VSNs (whole VNO, both sites) labelled by RNAscope in the three different genotypes. *n* ≥ 3 mice per genotype, ANOVA: *F*(5,19) = 189.1 *P* < 0.0001; LSD: ***P < 0.001, ns, *P* = 0.85. Source data are provided as a Source Data file

Is Fpr3 required for VSN responses to f-MKKFRW? We used a novel mouse strain carrying a lacZ-tagged *Fpr3* deletion allele, hereafter abbreviated as ΔFpr3-lacZ mice (Fig. 6a). ΔFpr3-lacZ mice are either wild-type (+/+), heterozygous (+/−) or homozygous (−/−) for the Fpr3 mutation. RT-PCR analyses demonstrated that *Fpr3* mRNA is present in the VNO of +/+and +/− mice but absent in −/− mice (Fig. 6b and Supplementary Fig. 3), whereas *Fpr-rs3*, *Fpr-rs4*, *Fpr-rs6* and *Fpr-rs7* could be readily detected in the VNO of −/− mice (Supplementary Fig. 3). *Fpr3* mRNA was also detectable in the VNO of B6 mice, of conditional null mutants of Gαo^57^, and of Trpc2 knockout mice^58^ (Fig. 6b).

We performed RNAscope two-colour fluorescence *in situ* hybridisation for *Fpr3* and *lacZ* (Fig. 6c) and immunohistochemistry for the β-galactosidase (β-gal) reporter (Fig. 6d) in coronal VNE sections of B6, ΔFpr3-lacZ + /− and −/− mice. *Fpr3*-expressing VSNs were readily detected in the basal VNE layer of B6 and +/− mice but were absent in −/− mice (Fig. 6c–e). *LacZ*- or β-gal-labelled VSNs were detected only in +/− and −/− mice. We observed no overlap between *Fpr3*- and *lacZ*-expressing VSNs in +/− mice, arguing for monoallelic expression of *Fpr3* (Fig. 6c). Furthermore, the number of VSNs that express the deletion allele was strongly diminished in both +/− and −/− mice (Fig. 6e). Selective, partial loss of VSNs has also been observed in mice deficient for other signalling molecules^15,57,59^.

### MgrB peptide detection requires Fpr3, Gαo and Trpc2

**Fig. 7:**
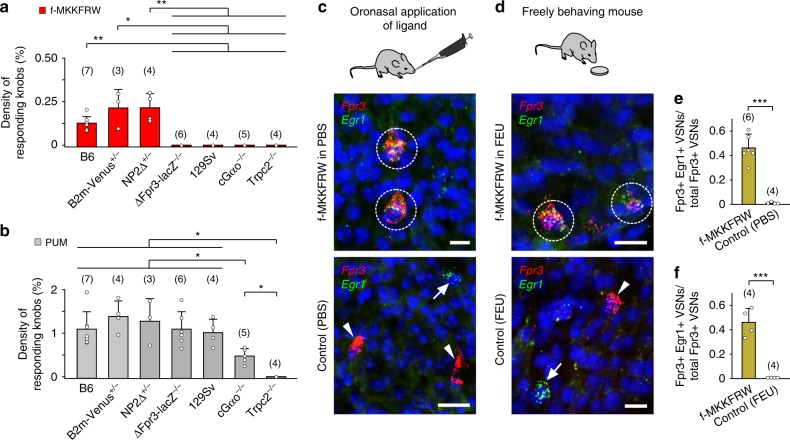
MgrB peptide detection requires Fpr3, Gαo, and Trpc2 and occurs in freely behaving mice. **a**, **b** B6, B2m-Venus +/−, and NP2∆ +/− mice show normal VSN Ca^2+^ responses to f-MKKFRW (1 µM) (**a**) or predator urine mix (PUM) (**b**). Mann–Whitney: *P* = 0.11–0.86. f-MKKFRW: B6: 0.12 ± 0.04%; B2m-Venus +/−: 0.2 ± 0.1%; NP2∆ +/−: 0.2 ± 0.09%. PUM: B6: 1.1 ± 0.4%; B2m-Venus +/−: 1.4 ± 0.4%; NP2∆ +/−: 1.3 ± 0.5%. ∆Fpr3-lacZ −/− and 129Sv mice fail to respond to f-MKKFRW (**a**) but show normal responses to PUM (**b**; ∆Fpr3-lacZ −/−: 1.09 ± 0.4%; 129Sv: 1.01 ± 0.3%). PUM-evoked responses are reduced in cGαo −/− mice and absent in Trpc2 −/− mice (**b**). Both types of mice fail to respond to f-MKKFRW (**a**). Data are expressed as density of responding knobs (in percentage) with results from individual experiments plotted as open circles. Number of mice tested per genotype and stimulus is shown in brackets above each bar with standard deviations (s.d.). Mann–Whitney: *P < 0.05, **P < 0.01. **c**–**f** f-MKKFRW activates *Fpr3*-expressing VSNs in vivo. **c**, **d** RNAscope fluorescence in situ hybridisation for *Fpr3* (red) and *Egr1* (green) after presentation of f-MKKFRW (100 µM). Both VSN stimulation by **c** oronasal peptide application or **d** voluntary peptide uptake in freely behaving mice reveal *Egr1* expression in about 50% of *Fpr3*-labelled VSNs (dotted circles). Control stimulations with (**c**) PBS or (**d**) FEU show no co-localisation, VSNs were labelled either for *Fpr3* (arrow heads) or for *Egr1* (arrows). Nuclear stain, DAPI (blue). Scale bars, 10 μm. **e**, **f** Column diagrams summarising co-localisation of *Fpr3* and *Egr1* in VSNs measured as fraction of the total number of *Fpr3*-positive VSNs. **e** Oronasal peptide stimulation (0.46 ± 0.11; 145/317 VSNs), PBS control (0 ± 0.01; 1/150 VSNs). Unpaired *t*-test: *t*(8) = 8.17, ***P < 0.001. **f** Voluntary peptide uptake (0.46 ± 0.12; 77/160 VSNs), FEU control (0 ± 0; 0/104 VSNs). Unpaired *t*-test: *t*(6)=7.67, ***P < 0.001. Numbers in brackets above each column indicate number of VNOs analysed. Data, mean ± s.d. Source data are provided as a Source Data file

To examine whether Fpr3 is required for VSN detection of MgrB peptide, we performed Rhod-2 confocal Ca^2+^ imaging and compared the responses evoked by f-MKKFRW (1 µM) in the VNE from mice of various genotypes (Fig. 7a, b). Ca^2+^ responses to PUM, which occurred in VSNs distinct from those responding to f-MKKFRW (Fig. 4e, h), were used as control in each experiment (Fig. 7b). Data were expressed as the density of responding knobs (in percentage, see Methods). In B6 mice, B2m-Venus+/− mice, and NP2∆+/− mice, we observed percentages ranging from 0.07 to 0.29% (0.16 ± 0.08, *n* = 14 mice) to f-MKKFRW (Fig. 7a) and from 0.71 to 1.93% (1.22 ± 0.4, *n* = 14 mice) to PUM exposure (Fig. 7b). By contrast, responses to f-MKKFRW were absent in ΔFpr3-lacZ −/− mice whereas responses to PUM were normal (Fig. 7a, b). 129Sv mice, which carry a nonfunctional *Fpr3* gene variant^30^, also showed no responses to f-MKKFRW, but normal responses to PUM. Moreover, responses to f-MKKFRW were absent in cGαo −/− mice and in Trpc2 −/− mice (Fig. 7a); responses to PUM were reduced to ~50% in cGαo −/− mice and were absent in Trpc2 −/− mice (Fig. 7b). Taken together, these results show that Fpr3, Gαo and Trpc2 are required for Ca^2+^ responses evoked by MgrB peptide in VSN dendritic knobs. We conclude that, in the VNE, f-MKKFRW is detected exclusively by Fpr3-expressing VSNs and that this detection requires Gαo and Trpc2.

### Detection of MgrB peptide in freely behaving mice

Nonvolatile chemostimuli such as peptides are known to gain access to the VNE of behaving mice during social investigation^16,55,60^. To assess specific activation of *Fpr3*-expressing VSNs in vivo, we stimulated adult male B6 mice with f-MKKFRW (100 µM) and then performed double-labelling RNAscope in situ hybridisation for *Fpr3* and the immediate early gene *Egr1* as a marker of neural activation^61^ on VNO tissue sections (Fig. 7c–f). Stimulus solution was either placed directly onto a male’s oronasal groove^60^ (Fig. 7c), or mice were allowed to freely investigate filter paper containing specific test solutions (Fig. 7d). Both types of experiments revealed *Egr1* induction after peptide exposure in ~50% of *Fpr3*-positive VSNs (Fig. 7c–f). This activation was not oberserved with control stimuli (PBS or diluted female oestrous urine, FEU), demonstrating the specificity of *Egr1* induction (Fig. 7c–f). These results show that nasal contact with MgrB peptide present in aqueous solutions activates *Fpr3*-expressing VSNs under in vivo conditions in freely behaving mice.

### MgrB peptide drives avoidance behaviour

**Fig. 8:**
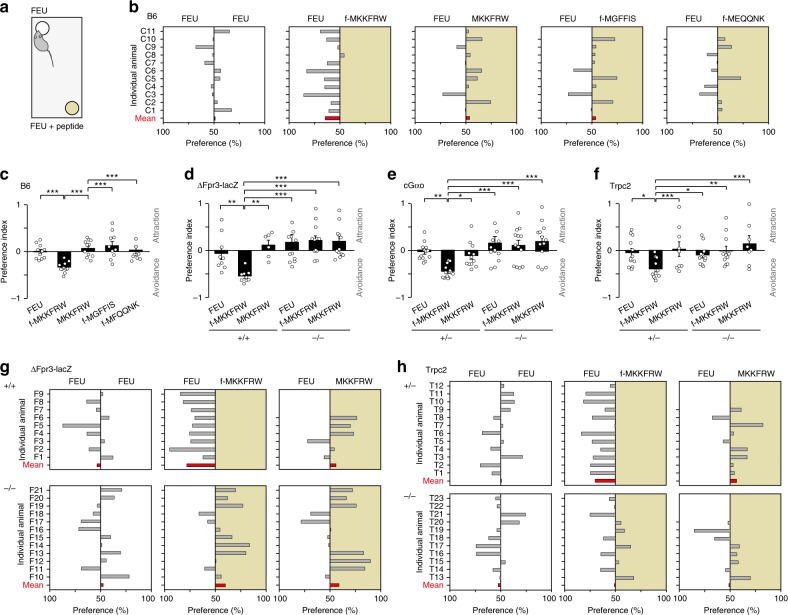
MgrB peptide drives innate avoidance. **a** Male mice were given a choice to freely investigate female oestrus urine (FEU) or FEU supplemented with peptide. Stimuli were applied on filter papers placed in randomised manner in opposite corners of the cage. **b** Shown are investigation times above 50% of individual B6 males (grey bars), illustrating animal variability. Red bars, group mean values. **c**–**f** Average responses to a given stimulus, as quantified by the avoidance index in B6 (**c**), ∆Fpr3-lacZ (**d**), cGαo (**e**), and Trpc2 mice (**f**). Data from individual mice are plotted as open circles. The avoidance index reflects avoidance by a negative value and attraction by a positive value. Data in mean ± s.e.m. (**n**): **c** B6: FEU, −0.002 ± 0.045 (11); f-MKKFRW, −0.34 ± 0.03 (11); MKKFRW, 0.07 ± 0.05 (11); f-MGFFIS, 0.13 ± 0.09 (10); f-MFQQNK, 0.04 ± 0.09 (10). ANOVA: *F*(4,52) = 7.99, *P* < 0.0001; **d** ∆Fpr3-lacZ +/+: FEU, −0.06 ± 0.13 (9); f-MKKFRW, −0.55 ± 0.04 (9); MKKFRW, 0.12 ± 0.11 (6); ∆Fpr3-lacZ −/−: FEU, 0.19 ± 0.15 (12); f-MKKFRW, 0.22 ± 0.10 (12); MKKFRW, 0.20 ± 0.12 (12). ANOVA: F(5,59) = 5.69, *P* < 0.0001; **e** cGαo +/−: FEU, −0.04 ± 0.05 (13); f-MKKFRW, −0.45 ± 0.04 (13); MKKFRW, −0.12 ± 0.08 (12); cGαo −/−: FEU, 0.15 ± 0.14 (13); f-MKKFRW, 0.10 ± 0.11 (13); MKKFRW, 0.21 ± 0.11 (13). ANOVA: *F*(5,76) = 6.53, *P* < 0.0001; **f** Trpc2+/−: FEU, −0.05 ± 0.09 (12); f-MKKFRW, −0.41 ± 0.06 (12); MKKFRW, 0.02 ± 0.16 (9); Trpc2 −/−: FEU, −0.10 ± 0.07 (11); f-MKKFRW, −0.02 ± 0.10 (11); MKKFRW, 0.14 ± 0.17 (8). ANOVA: *F*(5,62) = 3.21, *P* = 0.01. LSD: *P < 0.05, **P < 0.01, ***P < 0.001. **g**, **h** Investigation time above 50% that individual male ∆Fpr3-lacZ (**g**) and Trpc2 (**h**) mice (grey bars) examined both stimuli. Statistics were performed on average responses quantified by the avoidance index (**c**–**f**). Source data are provided as a Source Data file

Given the importance of the olfactory system in a variety of behavioural avoidance and defence mechanisms^14,62,63^, we reasoned that in vivo detection of MgrB peptide may evoke an avoidance reaction in mice. We devised a behavioural assay^14,60^ in order to determine whether direct nasal contact with MgrB peptide evokes unconditioned, innate avoidance (Fig. 8a and Methods). Using naïve male mice, we compared the effects of the Fpr3 agonist f-MKKFRW versus non-formylated MKKFRW and the two fMet peptides f-MGFFIS and f-MFQQNK which failed to activate heterologously expressed Fpr3^21^ (Fig. 8b). FEU or FEU supplemented with a given peptide were placed on filter paper in opposite corners of the testing cage and mice were given a choice to investigate the two stimulus sources. All mice showed a keen interest in investigating the stimulus source during which their nose was in close contact with the stimulus source^60^. This investigative behaviour was quantified as the time spent in close contact with each filter paper^60^.

We found that f-MKKFRW (100 µM) elicited robust avoidance, not only in B6 mice (Fig. 8b, c) but also in ΔFpr3-lacZ+/+, cGαo+/−, and Trpc2+/− mice (Fig. 8d–h and Supplementary Fig. 4). By contrast MKKFRW, f-MGFFIS and f-MFQQNK (100 µM) failed to elicit avoidance (Fig. 8b, c and Supplementary Fig. 4), attesting to the remarkable sequence-specificity of this behavioural response. All of the averaged behavioural results were fully supported by examining data from each individual mouse (Fig. 8b, g, h and Supplementary Fig. 4a). Furthermore, mice were still capable of displaying avoidance at 50 µM f-MKKFRW (Supplementary Fig. 4b–d). Thus, naïve male B6 mice display innate avoidance to f-MKKFRW, which mirrors the sequence-selectivity of Fpr3 activation.

Importantly, the avoidance to f-MKKFRW was absent in ΔFpr3-lacZ −/− mice (Fig. 8d, g), in cGαo −/− mice (Fig. 8e and Supplementary Fig. 4a), and in Trpc2 −/− mice (Fig. 8f, h). Thus, the presence of a peptide motif from a virulence factor linked to specific bacteria renders FEU much less attractive and drives innate avoidance in mice. This avoidance requires Fpr3, Gαo, and Trpc2. These results provide strong support for an involvement of f-MKKFRW-responsive VSNs in this behavioural discrimination.

### Fpr3-independent avoidance of LPS-injected mice

**Fig. 9:**
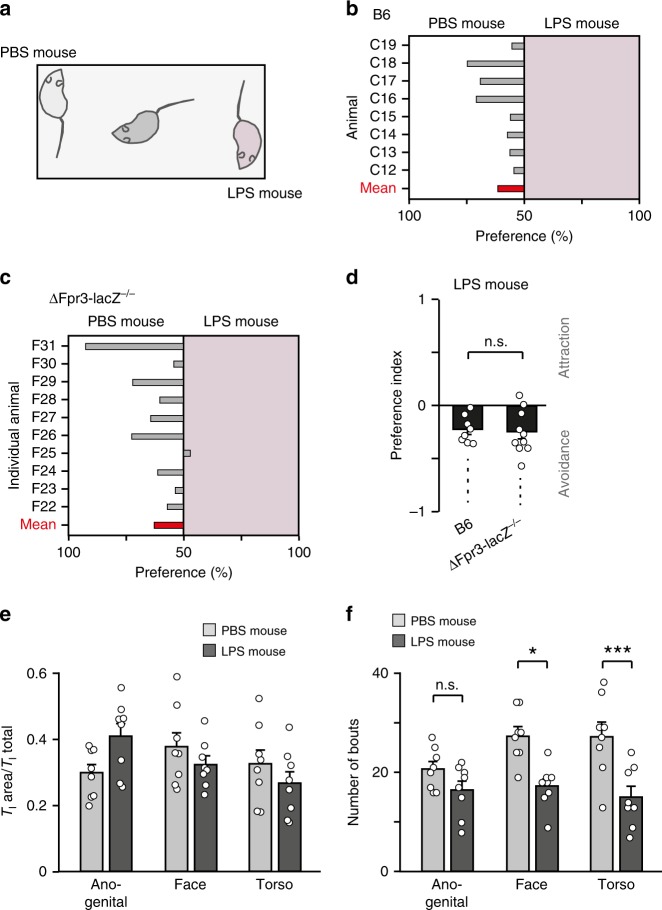
Fpr3-independent avoidance of LPS-injected mice. **a** Male B6 mice were given a choice to freely investigate an anaesthetised male mouse injected with vehicle (PBS) or LPS dissolved in PBS. Stimulus mice were placed in randomised manner in opposite corners of the cage. **b**–**d** Graphs showing investigation time above 50% that individual male B6 (**b**) and ∆Fpr3-lacZ (**c**) mice (grey bars) examined either the PBS or LPS mouse. Group mean values are plotted as red bars. The calculated avoidance index (**d**) indicates that both genotypes avoid the LPS mouse. Data in mean ± s.e.m. (n): B6, −0.22 ± 0.04 (8); ∆Fpr3-lacZ−/−, −0.25 ± 0.07 (10). Unpaired *t*-test: *t*(16) = −0.32, ns, *P* = 0.75. Data from individual mice are plotted as open circles. **e** Investigation ratio (*T*_I_ area/T_I_ total) as calculated by dividing the time investigating a particular body region (anogenital, face or torso) by overall investigation time of an anaesthetised mouse. Area: anogenital, PBS, 0.30 ± 0.03 (8), LPS, 0.41 ± 0.04 (8); face, PBS, 0.38 ± 0.04 (8), LPS, 0.32 ± 0.02 (8); torso, PBS, 0.33 ± 0.04 (8), LPS, 0.27 ± 0.04 (8). ANOVA: *F*(5,47) = 2.18, *P* = 0.08. **f** Investigation of other mice during the 10-min trial comprised high numbers of individual contacts (bouts), 75.1 ± 4.2 bouts (8) for PBS mice and 48.9 ± 3.7 bouts (8) for LPS mice. Area: anogenital, PBS, 20.6 ± 1.5 (8), LPS, 16.5 ± 1.8 (8); face, PBS, 27.3 ± 1.8 (8), LPS, 17.3 ± 1.5 (8); torso, PBS, 27.3 ± 2.8 (8), LPS, 15.1 ± 2.1 (8). ANOVA: *F*(5,47) = 7.49, *P* < 0.0001; Tukey: *P < 0.05, ***P < 0.001, ns, *P* = 0.68. Source data are provided as a Source Data file

We assessed investigative behaviour of naïve B6 males towards LPS-injected mice. We measured the time during which the nose of a B6 mouse was in close contact with anaesthetised PBS- vs. LPS-injected mice (Fig. 9a). LPS-treated mice were avoided (Fig. 9b, d), consistent with previous results^17^. This avoidance was not affected in ΔFpr3-lacZ−/− mice (Fig. 9c, d), indicating that Fpr3 was not required for this response. Therefore, additional chemoreceptors must mediate the detection of sensory cues derived from LPS-treated mice. We also used the LPS-treated mice to analyse in more detail the strategies underlying investigative inspection of sick conspecifics. This analysis revealed two important observations: First, mice sample information from the entire body of another mouse, including the face, anogenital region and torso, spending nearly equal times with each of these body regions (Fig. 9e). The presence of bacteria-associated cues on the entire body can be sensed during this investigative behaviour. Second, olfactory inspection during a 10-min trial comprised high numbers of individual contacts (bouts), on average 49 bouts with LPS mice and 75 bouts with PBS mice (Fig. 9f). As the relative stimulus concentration within the vomeronasal cavity increases with repeated contacts^64^, this strategy maximises the probablity that stimuli at low concentrations are detected during this behaviour.

Mouse VSNs can sense natural molecules ranging from a 10^–6^ dilution to a few percentages of whole urine^64^. Effective concentrations inside the VNO typically reach ~0.5–1% of that in urine for investigations using 1–3 contacts^64^. Stimulus conditions used in our in vivo experiments (Figs. 7–9 and Supplementary Fig. 4) are consistent with these findings and with the dose-dependency of our Fpr3-mediated Ca^2+^ responses measured in vitro (Fig. 2) and in native VSNs (Fig. 4). fMet peptides are found in faeces in at least micromolar concentrations^46–49^, naturally produced MgrB peptides can be secreted by bacteria at sufficient levels to cause activation of Fpr3 (Fig. 3), and EC_50_-values for longer MgrB peptide fragments are further shifted to lower concentrations (Fig. 2). Therefore, our stimulus conditions are within the physiological range. We conclude that recognition of MgrB peptides by VSNs is biologically relevant for the display of an avoidance behaviour and that this effect depends critically on the function of a single chemoreceptor, Fpr3.

## Discussion

It has been proposed that the neural mechanisms underlying infection-avoidance behaviour may form a continuum with the immune system through the sharing of signalling pathways, sites of action, and evolutionary history^2,65^. Our results are generally consistent with this hypothesis in that they show that the innate immune chemoreceptor Fpr3 is required in the olfactory system to detect and avoid peptides that are predominantly present in signal sequences of the bacterial protein MgrB, a highly conserved regulator of virulence and antibiotic resistance in *Enterobacteriaceae*. To the best of our knowledge, these results show the first function for Fpr3 in the nervous system of any species.

Several main conclusions emerge from these studies. First, Fpr3 mediates VSN detection of a relatively small subset of bacteria-derived peptide ligands. Overall, we tested 53 fMet peptides that are representative for the *N*-termini of 15,125 annotated bacterial proteins (Supplementary Data 1). On the basis of combined analyses from heterologously expressed Fpr3 and native VSNs, we conclude that the f-MKKFRW motif serves as a minimal consensus sequence for the recognition by Fpr3, and we defined some of the recognition rules by which fMet peptides are detected (Figs. 1 and 2). Relatively slight deviations from the minimal consensus sequence abolished or diminished Fpr3 activation whereas elongation of this sequence increased response amplitude and efficacy. We hypothesised previously that some immune Fprs may function as general detectors of bacterial signal sequences^21,43^. The current study indicates that Fpr3 is not a general detector of such peptides but that it recognises only a very small subset of formylated signal sequences. Signal peptides and their *N*-terminal fragments can be secreted by bacteria and are found in the extracellular medium of bacterial cultures^66,67^. Here, we provide the first evidence that MgrB-derived fMet peptides are produced by bacteria, secreted into the supernatant, and detected by Fpr3 (Fig. 3).

Second, the f-MKKFRW sequence is strongly enriched in bacteria, and is almost exclusively present in *Enterobacteriaceae* or enterobacterial isolates. One of the most intriguing results of our work is that this sequence occurs predominantly in the highly conserved bacterial protein MgrB that serves as a negative regulator of the PhoQ/PhoP two-component system^31^. This signalling system plays an essential role in the response of *Enterobacteriaceae* to the environment of their mammalian hosts and thereby serves as a master virulence regulator^34–37^. It senses host-related stress conditions^68^ (i.e. low pH, low magnesium, hyperosmic conditions, antimicrobial peptides and other factors), subsequently controls expression of a large collection of genes important for virulence, and thereby promotes bacterial survival inside innate immune cells such as macrophages^34–37^. MgrB inactivation is now recognised as a common mechanism for antimicrobial drug resistance in a clinical setting^38–41^. Hence, our finding that f-MKKFRW functions as a specific Fpr3 agonist in mouse VSNs links the activation of this receptor to the detection of conserved bacterial signalling systems that govern virulence in a variety of bacterial pathogens. We note that the term virulence is also defined as a feature of host-pathogen interaction, affecting disease severity by reducing fitness of the infected host^69^.

Third, the identification of a subset of native VSNs that detect f-MKKFRW in vitro and in vivo enabled us to investigate the detailed chemoreceptive properties of these sensory neurons. These VSNs seem to be exquisitely selective for f-MKKFRW, in marked contrast to other VSN subpopulations that recognise a wide variety of peptide ligands of MHC class 1 molecules and other urinary peptides^51,52,55^ or major urinary proteins^70^ and which exhibit combinatorial activation with overlapping specificities^52,70^. Our demonstration that f-MKKFRW-evoked responses occur in B2m-expressing VSNs is consistent with the expression of Fpr3 in a subset of cells in the basal VNE layer^25,26^, and we showed that these neuronal Ca^2+^ responses require Fpr3, Gαo and Trpc2. Together, these results indicate that Fpr3-VSNs do not detect a diverse array of chemical signatures from the vast repertoire of potential pathogenic agents^71^, but rather are specialised detectors for a limited set of bacterial peptides that represent highly conserved proteins located at critical positions in virulence regulation. We propose that Fpr3 could enable mice to sense the infective capacity of conspecifics and their bodily secretions.

Fourth, as urine from LPS-injected mice does not activate Fpr3 and mice avoid other LPS-injected mice in an Fpr3-independent manner, we suggest a model in which repulsive signals that indicate accute inflammation or illness are detected by multiple types of chemoreceptors and perhaps multiple olfactory subsystems, not unlike the parallel mechanisms employed for sensing and avoiding predator odours^14^. In fact, mice have evolved a number of mechanisms for recognising endogenous, evasive or environmental bacteria^72,73^, including trace amine-associated receptors expressed in the main olfactory epithelium^72^ and bitter taste receptors expressed in solitary chemosensory cells of the respiratory epithelium^74^. Analyses of the receptive properties of the other Fprs expressed in VSNs^25,26^ and their genetic deletion will aid in refining this model further.

Fifth, in the immune system, bacterial fMet peptides are important chemotactic molecules that attract neutrophils to infected tissue sites as a first step of host defence against invading bacteria^18–20^. By contrast, the presence of an fMet peptide in an otherwise attractive odour source (female oestrous urine) led to the display of an avoidance response in freely behaving male mice. This behavioural response showed the same sequence selectivity as f-MKKFRW-detecting VSNs, and also depended on three major signalling molecules expressed in these neurons: Fpr3, Gαo and Trpc2 (Fig. 8). Given that mice sample information from the entire body of another mouse (Fig. 9), it seems likely that the presence of MgrB peptides in urine, faeces or other bodily secretions is detected by Fpr3 under natural conditions. However, because there is presently no established MgrB infection model available, we cannot yet test this directly with endogenously produced ligands. It is known that rodents prefer to feed in locations where conspecifics have deposited urine and faeces^75^. The presence of f-MKKFRW in urine or faeces could affect the food preference of mice. Similarly, the normally attractive quality of sex pheromones in urine can be overridden by illness-associated cues^76^. Future experiments will be required to determine the entire repertoire of behavioural consequences resulting from Fpr3 activation or its genetic deletion. We can also not exclude that repeated exposure to MgrB peptides would induce a learned form of avoidance, similar to the conditioned place aversion that has been observed with other threatening chemostimuli in the olfactory system^63^.

In summary, our results have uncovered a previously unknown bacteria–nose–brain mechanism that regulates behaviour. There is currently great interest in the identification of brain circuits underlying biological threat detection and innate behavioural defence^14,77–79^. Our results pave the way to identify higher neural circuits that are activated in response to the detection of defined bacterial cues to mediate infection-avoidance behaviour.

## Methods

### Heterologous expression of Fpr3

An *Fpr3* gene subcloned from genomic DNA of C57BL/6J mice into pcDNA3.1 (Invitrogen) was used in heterologous experiments^21,80^. HEK293T (DSMZ # ACC-635) cells were seeded at 20–30% confluence on poly-D-lysine-coated (10 µg/ml in PBS) black 96-well µCLEAR-Plates (Greiner Bio-One). Cells were transfected 24 h later using jetPEI™ (Polyplus-transfection SA) according to the manufacturer’s protocol for 96-well transfections. For Ca^2+^ imaging experiments, 0.125 µg of DNA plasmid encoding Fpr3 were cotransfected with equal amounts of a plasmid encoding Gα16, a promiscuous G protein α-subunit. Ca^2+^ signals to agonist stimulation were recorded 48 h post transfection using the Ca^2+^ indicator dye Fluo-4/AM (Molecular Probes) and a fluorescence imaging plate reader (FLIPR) system (Molecular Devices). Cotransfection of a plasmid encoding Gα16 with an empty vector used to subclone *Fpr3* gave no agonist-evoked Ca^2+^ responses.

### Peptides

We used custom-made peptides with a length of 6–21 amino acids to screen for agonists. Sequences of fMet peptides were designed according to motifs present at the *N*-termini of 15,125 specific bacterial proteins as annotated in UniProt. Peptides were chemically synthesised, purified, and verified by mass spectroscopy (MALDI-TOF) by commercial suppliers. Purity of all peptides was >95%. Source, sequence and purity of each peptide are listed in Supplementary Data 1. Lyophilised peptides were routinely dissolved in the Ca^2+^ imaging assay buffer C1 (130 mM NaCl, 10 mM HEPES, 5 mM KCl, 2 mM CaCl_2_, 5 mM glucose, pH 7.2) as 0.2–1 mM stock solutions and kept in small aliquots at −20 °C until use. We used previously two peptides known as W-peptide and M-peptide as agonists for mouse Fpr3^21,80^. These two synthetic peptides do not exist in nature and are therefore not of biological relevance. We also have identified two closely related signal peptide fragments, f-MLFYFS and f-MLFYLA, as agonists for mouse Fpr3^21^. The sequences of these peptides exist in proteins from the following bacterial strains^21^: *Psychromonas ingrahamii* that belongs to a marine ice water microbial community, *Desulfotomaculum reducens* that is found in deep freshwater lakes, and *Pseudomonas grimontii*, a plant pathogen. As mice are unlikely to encounter these strains in their natural habitat, these findings are probably not of biological relevance.

### Bioinformatic analyses

To analyse the natural occurrence and abundance in microorganisms of fMet peptides used in this study, we employed the peptide search function of UniProt. Further analyses concerning sequence, taxa, and function of specific organism were performed. To carry out a disease-risk-classification of *mgrB*+ bacterial strains, we used the Risk Group Database from the American Biological Safety Association (ABSA International).

### MgrB expression in bacteria

A sequence containing the *E. coli* EDL933 *mgrB* gene (Genbank Z2872) fused to a C-terminal His-6 tag (Supplementary Fig. 1d) was designed, synthesised (Eurofins), and subcloned into the XbaI and XhoI restriction sites of pBluescript KS(-) to obtain plasmid p*mgrB-H6*. This is the *mgrB* sequence of the first EHEC isolate sequenced (strain EDL933)^81^ which is identical to the ones found in BL21 or K12. The plasmid was then transformed into *E. coli* TOP10 cells to isolate sufficient amounts of plasmid DNA and verify the sequence. For overexpression, p*mgrB-H6* was transfected into *E. coli* BL21 (DE3) pLysS (NEB) and plated on ampicillin-containing agar plates (100 µg/ml). In all, 16–24 h later, freshly grown colonies were transferred to 30 ml 1x M9 Salt Solution (Sigma-Aldrich) supplemented with 1x MEM Amino Acid Solution (Sigma-Aldrich). After 24–48 h, this bacterial culture was harvested by centrifugation and resuspended in M9 medium supplemented with the amino acid mix and 0.1 mM IPTG (Sigma-Aldrich). After different time points, 10 ml samples were collected, the bacteria were harvested by centrifugation and the supernatant was sterile filtrated using 0.2 µM filters (Filtropur S, Sarsted). All samples were immediately frozen and stored at −20 °C until use.

### SDS–PAGE and western blot

Cultures of BL21 (DE3) pLysS and BL21 (DE3) pLysS harbouring plasmid p*mgrB-H6* were grown for 7 h at 37 °C and 250 rpm in M9 medium supplemented with the amino acid mix and 0.1 mM IPTG. Cultures were subsequently adjusted to an optical density at 600 nm of 5, the bacteria sedimented by centrifugation for 5 min at 12,000 g, and bacterial pellets resuspended in 1 x SDS PAGE loading buffer (30 mM Tris-HCl, pH 6.8, 1% SDS, 12% glycine, 0,005% bromophenol blue, 2.5% β-mercaptoethanol). The bacterial solutions were incubated for 5 min at 99 °C, cooled down, and sonicated for 20 s at 50W. In all, 5 µl of the whole cell extracts, each adjusted to an optical density of 5, were loaded and separated on a 15% SDS–PAGE in Tris/glycine buffer. In total, 100 mA current for 1.5 h was applied for a semi-dry transfer of the samples on an Immobilon- P^SQ^ membrane (Thermo Fisher). The membrane was then incubated at 4 °C overnight with a polyclonal rabbit anti-6×-HIS-Tag IgG antibody (ThermoFisher PA1-983B) at a 1:750 dilution, followed by a second incubation for 1 h at RT with an HRP-conjugated goat anti-rabbit IgG (BioRad, 1706515) at a 1:3000 dilution. For detection, we used the Western LightningPlus-ECL (PerkinElmer) chemoluminescence substrate and a BioRad ChemiDoc system.

### MALDI-TOF mass spectrometry

In all, 1 ml of each sterile filtrated bacterial supernatant was acidified with 0.4 ml of 1% trifluoric acid (TFA; Applied Biosystems, Warrington, UK). The solution was loaded onto an activated C18-SPE-cartridge (100 mg; Macherey-Nagel, Düren, Germany); the retained peptides were washed with 0.1% TFA and eluted with 80% acetonitrile (Roth GmbH, Karlsruhe, Germany) in 0.1% TFA. The eluate was lyophilised overnight, redissolved in 20 µl 0.1% TFA, and 1 µl of this sample was mixed with 1 µl matrix solution (2, 5-dihydroxybenzoic acid, DHB; 5 mg/ml; Sigma, Darmstadt, Germany), and methylendiphosphonic acid (MDPA; 5 mg/ml; Fluka, Steinheim, Germany) in 0.1% TFA) for MS analysis. MALDI-TOF-MS was performed on an Ultraflex I TOF/TOF mass spectrometer (Bruker Daltonics, Bremen, Germany) equipped with a nitrogen laser and a LIFT-MS/MS facility. The instrument was operated in the positive-ion reflectron mode acquiring 200–400 single spectra per sample for sum spectra. For data processing and instrument control the Compass 1.3 software package consisting of FlexControl 2.4, FlexAnalysis 3.0, BioTools 3.0 was used.

### Mice

All experimental procedures have complied with all relevant guidelines and ethical regulations for animal testing and research established and approved by the animal welfare committees of Saarland University, The Rockefeller University, and the Max Planck Research Unit for Neurogenetics. Mice were housed under a reverse 12:12 h light/dark cycle with food and water ad libitum. Ten different mouse strains or crosses were used in this study. C57BL6/N mice (referred to as B6) served as wild-type controls and were obtained from Charles River Laboratories (Sulzfeld, Germany). Trpc2-IRES-taumCherry mice^82^ and OMP-GFP mice^83^ were used to identify VSN dendritic knobs in the VNO whole-mount preparation. The generation of B2m-Venus mice and of ΔFpr3-lacZ mice is described below. B2m-Venus mice were crossed with NP2∆ mice^56^ to yield NP2∆/B2m-Venus mice, which are heterozygous for both mutations. Some experiments also used 129Sv mice (Charles River Laboratories)^30^, conditional cGαo knockout mice^57^, and Trpc2 knockout mice^58^. Control mice were heterozygous littermates from conditional cGαo knockout mice^57^, and Trpc2 knockout mice^58^.

### Treatment of mice with LPS and urine collection

Male mice (B6, 8–12 week old) were injected intraperitoneally 4 h prior to behavioural testing with 1 mg/kg LPS (Lipopolysaccharide L4391, Sigma) diluted in PBS. Control mice were injected with 200 µl PBS. Mice were returned into their individual home cages. In total, 4 h post injection, urine from mice injected with LPS or PBS, respectively, was collected and pooled, and used either directly in Fpr3 assays or stored at −80 °C until use.

### VNO whole-mount preparation and en face Ca^2+^ imaging

Mice (6–20 week old, either sex) were euthanized by CO_2_ inhalation according to institutional guidelines. After decapitation, the skin was removed from the skull, the mandible was cut off and the cranium was opened by a sagittal cut, 1–2-mm right-lateral in parallel to the midline. The palate and the right incisor were removed and the hemisected skull was mounted in low-melting agarose (4%). The bony capsule of the VNO was opened with fine forceps and the nonsensory epithelium was stripped off. Tissue and cell debris were gently washed off, and the en face preparation was superfused with physiological S1 solution (120 mM NaCl, 25 mM NaHCO_3_, 5 mM KCl, 1 mM MgSO_4_, 1 mM CaCl_2_, 10 mM glucose, 5 mM BES, pH 7.3) saturated with carbogen (95% O_2_, 5% CO_2_).

Loading of the VNE with Rhod-2/AM (5–15 µM, 1 h) was performed in S1 solution under carbogen atmosphere at RT. Images were taken with an upright confocal laser scanning microscope (Leica TCS SP5 II, 20x water immersion objective HCX APO L20x/1.0w) equipped with Ar and He/Ne lasers. GFP was excited at 488 nm, Venus at 514 nm and Rhod-2 at 543 nm. Emission was measured at 490–520 nm (GFP), 520–600 nm (Venus) and 560–680 nm (Rhod-2). To minimise interference between Venus and Rhod-2 fluorescence, images were taken sequentially (sample interval per image, 3–4 s). Peptide stimuli were applied to the VNE surface with a local perfusion system which, when positioned close to the anterior end of the VNO, produced a continuous solution stream from rostral to caudal along the VNO axis. Data analysis was performed with the open source software ImageJ (NIH). Ca^2+^ responses of individual dendritic knobs were normalised to the knob resting fluorescence level obtained before stimulation (ratio *F*_x_/*F*_0_, *F*_x_ = actual fluorescence; *F*_0_ = mean fluorescence of 3–5 images before ligand application). Mapping was performed by superimposing Ca^2+^ peak images onto images showing intrinsic fluorescence for specific markers.

Predator urine mix was composed of urine from mountain lion, coyote, bobcat, fox and marten (at equal volume ratios), purchased from Predatorpee (USA) and Kieferle (Germany). The fMet peptides were as described above. Stimuli were applied in S2 solution (140 mM NaCl, 5 mM KCl, 10 mM HEPES, 1 mM CaCl_2_, 1 mM MgCl_2_, 10 mM glucose, pH 7.3 with NaOH).

### Density of VSN knobs in VNO whole-mount preparation

For calculations of VSN knob density, we analysed randomly selected VNE areas, each containing several hundred knobs. The mean density of OMP-GFP+ knobs was 11.25 knobs/100 µm². Through the knob density and the VNE surface area, an estimate of the total number of VSNs per VNO can be made. Assuming a mean lumen diameter of 450 µm, a length of 2 mm and an end-to-end semilunar cross-sectional shape of the VNO, a number of ~150,000 VSNs (per side) can be estimated. This number is consistent with our previous estimates^52^.

### Gene targeting of *B2m* locus and validation of B2m-Venus mice

In B2m-Venus mice, coding exon 1 of the *B2m* gene is replaced with a cassette composed of *Venus*, *rabbit ß-globin poly-A signal* and *ACNf*. The targeted mutation deletes 64 of 67 nt from the exon 1 coding sequence. The targeting vector was electroporated into E14 ES cells^83^. Mice are in a mixed 129 x C57BL/6 background. B2m-Venus mice are publicly available from The Jackson Laboratory as stock JR#7888 and B6;129P2-B2m<tm1(Venus)Mom>/MomJ.

### Generation of ΔFpr3-lacZ mice

Generation of these mice was accomplished by utilising *Fpr3*-targeted ES cells from the European Union Conditional Mouse Mutagenesis program. The mouse strain Fpr3<tm1b(EUCOMM)Hmgu> (MGI:5584405) was generated by the Mouse Clinical Institute (Illkirch, France)^84^. Mice are in a C57BL/6N background. In this strain, the *Fpr3* coding sequence has been replaced by an *E. coli LacZ* reporter gene using the Cre-*loxP* system (Fig. 5a). A L1L2_Bact_P cassette was inserted at position 17970088 of Chromosome 17 upstream of the exon encoding *Fpr3*. The cassette is composed of an *FRT* site followed by the *lacZ* sequence encoding the bacterial enzyme β-galactosidase and a *loxP* site (L1). This first *loxP* site is followed by the neomycin resistance gene under the control of the human *ß-actin* promoter, *SV40* polyA, a second *FRT* site, and a second *loxP* site (L2). A third *loxP* site (L3) was inserted downstream of the *Fpr3* coding exon at position 17,971,914. Subsequent *Cre* expression through crossing with a global deleter mouse line created an Fpr3 knockout/lacZ-knockin mouse that we call ΔFpr3-lacZ. Experiments were performed on +/+, +/− or −/− littermates.

### PCR analyses of ΔFpr3-lacZ mice

For genotyping of ΔFpr3-lacZ mice, we prepared genomic DNA extracts from 1-mm tail biopsies by incubating tissue in digestion buffer (50 mM Tris HCl pH 8.0, 20 mM NaCl, 1 mM EDTA, 1% SDS, 1 mg/ml proteinase K) for 60 min at 56 °C, followed by proteinase K inactivation for 20 min at 95 °C. In all, 1 µl DNA extract was subjected to 25 µl PCR reaction using the *GoTaq* G2 DNA Polymerase system (Promega) and gene-specific primers (10 pmol each). Primers were 833-Ef: GTCTGTTATTCATTCTGTGGGTCATGC, 834-Kr: CCAACAGCTTCCCCACAACGG, 831-L3f: GAAGGCCTTAGACTCACTGGGTTAG, 832-L3r: CCCAGATGCCCTTCAACAGAGG (Eurofins Genomics, Ebersberg, Germany). Cycling parameters were an initial denaturation [3 min, 95 °C], followed by 36 cycles of [30 s, 95 °C – 30 s, 65 °C – 60 s, 72 °C], and a final extension [10 min, 72 °C] prior to cooling samples to 4 °C. Correct sizes of PCR products were analysed on 1.5% agarose gels containing ethidium bromide. Primer combinations were 833-Ef / 834-Kr yielding a 417-bp amplicon for the mutant allele, and 831-L3f / 832-L3r yielding a 397-bp amplicon for the wild-type allele.

For RT-PCR analyses, RNA from whole VNO tissue of adult mice was obtained with the innuPREP RNA Mini Kit (Analytik Jena AG, Jena, Germany) according to the manufacturer’s protocol. Quality was assessed by gel electrophoresis and photometric measurements. cDNA was synthesised from 0.5 µg of total RNA using Smart cDNA Synthesis protocol (Clontech) and Superscript II Reverse Transcriptase (Invitrogen). PCR was performed with Phusion High Fidelity DNA polymerase (New England Biolabs Inc.) according to the manufacturer’s protocol. Primer sequences and annealing temperature were: for mFpr3: ATGGAATCCAACTACTCCATCCATCT and TATTGCCTTTATTTCAATGTCTTCAGGAAGT, 64 °C; for mFpr-rs3: ATGGAAGCC AACTCCTCCATC and TAGTTCAGAGTCGGCAGGACATGA, 64 °C; for mFpr-rs4: ATGGAAGTCAACATTTCAATGCCTCT, GTCTTCCCTCAGGGCCCTCTC and 64 °C; for mFpr-rs6: ATGGAAGCCAACTTCTCCATACCTC and GAGTCTTTGTGAAGACAAGTTTCTG, 64 °C; for mFpr-rs7: ATGGAAGCCAACTTCTCCATACCTC and GAGTCTTAAGTTTGTGAAGACAAGTTTCTGATTT. Primers covered the complete coding region of the indicated genes. Specificity of the PCR products was confirmed by direct sequencing of gel-purified PCR products.

### Immunohistochemistry

Mice were anaesthetised by intraperitoneal injection of 165 mg/kg body weight ketamine (Pharmacia GmbH, Berlin, Germany) and 11 mg/kg body weight xylazine (Bayer Health Care, Leverkusen, Germany), and sacrificed by transcardial perfusion with ice-cold phosphate buffered saline (PBS), followed by 4% paraformaldehyde (PFA) in PBS. Mouse heads were dissected, fixated in 4% PFA for 2 h, cryoprotected in 30% sucrose in PBS at 4 °C for 2 days, embedded in O.C.T. (Tissue-Tek), and snap-frozen in a dry ice/2-methylbutane bath. Adjacent sets of coronal tissue sections (12 µm) of the whole VNO were collected on a cryostat (HM525; Microm, Walldorf, Germany), thaw-mounted onto glass slides (Superfrost Plus, Polysciences), and stored at −80 °C. For anti-β-gal immunohistochemistry, sections were treated with 3% H_2_O_2_ for 10 min, washed with PBS, incubated in blocking buffer containing 0.2% Trion X-100 (Sigma), 4% normal donkey serum (Vector Laboratories), prepared in PBS for 60 min. If not otherwise noted, incubation steps were at room temperature. Sections were incubated overnight at 4 °C in polyclonal rabbit anti-β-gal primary antibody (5-Prime 3-Prime; Boulder, CO; RRID:AB_2314507) diluted 1:500 in blocking buffer. After three washes for 10 min in PBS, sections were incubated in biotinylated goat anti-rabbit serum (BA-1000, Vector Laboratories; RRID:AB_2313606) for 1 h, then washed 3 × 10 min in PBS, incubated in Avidin/Biotin Complex (VECTASTAIN Elite ABC-Peroxidase Kit, Vector Laboratories; RRID:AB_2336817) for 90 min, and washed in PBS. Bound antibody was visualised with 0.05 g/L DAB (Sigma-Aldrich) and 0.015% H_2_O_2_ in PBS. Then sections were dehydrated in graded ethanol, incubated in Roti-Histosol (Carl Roth, Germany) for 10 min, and mounted in DPX permanent medium (Sigma-Aldrich, Germany). Bright-field images were acquired on a BX61 microscope attached to a DP71 camera (Olympus) and minimally adjusted in contrast and brightness using Adobe Photoshop Elements 10.

### RNAscope fluorescence in situ hybridisation

For each mouse, serial coronal VNO cryosections (12 µm) were collected as two alternate sets, each representing every second section of the whole VNO. One set was subjected to RNAscope in situ hybridisation using the RNAscope Fluorescent Multiplex Detection Kit (ACD Bio-Techne) and specific probes for *Fpr3* and *LacZ* according to the manufacturer’s protocol. Sections were incubated in 1 x citrate buffer pH 6.0 (DAKO, Germany) for 5 min at 98 °C in a coplin jar, rinsed twice in distilled water, dehydrated in 100% ethanol, and air dried prior to protease III treatment for 30 min at 40 °C. After rinsing in distilled water, sections were simultaneously hybridised with RNA scope target probes generated for mouse *Fpr3* and E.coli *LacZ* (ACD Bio-Techne). The *Fpr3* probe was designed as channel 1 probe containing 7 ZZ structures targeting base pairs 456–1143 of NM_008042.2, specific to mouse *Fpr3*. The *LacZ* probe, designed as channel 3 probe contains 30 ZZ structures targeting the region 279–1790 bp of KC847299.1 (cat# 313451-C3). The *LacZ* probe (50 x stock solution) was 1:50 diluted in the supplied 1×*Fpr3* solution. Following hybridisation (2 h, 40 °C), sections were rinsed twice for 2 min in wash buffer (ACD Bio-Techne), and sequentially treated with amplification solutions at 40 °C (30 min Amp1-FL, 15 min Amp2-FL, 30 min Amp3-FL) and fluorescence reagent (15 min, 40 °C Amp4-altC), with intermitting washing steps at room temperature between reagents. Cell nuclei were stained with DAPI solution (ACD Bio-Techne) for 1 min, and tissue sections were mounted with DAKO fluorescence medium and stored for 12 h at 4 °C. Fluorescence images of the RNAscope results were acquired from each section using a Zeiss LSM 880 confocal microscope containing a 32-channel GaAsP-PMT and 2-channel PMT QUASAR detector.

To quantify numbers of *Fpr3-* and *LacZ-*labelled VSNs, every second section throughout the whole extent of the VNO was scanned and manually counted. Counts derive from four B6 mice (250 VNO sections), three ΔFpr3-lacZ +/− mice (200 VNO sections) and three ΔFpr3-lacZ −/− mice (158 VNO sections).

### Assessing activation of *Fpr3*-expressing VSNs in vivo

We stimulated adult male B6 mice with f-MKKFRW (100 µM) and performed double-labelling RNAscope in situ hybridisation for *Fpr3* and the immediate early gene *Egr1* on VNO tissue sections. We applied 25 µl of 100 µM f-MKKFRW dissolved in PBS (peptide) or 25 µl of PBS (control) to the oronasal groove of mice that were previously kept in sterilised cages without bedding for at least 1 h. At 40 min post-stimulation, mice were killed by transcardial perfusion, and VNO tissue was prepared for RNAscope analyses. Results were verified using an experimental setup employing freely behaving mice. Habituation of mice on four consecutive days was as described below for other behavioural tests. Following habituation on day 4, mice were individually placed into a new cage containing a petri dish with a filter paper carrying either 45 µl 50% female oestrous urine (FEU, diluted with PBS) + 5 µl C1 solution (control), or 45 µl 50% FEU (diluted with PBS) + 5 µl f-MKKFRW (100 µM final concentration). Direct physical contact with the filter paper was allowed for 10 min; 40 min later mice were killed by transcardial perfusion. VNO tissue was prepared for RNAscope fluorescence in situ hybridisation as described above using *Fpr3* and *Egr1* probes simultaneously. The *Egr1* probe, designed as channel 3 probe contains 20 ZZ structures targeting base pairs 244–1241 of NM_007913.5, specific to mouse *Egr1* (ACD Bio-Techne, cat# 423371-C3). The *Egr1* probe (50 x stock solution) was 1:50 diluted in the supplied 1×*Fpr3* solution. To quantify peptide-mediated VSN activation, *Fpr3-*labelled VSNs deriving from 15–25 VNO sections per mouse were evaluated for co-labelling with *Egr1*. Labelled VSNs were scanned using a Zeiss LSM 880 confocal microscope and manually counted. Colocalization of *Fpr1* and *Egr1* is expressed as fraction of *Fpr3*-labelled VSNs.

### Behavioural testing

These assays were adapted from behavioural procedures described previously^14,60^. We tested avoidance by giving adult male mice (8–14 week old) of various genotypes a choice to freely investigate two types of chemical stimuli: FEU vs. FEU supplemented with a peptide. Mice were sexually naïve and not exposed to the test stimuli prior to the testing day. Mice were habituated for three days (40 min each day) to clean filter papers (2.5 cm diameter), each deposited in a sterile plastic culture dish and placed in opposite corners of the test cage (30 × 12 cm). After the third habituation period, the test subject was transferred into a new cage containing fresh filter papers/dishes with either of the two test stimuli. One filter paper was impregnated with 45 µl of fresh urine pooled from two sexually naïve female B6 mice (8–16 weeks old) in oestrus (FEU, female oestrous urine) and 5 µl physiological solution containing (in mM): NaCl, 130; KCl, 5; Na-Hepes, 10; CaCl_2_, 2; glucose, 10; pH = 7.4. For testing the effects of specific peptides, the second filter paper was impregnated with FEU supplemented with a given peptide (100 or 50 µM final concentration, dissolved in physiological solution). Direct physical contact was allowed. Behaviour was recorded for 10 min from the top of the cage. Mice were exposed only once to a given peptide solution, and were exposed to only one peptide stimulus per day. Stimuli were presented in randomised order. All mice showed a keen interest in investigating the stimulus source during which their nose was in close contact with the stimulus source^60^. This investigative behaviour was quantified as the time spent in close contact with each filter paper, scored manually by an observer blind to the experimental conditions using Behavioural Observation Research Interactive Software (BORIS)^85^. Contact time was measured as the time during which the nose crossed the border of a petri dish containing impregnated filter paper. An avoidance index was calculated as (T_P_ — aT_U_)/(aT_P_ + aT_U_), with T_P_ as the time an individual mouse investigated a FEU-peptide mixture, aT_P_ as the average time of all tested mice investigating this stimulus and aT_U_ as the average time of all mice investigating FEU without added peptide. Negative values represent avoidance and positive values represent attraction. For analysing investigation of sick conspecifics, B6 mice were injected either with LPS or PBS as described above. In total, 4 h post injection, these mice were anaesthetised with 165 mg/kg ketamine (Pharmacia GmbH, Berlin, Germany) and 11 mg/kg xylazine (Bayer Health Care, Leverkusen, Germany) and then used in behavioural tests.

### Statistical analysis

Statistical analyses were performed using the software packages SPSS (IBM Corporation, New York, U.S.A.) or Origin Pro 2017G (OriginLab Corporation, Northampton, MA, USA). Assumptions of normality and homogeneity of variance were tested before conducting the following statistical tests. Student’s *t*-test was used to measure the significance of the differences between two distributions. Multiple groups were compared using a two-way analysis of variance (ANOVA) with Tukey’s multiple comparison or LSD test as a posthoc comparison. In case the results failed the test of normality, the Kruskal–Wallis ANOVA and Mann–Whitney test were performed. The probability of error level (alpha) was chosen to be 0.05. The statistical tests used were two-sided. Unless otherwise stated, data are expressed as mean ± s.d.

## Supplementary information

Supplementary Information

Description of Additional Supplementary Files

Supplementary Data 1

Supplementary Data 2

Supplementary Data 3

Supplementary Data 4

Source Data

## Data Availability

Raw data and analysis code are available from the corresponding author on request. MALDI-TOF spectra are available at 10.6084/m9.figshare.9884090 and 10.6084/m9.figshare.9884105. Source data underlying Figs. 1–4 and Figs. 6–9 are available as a Source Data file.
