# Supplementary material for: Bacterial MgrB peptide activates chemoreceptor Fpr3 in mouse accessory olfactory system and drives avoidance behaviour
Source: Nat Commun. 2019 Oct 25;10:4889. doi: 10.1038/s41467-019-12842-x (PMC6814738; doi:10.1038/s41467-019-12842-x)
Supplement: Supplementary file 1 — Supplementary Information [file 41467_2019_12842_MOESM1_ESM.pdf]

Supplementary Information for

Bacterial MgrB peptide activates chemoreceptor Fpr3 in mouse accessory olfactory system and drives avoidance behaviour

Bufe et al.

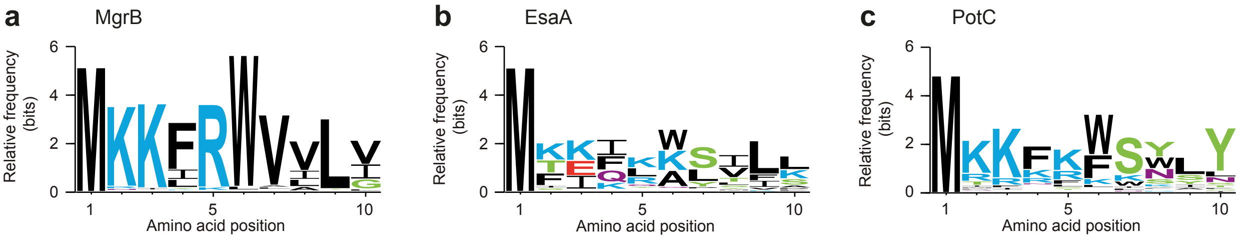

d

```
                                                XbaI
  1  AGTAAAACGACGGCCAGTGAGCTAGGTACCATCTAGAAATAATTTTGTTTAACTTTAAGA  60
     ------------------------------------------------------------
     TCATTTTGCTGCCGGTCACTCGATCCATGGTAGATCTTTATTAAAACAAATTGAAATTCT

 61  AGGAGATATACCATGAAAAAGTTTCGATGGGTCGTTCTGGTTGTCGTGGTGTTGGCTTGC  120
     ------------------------------------------------------------
     TCCTCTATATGGTACTTTTTCAAAGCTACCCAGCAAGACCAACAGCACCACAACCGAACG
                  M  K  K  F  R  W  V  V  L  V  V  V  V  L  A  C

121  TTGCTGCTTTGGGCGCAGGTATTCAACATGATGTGCGATCAGGATGTACAATTTTTCAGC  180
     ------------------------------------------------------------
     AACGACGAAACCCGCGTCCATAAGTTGTACTACACGCTAGTCCTACATGTTAAAAAGTCG
      L  L  L  W  A  Q  V  F  N  M  M  C  D  Q  D  V  Q  F  F  S

181  GGAATTTGTGCCATTAACCAGTTTATCCCGTGGGGATGCGGACACCACCACCACCACCAC  240
     ------------------------------------------------------------
     CCTTAAACACGGTAATTGGTCAAATAGGGCACCCCTACGCCTGTGGTGGTGGTGGTGGTG
      G  I  C  A  I  N  Q  F  I  P  W  G  C  G  H  H  H  H  H  H

                          XhoI
241  TGATAAGGAGAGCTAGCACTCGAGCACATGGTCATAGCTGTTTCCA  286
     ----------------------------------------------
     ACTATTCCTCTCGATCGTGAGCTCGTGTACCAGTATCGACAAAGGT
      *  *
```

Supplementary Figure 1 | a-c, Comparison of sequence logos from the first 10 amino acid residues of all 350 mgrB+ Enterobacteriaceae (**a**) with the N-termini of EsaA (secretion accessory factor) from 319 Bacillus strains (**b**) and the ABC transporter PotC (spermidine/putrescine transport system permease) from 79 Enterococcus strains (**c**). Note that the pan-species conservation of N-termini of MgrB among Enterobacteriaceae is much higher than the N-terminus conservation of the two other proteins within a single genus. Amino acids are color-coded according to their chemical properties: black, hydrophobic (A, V, L, I, P, W, F, M); blue, basic (K, R, H); green, polar (G, S, T, Y, C); purple, neutral (Q, N); red, acidic (D, E). **d**, Sequence of the E. coli mgrB-H6 construct of the E. coli O157:H7 strain. Start (ATG) and stop codons (*) are highlighted in red. The resulting amino acid sequence is displayed in one letter code (gold metallic). Restriction enzyme sites for XbaI and XhoI used for cloning are underlined.

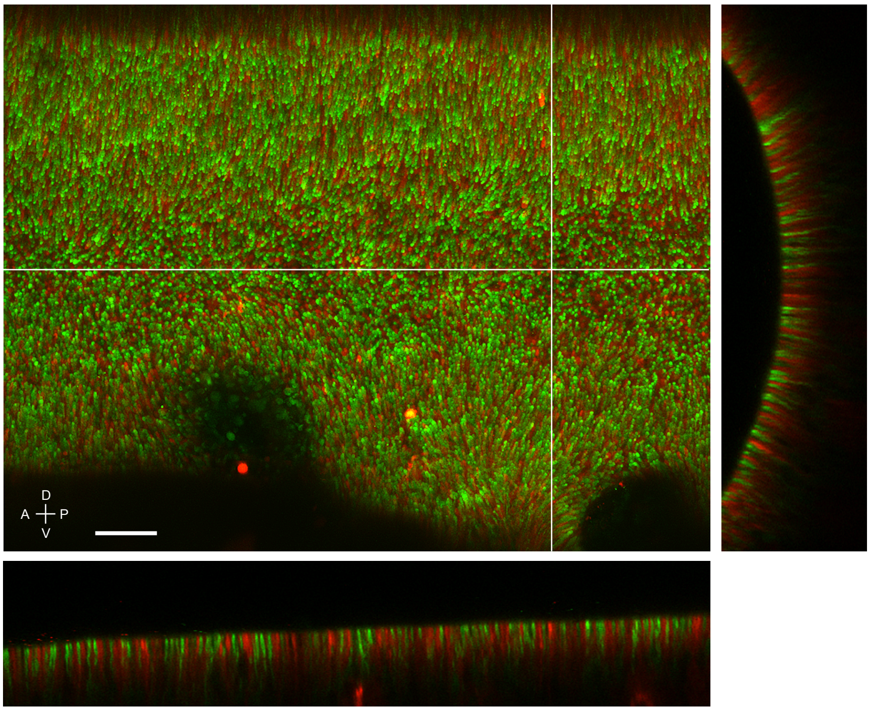

Supplementary Figure 2 | Confocal image of an en-face view of the VNE surface from a NP2 Δ/B2m-Venus mouse showing the distribution of VSN dendritic knobs that express GFP (green) or B2m-Venus (red). Optical xyz-sections were merged to obtain the high-resolution xy-image with a maximal thickness of 118 µm. Orthogonal confocal sections (bottom: xz or right: yz) demonstrate a mosaic distribution of GFP- and Venus-expressing VSN dendritic knobs. The white lines indicate the position of the two orthogonal sections. Scale bar, 50 µm. D, dorsal; V, ventral; A, anterior; P, posterior.

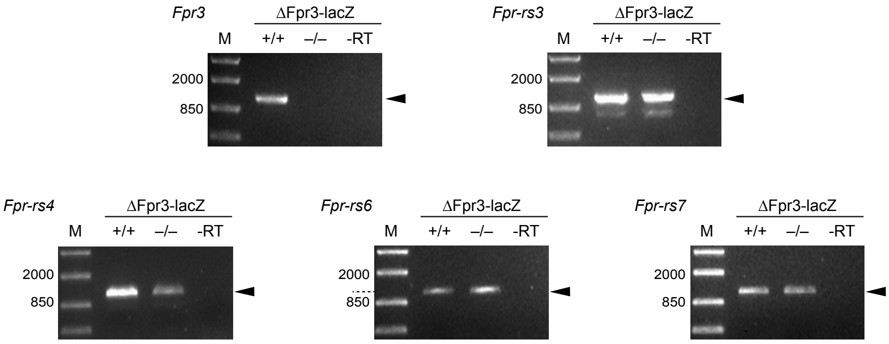

Supplementary Figure 3 | RT-PCR analyses of Fpr3, Fpr-rs3, Fpr-rs4, Fpr-rs6 and Fpr-rs7 expression in VNO tissue obtained from ΔFpr3-lacZ +/+ and –/– mice. For Fpr3, bands of correct size and sequence were present in +/+ but absent in –/– VNO. For the other vomeronasal Fprs, bands of correct size and sequence were observed in cDNA from both +/+ and –/– VNO. Genomic DNA contamination was ruled out by control reactions omitting reverse transcriptase (−RT). M, DNA size marker in base pairs (bp). Arrows denote the size of specific PCR products.

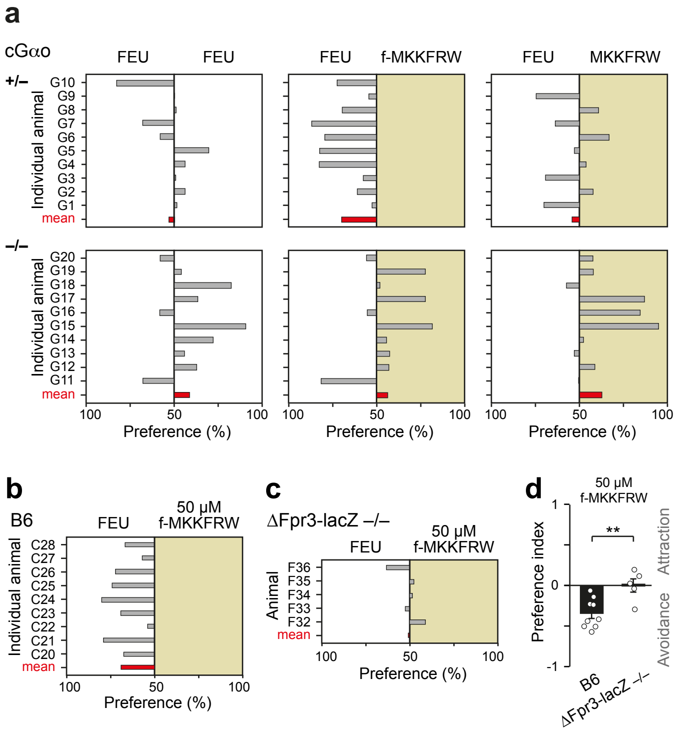

Supplementary Figure 4 | a, Investigation time above 50% that individual male cGαo mice (grey bars) spent investigating either FEU or FEU supplemented with 100 µM peptide. Group mean values (red bars) reveal the preference to a particular stimulus. **b,c**, Graphs showing investigation time above 50% that individual male B6 (**b**) and ΔFpr3-lacZ –/– (**c**) mice (grey bars) spent investigating either FEU or FEU supplemented with 50 µM f-MKKFRW. Group mean values are shown in red bars. d, Avoidance index of B6 and ΔFpr3-lacZ –/– mice calculated from the individual data shown in b and c. Data are in mean ± s.e.m. (N): B6, -0.34 ± 0.06 (9); ΔFpr3-lacZ –/–, 0.005 ± 0.08 (5). Unpaired t-test: $t(12) = 3.37$, $^{**}P < 0.01$. Data from individual mice are plotted as open circles. Source data are provided as a Source Data file.
