# Supplementary material for: Bacterial MgrB peptide activates chemoreceptor Fpr3 in mouse accessory olfactory system and drives avoidance behaviour
Source: Nat Commun. 2019 Oct 25;10:4889. doi: 10.1038/s41467-019-12842-x (PMC6814738; doi:10.1038/s41467-019-12842-x)
Supplement: Supplementary file 2 — Description of Additional Supplementary Files [file 41467_2019_12842_MOESM2_ESM.pdf]

Description of Additional Supplementary Files

File Name: Supplementary Data 1

Supplementary Data 1 | List of all synthesized fMet peptides (n=53) used in this study. These sequences are representative for the *N*-termini of 15,125 bacterial proteins. Peptides #1–41 were used in the screen of Fig. 1a. Columns display amino-acid sequence, signal peptide (SP) name, the number of naturally occurring bacterial proteins starting with this sequence at their *N*-termini, and source and purity.

File Name: Supplementary Data 2

Supplementary Data 2 | List of organisms (n=417, UniProt) that encode proteins comprising a MKKFRW motif. Columns display species name, species class, protein name, accession, and position of the MKKFRW motif within the protein.

File Name: Supplementary Data 3

Supplementary Data 3 | List of organisms (n=350, UniProt) with a full-length MgrB protein. Columns display species name, accession, disease-risk-classification according to ABSA International Risk Group Database, complete protein sequence of MgrB, and number of residues.

File Name: Supplementary Data 4

Supplementary Data 4 | Clinical relevance of the most frequent MgrB-encoding bacterial species. Columns display genera and most abundant species, stereotypical sequences of complete MgrB proteins, and general pathogenicity of these bacteria.
