# Supplementary material for: Bacterial MgrB peptide activates chemoreceptor Fpr3 in mouse accessory olfactory system and drives avoidance behaviour
Source: Nat Commun. 2019 Oct 25;10:4889. doi: 10.1038/s41467-019-12842-x (PMC6814738; doi:10.1038/s41467-019-12842-x)
Supplement: Supplementary file 3 — Supplementary Data 1 [file 41467_2019_12842_MOESM3_ESM.pdf]

Supplementary Data 1 | List of all synthesized fMet peptides (n=53) used in this study.

	Structure	Reference name	Number of bacterial proteins containing peptide sequence at N-terminus	Source and Purity
1	f-MGFFIS	SP1	71	VCPBIO, >95.43%
2	f-MKNFKG	SP2	191	VCPBIO, >96.42%
3	f-MFIYYCK	SP3	3	VCPBIO, >97.24%
4	f-MAMKKL	SP4	932	VCPBIO, >96.14%
5	f-MVMKFK	SP5	23	VCPBIO, >95.74%
6	f-MLFKYS	SP7	18	VCPBIO, >95.18%
7	f-MPKLNR	SP10	326	United biosystems, >95.32%
8	f-MVKIIF	SP11	92	United biosystems, >96.63%
9	f-MNKKLL	SP12	3036	United biosystems, >95.21%
10	f-MKKNLV	SP13	552	United biosystems, >95.67%
11	f-MEQQNK	SP14	70	United biosystems, >95.72%
12	f-MVPISI	SP15	22	United biosystems, >95.85%
13	f-MKKFLL	SP16	4819	VCPBIO, >95.96%
14	f-MMKMEG	SP17	5	United biosystems, >96.38%
15	f-MKKIML	SP18	1600	United biosystems, >95.27%
16	f-MKFCTA	SP19	7	United biosystems, >95.44%
17	f-MTNKIS	SP20	134	United biosystems, >95.91%
18	f-MKTSIR	SP21	185	United biosystems, >96.04%
19	f-MKKFNI	SP22	372	VCPBIO, >95.30%
20	f-MINKKI	SP23	394	VCPBIO, >95.38%
21	f-MLFKYL	SP24	28	VCPBIO, >96.40%
22	f-MKKFRW	SP25	326	VCPBIO, >98.34%
23	f-MEFVAKLFK	SP26	17	VCPBIO, >98.76%
24	f-MAQDIISTI	SP27	11	VCPBIO, >95.29%
25	f-MKKYKRLLL	SP28	84	VCPBIO, >98.87%
26	f-MKKTLSLKN	SP29	7	VCPBIO, >96.64%

27	f-MKKISKFLP	SP30	1	VCPBIO, >95.12%
28	f-MKKLLNKIV	SP31	10	VCPBIO, >95.20%
29	f-MLNKKKRGN	SP32	29	VCPBIO, >95.22%
30	f-MPSTFF	SP33	65	VCPBIO, >95.21%
31	f-MNPITL	SP34	203	VCPBIO, >95.33%
32	f-MLKIIL	SP35	351	VCPBIO, >95.63%
33	f-MNIFTT	SP36	77	VCPBIO, >95.42%
34	f-MAYPFQ	SP37	6	VCPBIO, >97.32%
35	f-MTHQTH	SP38	87	VCPBIO, >95.74%
36	f-MTNMRK	SP39	32	VCPBIO, >95.33%
37	f-MPQLDT	SP40	38	VCPBIO, >98.37%
38	f-MKKWRW	SP41	19	VCPBIO, >97.62%
39	f-MKKHRW	SP42	35	VCPBIO, >95.44%
40	f-MEFVAK	SP43	29	VCPBIO, >98.62%
41	f-MKKFYS	SP44	387	VCPBIO, >97.18%
42	f-MLFYLA	SP8	29	VCPBIO, >97.04%
43	f-MKKFRY	SP45	83	VCPBIO, >96.41%
44	f-MKKFLW	SP46	215	VCPBIO, >99.89%
45	f-MKKFKWV	SP47	2	Synpeptide, >98.76%
46	f-MKKFRWS	SP48	35	Synpeptide, >98.45%
47	f-MKKFRWA	SP49	8	Synpeptide, >96.65%
48	f-MKKFKWSI	SP50	102	Synpeptide, >98.14%
49	f-MKKFRWV	SP51	257	VCPBIO, >95.03%
50	f-MKKFRWVV	SP52	221	Synpeptide, >97.51%
51	f-MKKFRWVVL	SP53	221	Synpeptide, >98.49%
52	f-MKKFRWVVLGIV	SP54	117	Genscript, >95,7%
53	f-MKKFRWVVLGIVVVVCLLLWAQ	SP55	114	Peptides&elephants, >95.7%
