# Supplementary material for: Bacterial MgrB peptide activates chemoreceptor Fpr3 in mouse accessory olfactory system and drives avoidance behaviour
Source: Nat Commun. 2019 Oct 25;10:4889. doi: 10.1038/s41467-019-12842-x (PMC6814738; doi:10.1038/s41467-019-12842-x)
Supplement: Supplementary file 4 — Supplementary Data 2 [file 41467_2019_12842_MOESM4_ESM.pdf]

Supplementary Data 2 | List of organisms (n=417, UniProt) that encode proteins comprising a MKKFRW motif.

	Strain	Species class	Protein name	Accession	MKKFRW position
1	Alteribacillus bidgolensis	Bacteria	Iron(III) transport system substrate-binding protein	A0A1G8MKI2_9BACI	1-6
2	Bacillus selenatarsenatis SF-1	Bacteria	Glycerol-3-phosphate ABC transporter, periplasmic glycerol-3-phosphate-binding protein	A0A0A8X636_9BACI	1-6
3	Bacillus subterraneus	Bacteria	Uncharacterized protein	A0A0D6Z769_9BACI	18-24
4	Bacillus thuringiensis serovar cameroun	Bacteria	Type VII secretion protein EsaA	A0A242WMJ0_BACTU	1-6
5	Bacillus thuringiensis serovar malayensis	Bacteria	Type VII secretion protein EsaA	A0A242X6I4_BACTU	1-6
6	Bacillus toyonensis	Bacteria	Type VII secretion protein EsaA	A0A2B6A1I7_9BACI	1-6
7	Bacillus toyonensis	Bacteria	Type VII secretion protein EsaA	A0A2B6B693_9BACI	1-6
8	Bacillus toyonensis	Bacteria	Type VII secretion protein EsaA	A0A2C4HAP9_9BACI	1-6
9	Bacillus toyonensis	Bacteria	Type VII secretion protein EsaA	A0A2C4U1U7_9BACI	1-6
10	Bacillus toyonensis	Bacteria	Type VII secretion protein EsaA	A0A2B5Q3R7_9BACI	1-6
11	Bacillus toyonensis	Bacteria	Type VII secretion protein EsaA	A0A2B6L7H0_9BACI	1-6
12	Bacillus toyonensis	Bacteria	Type VII secretion protein EsaA	A0A2C4N501_9BACI	1-6
13	Bacillus toyonensis	Bacteria	Type VII secretion protein EsaA	A0A2C4MSA8_9BACI	1-6
14	Bacillus toyonensis	Bacteria	Type VII secretion protein EsaA	A0A2B6M5D5_9BACI	1-6
15	Bacillus toyonensis	Bacteria	Type VII secretion protein EsaA	A0A2B6MSM7_9BACI	1-6
16	Bacillus toyonensis	Bacteria	Type VII secretion protein EsaA	A0A2C3RLL9_9BACI	1-6
17	Bacillus toyonensis	Bacteria	Type VII secretion protein EsaA	A0A2C4KHF1_9BACI	1-6
18	Bacillus toyonensis	Bacteria	Type VII secretion protein EsaA	A0A2B7DTN1_9BACI	1-6
19	Bacillus toyonensis	Bacteria	Type VII secretion protein EsaA	A0A2C4VA77_9BACI	1-6
20	Bacillus toyonensis	Bacteria	Type VII secretion protein EsaA	A0A2B5SU60_9BACI	1-6
21	Bacillus toyonensis	Bacteria	Type VII secretion protein EsaA	A0A2C3TNG6_9BACI	1-6
22	Bacillus toyonensis	Bacteria	Type VII secretion protein EsaA	A0A2C5I622_9BACI	1-6

23	Bacillus toyonensis	Bacteria	Type VII secretion protein EsaA	A0A2C5IWV3_9BACI	1-6
24	Bacillus toyonensis	Bacteria	Type VII secretion protein EsaA	A0A2B7D5S4_9BACI	1-6
25	Bacillus toyonensis	Bacteria	Type VII secretion protein EsaA	A0A2C5HLL5_9BACI	1-6
26	Bacillus toyonensis	Bacteria	Type VII secretion protein EsaA	A0A2C5Q9M2_9BACI	1-6
27	Bacillus toyonensis	Bacteria	Type VII secretion protein EsaA	A0A2C4CTX3_9BACI	1-6
28	Bacillus toyonensis	Bacteria	Type VII secretion protein EsaA	A0A2B6P4U4_9BACI	1-6
29	Bacillus toyonensis	Bacteria	Type VII secretion protein EsaA	A0A2B6EL64_9BACI	1-6
30	Bacillus toyonensis	Bacteria	Type VII secretion protein EsaA	A0A2B5JPY1_9BACI	1-6
31	Bacillus toyonensis	Bacteria	Type VII secretion protein EsaA	A0A2B6D7T6_9BACI	1-6
32	Bacillus toyonensis	Bacteria	Type VII secretion protein EsaA	A0A2B7DUM3_9BACI	1-6
33	Bacillus toyonensis	Bacteria	Type VII secretion protein EsaA	A0A2B5TIC7_9BACI	1-6
34	Bacillus toyonensis	Bacteria	Type VII secretion protein EsaA	A0A2C4EPA4_9BACI	1-6
35	Bacillus toyonensis	Bacteria	Type VII secretion protein EsaA	A0A2B6THR9_9BACI	1-6
36	Bacillus toyonensis	Bacteria	Type VII secretion protein EsaA	A0A2C4DVG4_9BACI	1-6
37	Bacillus toyonensis	Bacteria	Type VII secretion protein EsaA	A0A2B6N743_9BACI	1-6
38	Bacillus toyonensis	Bacteria	Type VII secretion protein EsaA	A0A2B5DH91_9BACI	1-6
39	Bacillus toyonensis	Bacteria	Type VII secretion protein EsaA	A0A2C4FE08_9BACI	1-6
40	Bacillus toyonensis	Bacteria	Type VII secretion protein EsaA	A0A2C4V6Q4_9BACI	1-6
41	Bacillus toyonensis	Bacteria	Type VII secretion protein EsaA	A0A2B6YUR3_9BACI	1-6
42	Bacillus toyonensis	Bacteria	Type VII secretion protein EsaA	A0A2C4RJ52_9BACI	1-6
43	Bacillus toyonensis	Bacteria	Type VII secretion protein EsaA	A0A2A8AEX4_9BACI	1-6
44	Bacillus toyonensis	Bacteria	Type VII secretion protein EsaA	A0A2B6T4C0_9BACI	1-6
45	Bacillus toyonensis	Bacteria	Type VII secretion protein EsaA	A0A2B5U8E7_9BACI	1-6
46	Bacillus toyonensis	Bacteria	Type VII secretion protein EsaA	A0A2B7E2X1_9BACI	1-6
47	Bacillus toyonensis	Bacteria	Type VII secretion protein EsaA	A0A2B4X280_9BACI	1-6

48	Bacillus toyonensis	Bacteria	Type VII secretion protein EsaA	A0A2C5E0V9_9BACI	1-6
49	Bacillus toyonensis	Bacteria	Type VII secretion protein EsaA	A0A2B5Z5V1_9BACI	1-6
50	Bacillus toyonensis	Bacteria	Type VII secretion protein EsaA	A0A1X3MTJ3_9BACI	1-6
51	Bacillus toyonensis	Bacteria	Type VII secretion protein EsaA	A0A2A8HG02_9BACI	1-6
52	Bacteroides dorei	Bacteria	Membrane protein	A0A0K2HPY7_9BACE	623-629
53	Bacteroides dorei	Bacteria	Membrane protein	A0A076IJA6_9BACE	603-609
54	Bacteroides dorei	Bacteria	SusC/RagA family TonB-linked outer membrane protein	A0A1Y4PF17_9BACE	631-607
55	Bacteroides dorei CL02T12C06	Bacteria	SusC/RagA family TonB-linked outer membrane protein	I8WC85_9BACE	631-607
56	Bacteroides uniformis	Bacteria	SusC/RagA family TonB-linked outer membrane protein	A0A1Q6I253_BACUN	593-599
57	Bacteroides vulgatus	Bacteria	Outer membrane receptor proteins, mostly iron transport	A0A174L978_BACVU	631-637
58	Bacteroidetes bacterium OLB8	Bacteria	DMT(Drug/metabolite transporter) superfamily permease	A0A136LD73_9BACT	856-862
59	Bellilinea caldifistulae	Bacteria	Uncharacterized protein	A0A0P6XBM6_9CHLR	1-6
60	Brevefilum fermentans	Bacteria	Putative ABC transporter substrate binding protein	A0A1Y6K6Q8_9CHLR	1-6
61	Chloroflexi bacterium GWB2_54_36	Bacteria	Uncharacterized protein	A0A1F8KKN9_9CHLR	1-6
62	Citrobacter amalonaticus Y19	Bacteria	PhoP/PhoQ regulator MgrB	M1KGH3_CITAM	1-6
63	Citrobacter farmeri	Bacteria	PhoP/PhoQ regulator MgrB	A0A223JSW8_9ENTR	1-6
64	Citrobacter koseri (Citrobacter diversus)	Bacteria	PhoP/PhoQ regulator MgrB	A0A1Z3Y021_CITKO	1-6
65	Citrobacter koseri (strain ATCC BAA-895 / CDC 4225-83 / SGSC4696)	Bacteria	PhoP/PhoQ regulator MgrB	MGRB_CITK8	1-6
66	Citrobacter pasteurii	Bacteria	PhoP/PhoQ regulator MgrB	A0A0A1RC41_9ENTR	1-6
67	Citrobacter rodentium (strain ICC168) (Citrobacter freundii biotype 4280)	Bacteria	Putative exported protein	D2TM43_CITRI	1-6
68	Clostridia bacterium 41_269	Bacteria	Extracellular solute-binding protein family 3	A0A124FD47_9FIRM	1-6
69	Corynebacterium jeikeium	Bacteria	PhoP regulon feedback inhibition membrane protein MgrB	A0A1V3DM18_CORJE	1-6
70	Cronobacter sakazakii (Enterobacter sakazakii)	Bacteria	PhoP/PhoQ regulator MgrB	A0A0F6VUC5_CROSK	1-6

71	Cronobacter sakazakii (strain ATCC BAA-894) (Enterobacter sakazakii)	Bacteria	PhoP/PhoQ regulator MgrB	MGRB_CROS8	1-6
72	Cronobacter sakazakii 696	Bacteria	PhoP/PhoQ regulator MgrB	K8D9J2_CROSK	1-6
73	Cronobacter sakazakii 701	Bacteria	PhoP/PhoQ regulator MgrB	K8CDK0_CROSK	1-6
74	Cronobacter turicensis (strain DSM 18703 / LMG 23827 / z3032)	Bacteria	PhoP/PhoQ regulator MgrB	C9XUH3_CROTZ	1-6
75	Cronobacter turicensis 564	Bacteria	PhoP/PhoQ regulator MgrB	K8B7R0_9ENTR	1-6
76	Cronobacter universalis NCTC 9529	Bacteria	PhoP/PhoQ regulator MgrB	K8DBV6_9ENTR	1-6
77	Desulfotomaculum guttoideum	Bacteria	PTS system IIA, IIB, and IIC component, Fru family	A0A1I0KKL6_9FIRM	408-414
78	Enterobacteriaceae bacterium (strain FGI 57)	Bacteria	PhoP/PhoQ regulator MgrB	L0M508_ENTBF	1-6
79	Enterococcus canintestini	Bacteria	Spermidine/putrescine ABC transporter permease	A0A267HR32_9ENTE	1-6
80	Enterococcus casseliflavus (Enterococcus flavescens)	Bacteria	Spermidine/putrescine ABC transporter permease	A0A1I1UIB9_ENTCA	1-6
81	Enterococcus casseliflavus (strain EC10)	Bacteria	Binding-protein-dependent transport system inner membrane component	C9CNC9_ENTCS	1-6
82	Enterococcus casseliflavus 14-MB-W-14	Bacteria	Putative spermidine/putrescine ABC transporter membrane protein	S4BFW8_ENTCA	1-6
83	Enterococcus casseliflavus ATCC 12755	Bacteria	ABC transporter, permease protein	F0EIX0_ENTCA	1-6
84	Enterococcus casseliflavus EC20	Bacteria	Binding-protein-dependent transport system inner membrane component	C9AC16_ENTCA	1-6
85	Enterococcus dispar ATCC 51266	Bacteria	Spermidine/putrescine ABC transporter permease	S1P035_9ENTE	1-6
86	Enterococcus faecalis 06-MB-DW-09	Bacteria	Putative spermidine/putrescine ABC transporter membrane protein	S4CIJ1_ENTFL	1-6
87	Enterococcus faecium (Streptococcus faecium)	Bacteria	Putative spermidine/putrescine ABC transporter membrane protein	A0A242IZ97_ENTFC	1-6
88	Enterococcus faecium 13.SD.W.09	Bacteria	Putative spermidine/putrescine ABC transporter membrane protein	T2NQ62_ENTFC	1-6
89	Enterococcus flavescens ATCC 49996	Bacteria	Putative spermidine/putrescine ABC transporter membrane protein	R2QYD9_ENTCA	1-6
90	Enterococcus gallinarum	Bacteria	Putative spermidine/putrescine ABC transporter membrane protein	A0A120LL15_ENTGA	1-6
91	Enterococcus gallinarum	Bacteria	Putative spermidine/putrescine ABC transporter membrane protein	A0A1V8Z5M1_ENTGA	1-6
92	Enterococcus gallinarum (strain EG2)	Bacteria	Binding-protein-dependent transport system inner membrane component	C9A0I7_ENTGE	1-6

93	Enterococcus saccharolyticus 30_1	Bacteria	Uncharacterized protein	G5ITB3_9ENTE	1-6
94	Enterococcus sp. 2F9_DIV0599	Bacteria	Spermidine/putrescine ABC transporter permease PotC	A0A242EEX8_9ENTE	1-6
95	Enterococcus sp. 2G9_DIV0600	Bacteria	Spermidine/putrescine ABC transporter permease PotC	A0A2C9XI44_9ENTE	1-6
96	Enterococcus sp. 3C7_DIV0644	Bacteria	Spermidine/putrescine ABC transporter permease PotC	A0A242DSB7_9ENTE	1-6
97	Enterococcus sp. 3C8_DIV0646	Bacteria	Spermidine/putrescine ABC transporter permease PotC	A0A242E4S7_9ENTE	1-6
98	Enterococcus sp. 3G6_DIV0642	Bacteria	Spermidine/putrescine ABC transporter permease PotC	A0A242D6E8_9ENTE	1-6
99	Enterococcus sp. 4E1_DIV0656	Bacteria	Spermidine/putrescine ABC transporter permease PotC	A0A242CNK0_9ENTE	1-6
100	Enterococcus sp. 5B3_DIV0040	Bacteria	Spermidine/putrescine ABC transporter permease PotC	A0A242BZZ1_9ENTE	1-6
101	Enterococcus sp. 6D12_DIV0197	Bacteria	Spermidine/putrescine ABC transporter permease PotC	A0A200IJG8_9ENTE	1-6
102	Enterococcus sp. 8E11_MSG4843	Bacteria	Spermidine/putrescine ABC transporter permease PotC	A0A200JCD6_9ENTE	1-6
103	Enterococcus sp. C1	Bacteria	Spermidine/putrescine ABC transporter permease PotC	J1HTL6_9ENTE	1-6
104	Escherichia albertii	Bacteria	PhoP/PhoQ regulator MgrB	A0A0S3NKU5_9ESCH	1-6
105	Escherichia albertii (strain TW07627)	Bacteria	PhoP/PhoQ regulator MgrB	B1EQ07_ESCAT	1-6
106	Escherichia coli	Bacteria	PhoP regulon feedback inhibition membrane protein MgrB	A0A2B7M6E9_ECOLX	1-6
107	Escherichia coli	Bacteria	PhoP regulon feedback inhibition membrane protein MgrB	A0A2B7PKL6_ECOLX	1-6
108	Escherichia coli	Bacteria	PhoP/PhoQ regulator MgrB	A0A0B1MU87_ECOLX	1-6
109	Escherichia coli	Bacteria	PhoP/PhoQ regulator MgrB	C3T687_ECOLX	1-6
110	Escherichia coli	Bacteria	PhoP/PhoQ regulator MgrB	E2QN28_ECOLX	1-6
111	Escherichia coli	Bacteria	PhoP/PhoQ regulator MgrB	A0A245NPM2_ECOLX	1-6
112	Escherichia coli	Bacteria	PhoP/PhoQ regulator MgrB	C3T690_ECOLX	1-6
113	Escherichia coli	Bacteria	PhoP/PhoQ regulator MgrB	A0A166SZZ7_ECOLX	1-6
114	Escherichia coli (strain 55989 / EAEC)	Bacteria	PhoP/PhoQ regulator MgrB	MGRB_ECO55	1-6
115	Escherichia coli (strain ATCC 8739 / DSM 1576 / Crooks)	Bacteria	PhoP/PhoQ regulator MgrB	MGRB_ECOLC	1-6
116	Escherichia coli (strain ATCC 9637 / CCM 2024 / DSM 1116 / NCIMB 8666 / NRRL B-766 / W)	Bacteria	PhoP/PhoQ regulator MgrB	E8PVX6_ECOLW	1-6

117	Escherichia coli (strain B / BL21-DE3)	Bacteria	PhoP/PhoQ regulator MgrB	A0A140N8W7_ECOBD	1-6
118	Escherichia coli (strain K12 / DH10B)	Bacteria	PhoP/PhoQ regulator MgrB	MGRB_ECODH	1-6
119	Escherichia coli (strain K12 / MC4100 / BW2952)	Bacteria	PhoP/PhoQ regulator MgrB	MGRB_ECOBW	1-6
120	Escherichia coli (strain K12)	Bacteria	PhoP/PhoQ regulator MgrB	MGRB_ECOLI	1-6
121	Escherichia coli (strain SE11)	Bacteria	PhoP/PhoQ regulator MgrB	MGRB_ECOSE	1-6
122	Escherichia coli (strain SMS-3-5 / SECEC)	Bacteria	PhoP/PhoQ regulator MgrB	MGRB_ECOSM	1-6
123	Escherichia coli (strain UTI89 / UPEC)	Bacteria	PhoP/PhoQ regulator MgrB	MGRB_ECOUT	1-6
124	Escherichia coli 1.2264	Bacteria	PhoP/PhoQ regulator MgrB	I2SPL6_ECOLX	1-6
125	Escherichia coli 1.2741	Bacteria	PhoP/PhoQ regulator MgrB	I2RJX3_ECOLX	1-6
126	Escherichia coli 110957	Bacteria	PhoP/PhoQ regulator MgrB	U9YSH3_ECOLX	1-6
127	Escherichia coli 1-110-08_S3_C1	Bacteria	PhoP/PhoQ regulator MgrB	A0A125X2S3_ECOLX	1-6
128	Escherichia coli 113290	Bacteria	PhoP/PhoQ regulator MgrB	U9Z5T2_ECOLX	1-6
129	Escherichia coli 113303	Bacteria	PhoP/PhoQ regulator MgrB	U9XWI6_ECOLX	1-6
130	Escherichia coli 1-176-05_S3_C2	Bacteria	PhoP/PhoQ regulator MgrB	A0A017III5_ECOLX	1-6
131	Escherichia coli 1-250-04_S3_C2	Bacteria	PhoP/PhoQ regulator MgrB	A0A080J2Z5_ECOLX	1-6
132	Escherichia coli 1303	Bacteria	PhoP/PhoQ regulator MgrB	A0A0E1M053_ECOLX	1-6
133	Escherichia coli 1-392-07_S4_C1	Bacteria	PhoP/PhoQ regulator MgrB	A0A074HXH8_ECOLX	1-6
134	Escherichia coli 1-392-07_S4_C3	Bacteria	PhoP/PhoQ regulator MgrB	A0A080FSW3_ECOLX	1-6
135	Escherichia coli 2.3916	Bacteria	PhoP/PhoQ regulator MgrB	I2XER0_ECOLX	1-6
136	Escherichia coli 2-005-03_S4_C2	Bacteria	PhoP/PhoQ regulator MgrB	A0A029IT43_ECOLX	1-6
137	Escherichia coli 2-005-03_S4_C3	Bacteria	PhoP/PhoQ regulator MgrB	A0A029HZ23_ECOLX	1-6
138	Escherichia coli 2-011-08_S1_C1	Bacteria	PhoP/PhoQ regulator MgrB	A0A062Y005_ECOLX	1-6
139	Escherichia coli 2-177-06_S3_C2	Bacteria	PhoP/PhoQ regulator MgrB	A0A070V2T7_ECOLX	1-6
140	Escherichia coli 2-210-07_S3_C3	Bacteria	PhoP/PhoQ regulator MgrB	A0A070SW86_ECOLX	1-6
141	Escherichia coli 2362-75	Bacteria	PhoP/PhoQ regulator MgrB	E3XP38_ECOLX	1-6

142	Escherichia coli 2-427-07_S4_C3	Bacteria	PhoP/PhoQ regulator MgrB	A0A073FWP5_ECOLX	1-6
143	Escherichia coli 3-267-03_S4_C1	Bacteria	PhoP/PhoQ regulator MgrB	A0A073GBN8_ECOLX	1-6
144	Escherichia coli 3-373-03_S4_C2	Bacteria	PhoP/PhoQ regulator MgrB	A0A069XI43_ECOLX	1-6
145	Escherichia coli 4.0522	Bacteria	PhoP/PhoQ regulator MgrB	I2UJ85_ECOLX	1-6
146	Escherichia coli 4.0967	Bacteria	PhoP/PhoQ regulator MgrB	I2WU26_ECOLX	1-6
147	Escherichia coli 53638	Bacteria	PhoP/PhoQ regulator MgrB	A0A0E1T0C7_ECOLX	1-6
148	Escherichia coli 5-366-08_S1_C1	Bacteria	PhoP/PhoQ regulator MgrB	A0A073USV5_ECOLX	1-6
149	Escherichia coli 5-366-08_S1_C3	Bacteria	PhoP/PhoQ regulator MgrB	A0A073HHE3_ECOLX	1-6
150	Escherichia coli 541-15	Bacteria	PhoP/PhoQ regulator MgrB	I4SWD7_ECOLX	1-6
151	Escherichia coli 6-537-08_S1_C3	Bacteria	PhoP/PhoQ regulator MgrB	A0A080FEG9_ECOLX	1-6
152	Escherichia coli 9.0111	Bacteria	PhoP/PhoQ regulator MgrB	I2W471_ECOLX	1-6
153	Escherichia coli 907672	Bacteria	PhoP/PhoQ regulator MgrB	V0S9T0_ECOLX	1-6
154	Escherichia coli 907713	Bacteria	PhoP/PhoQ regulator MgrB	U9Z453_ECOLX	1-6
155	Escherichia coli 908519	Bacteria	PhoP/PhoQ regulator MgrB	V0VLI9_ECOLX	1-6
156	Escherichia coli 908525	Bacteria	PhoP/PhoQ regulator MgrB	V0Y445_ECOLX	1-6
157	Escherichia coli 908573	Bacteria	PhoP/PhoQ regulator MgrB	V0ZF78_ECOLX	1-6
158	Escherichia coli 909945-2	Bacteria	PhoP/PhoQ regulator MgrB	U9ZGG3_ECOLX	1-6
159	Escherichia coli 97.0246	Bacteria	PhoP/PhoQ regulator MgrB	I2S468_ECOLX	1-6
160	Escherichia coli 99.0741	Bacteria	PhoP/PhoQ regulator MgrB	V6FR77_ECOLX	1-6
161	Escherichia coli ATCC BAA-2209	Bacteria	PhoP/PhoQ regulator MgrB	V8FM95_ECOLX	1-6
162	Escherichia coli B354	Bacteria	PhoP/PhoQ regulator MgrB	D6JAA5_ECOLX	1-6
163	Escherichia coli chi7122	Bacteria	PhoP/PhoQ regulator MgrB	J7QM34_ECOLX	1-6
164	Escherichia coli D6-113.11	Bacteria	PhoP/PhoQ regulator MgrB	A0A024KXF8_ECOLX	1-6
165	Escherichia coli DEC1B	Bacteria	PhoP/PhoQ regulator MgrB	H4IBV5_ECOLX	1-6
166	Escherichia coli DEC1C	Bacteria	PhoP/PhoQ regulator MgrB	H4ISR4_ECOLX	1-6

167	Escherichia coli DEC1D	Bacteria	PhoP/PhoQ regulator MgrB	H4J911_ECOLX	1-6
168	Escherichia coli DEC2C	Bacteria	PhoP/PhoQ regulator MgrB	H4KHU7_ECOLX	1-6
169	Escherichia coli DEC2E	Bacteria	PhoP/PhoQ regulator MgrB	H4LCR2_ECOLX	1-6
170	Escherichia coli DEC6A	Bacteria	PhoP/PhoQ regulator MgrB	H4UI03_ECOLX	1-6
171	Escherichia coli E1114	Bacteria	PhoP/PhoQ regulator MgrB	A0A1X3IN00_ECOLX	1-6
172	Escherichia coli E1118	Bacteria	PhoP/PhoQ regulator MgrB	A0A1X3MBX5_ECOLX	1-6
173	Escherichia coli G3/10	Bacteria	PhoP/PhoQ regulator MgrB	A0A0A0FHQ3_ECOLX	1-6
174	Escherichia coli H386	Bacteria	PhoP/PhoQ regulator MgrB	A0A1X3JGR7_ECOLX	1-6
175	Escherichia coli H461	Bacteria	PhoP/PhoQ regulator MgrB	A0A1X3KBM9_ECOLX	1-6
176	Escherichia coli H591	Bacteria	PhoP/PhoQ regulator MgrB	F4VG73_ECOLX	1-6
177	Escherichia coli H605	Bacteria	PhoP/PhoQ regulator MgrB	A0A1X3KRT6_ECOLX	1-6
178	Escherichia coli H736	Bacteria	PhoP/PhoQ regulator MgrB	F4SKX9_ECOLX	1-6
179	Escherichia coli ISC7	Bacteria	PhoP/PhoQ regulator MgrB	W1EVS7_ECOLX	1-6
180	Escherichia coli LAU-EC10	Bacteria	PhoP/PhoQ regulator MgrB	V8KE55_ECOLX	1-6
181	Escherichia coli M056	Bacteria	PhoP/PhoQ regulator MgrB	A0A1X3I7C9_ECOLX	1-6
182	Escherichia coli M605	Bacteria	PhoP/PhoQ regulator MgrB	F4SZ56_ECOLX	1-6
183	Escherichia coli M718	Bacteria	PhoP/PhoQ regulator MgrB	F4TFF4_ECOLX	1-6
184	Escherichia coli M8	Bacteria	PhoP/PhoQ regulator MgrB	A0A1S6TBH1_ECOLX	1-6
185	Escherichia coli M863	Bacteria	PhoP/PhoQ regulator MgrB	E9YP17_ECOLX	1-6
186	Escherichia coli MP021561.2	Bacteria	PhoP/PhoQ regulator MgrB	M9H086_ECOLX	1-6
187	Escherichia coli MS 119-7	Bacteria	PhoP/PhoQ regulator MgrB	D8EC48_ECOLX	1-6
188	Escherichia coli MS 124-1	Bacteria	PhoP/PhoQ regulator MgrB	E1JC52_ECOLX	1-6
189	Escherichia coli MS 145-7	Bacteria	PhoP/PhoQ regulator MgrB	E1IML7_ECOLX	1-6
190	Escherichia coli MS 85-1	Bacteria	PhoP/PhoQ regulator MgrB	E6BEP1_ECOLX	1-6
191	Escherichia coli O1:K1 / APEC	Bacteria	PhoP/PhoQ regulator MgrB	MGRB_ECOK1	1-6

192	Escherichia coli O103:H2 (strain 12009 / EHEC)	Bacteria	PhoP/PhoQ regulator MgrB	C8UA84_ECO10	1-6
193	Escherichia coli O104:H4 (strain 2011C-3493)	Bacteria	PhoP/PhoQ regulator MgrB	A0A0E0XY13_ECO1C	1-6
194	Escherichia coli O111:H- (strain 11128 / EHEC)	Bacteria	PhoP/PhoQ regulator MgrB	C8UBS5_ECO1A	1-6
195	Escherichia coli O111:H11 str. CVM9455	Bacteria	PhoP/PhoQ regulator MgrB	K4WG83_ECOLX	1-6
196	Escherichia coli O111:H8 str. CVM9634	Bacteria	PhoP/PhoQ regulator MgrB	K4V6M8_ECOLX	1-6
197	Escherichia coli O111:NM str. K6722	Bacteria	PhoP/PhoQ regulator MgrB	A0A027TT17_ECOLX	1-6
198	Escherichia coli O118:H16 str. 2009C-4446	Bacteria	PhoP/PhoQ regulator MgrB	A0A028E2J5_ECOLX	1-6
199	Escherichia coli O121:H19 str. 2010C-3609	Bacteria	PhoP/PhoQ regulator MgrB	A0A0E2TSV4_ECOLX	1-6
200	Escherichia coli O127:H6	Bacteria	Uncharacterized protein	A0A2D0P6H6_ECOLX	1-6
201	Escherichia coli O127:H6 (strain E2348/69 / EPEC)	Bacteria	PhoP/PhoQ regulator MgrB	MGRB_ECO27	1-6
202	Escherichia coli O128:H2 str. 2011C-3317	Bacteria	PhoP/PhoQ regulator MgrB	A0A070ETA6_ECOLX	1-6
203	Escherichia coli O139:H28 (strain E24377A / ETEC)	Bacteria	PhoP/PhoQ regulator MgrB	MGRB_ECO24	1-6
204	Escherichia coli O145:H25 str. 07-3858	Bacteria	PhoP/PhoQ regulator MgrB	A0A070D363_ECOLX	1-6
205	Escherichia coli O145:H28 str. RM12581	Bacteria	PhoP/PhoQ regulator MgrB	A0A023YXY9_ECOLX	1-6
206	Escherichia coli O145:NM str. 2010C-3526	Bacteria	PhoP/PhoQ regulator MgrB	A0A025CNG5_ECOLX	1-6
207	Escherichia coli O146:H21 str. 2010C-3325	Bacteria	PhoP/PhoQ regulator MgrB	A0A070CRX4_ECOLX	1-6
208	Escherichia coli O157	Bacteria	PhoP/PhoQ regulator MgrB	A0A1Z3UR72_ECOLX	1-6
209	Escherichia coli O157:H7	Bacteria	PhoP/PhoQ regulator MgrB	MGRB_ECO57	1-6
210	Escherichia coli O157:H7 (strain EC4115 / EHEC)	Bacteria	PhoP/PhoQ regulator MgrB	MGRB_ECO5E	1-6
211	Escherichia coli O157:H7 str. SS52	Bacteria	PhoP/PhoQ regulator MgrB	A0A0F6F7I3_ECO57	1-6
212	Escherichia coli O17:K52:H18 (strain UMN026 / ExPEC)	Bacteria	PhoP/PhoQ regulator MgrB	MGRB_ECOLU	1-6
213	Escherichia coli O174:H8 str. 04-3038	Bacteria	PhoP/PhoQ regulator MgrB	A0A026V3K9_ECOLX	1-6
214	Escherichia coli O25b:H4	Bacteria	PhoP/PhoQ regulator MgrB	A0A192C884_ECOLX	1-6

215	Escherichia coli O25b:H4-ST131	Bacteria	PhoP/PhoQ regulator MgrB	W8ZJQ3_ECOLX	1-6
216	Escherichia coli O45:K1 (strain S88 / ExPEC)	Bacteria	PhoP/PhoQ regulator MgrB	MGRB_ECO45	1-6
217	Escherichia coli O55:H7 (strain CB9615 / EPEC)	Bacteria	PhoP/PhoQ regulator MgrB	D3QVU2_ECOCB	1-6
218	Escherichia coli O6:H1 (strain CFT073 / ATCC 700928 / UPEC)	Bacteria	PhoP/PhoQ regulator MgrB	MGRB_ECOL6	1-6
219	Escherichia coli O6:K15:H31 (strain 536 / UPEC)	Bacteria	PhoP/PhoQ regulator MgrB	MGRB_ECOL5	1-6
220	Escherichia coli O69:H11 str. 08-4661	Bacteria	PhoP/PhoQ regulator MgrB	A0A027ZLA9_ECOLX	1-6
221	Escherichia coli O7:K1 str. CE10	Bacteria	PhoP/PhoQ regulator MgrB	A0A0E0V651_ECOLX	1-6
222	Escherichia coli O78:H11 (strain H10407 / ETEC)	Bacteria	PhoP/PhoQ regulator MgrB	E3PNL1_ECOH1	1-6
223	Escherichia coli O8 (strain IAI1)	Bacteria	PhoP/PhoQ regulator MgrB	MGRB_ECO8A	1-6
224	Escherichia coli O81 (strain ED1a)	Bacteria	PhoP/PhoQ regulator MgrB	MGRB_ECO81	1-6
225	Escherichia coli O83:H1 (strain NRG 857C / AIEC)	Bacteria	PhoP/PhoQ regulator MgrB	A0A0H3EIC2_ECO8N	1-6
226	Escherichia coli O9:H4 (strain HS)	Bacteria	PhoP/PhoQ regulator MgrB	MGRB_ECOHS	1-6
227	Escherichia coli P0301867.5	Bacteria	PhoP/PhoQ regulator MgrB	N4P199_ECOLX	1-6
228	Escherichia coli PCN033	Bacteria	PhoP/PhoQ regulator MgrB	A0A0G3K4U9_ECOLX	1-6
229	Escherichia coli TA249	Bacteria	PhoP/PhoQ regulator MgrB	A0A1X3LYF4_ECOLX	1-6
230	Escherichia coli TA280	Bacteria	PhoP/PhoQ regulator MgrB	F4V2X8_ECOLX	1-6
231	Escherichia coli TW10509	Bacteria	PhoP/PhoQ regulator MgrB	E9XJ20_ECOLX	1-6
232	Escherichia coli UMNF18	Bacteria	PhoP/PhoQ regulator MgrB	G0F6U3_ECOLX	1-6
233	Escherichia coli UMNK88	Bacteria	PhoP/PhoQ regulator MgrB	A0A0E0TZ74_ECOLX	1-6
234	Escherichia coli W26	Bacteria	PhoP/PhoQ regulator MgrB	I0VLI4_ECOLX	1-6
235	Escherichia coli Xuzhou21	Bacteria	PhoP/PhoQ regulator MgrB	A0A0F6C5B9_ECOLX	1-6
236	Escherichia fergusonii	Bacteria	PhoP/PhoQ regulator MgrB	A0A0W3CUM4_ESCFE	1-6
237	Escherichia fergusonii (strain ATCC 35469 / DSM 13698 / CDC 0568-73)	Bacteria	PhoP/PhoQ regulator MgrB	MGRB_ESCF3	1-6

238	Franconibacter pulveris	Bacteria	PhoP/PhoQ regulator MgrB	A0A0J8VIN5_9ENTR	1-6
239	Geobacillus galactosidasius	Bacteria	UDP-glucose 6-dehydrogenase	A0A226QKN8_9BACI	1-6
240	Geobacillus sp. (strain WCH70)	Bacteria	Negative regulator of sigma-X activity	C5D3J3_GEOSW	1-6
241	Geobacillus sp. 44B	Bacteria	UDP-glucose 6-dehydrogenase	A0A1V9AY01_9BACI	1-6
242	Geobacillus sp. AYN2	Bacteria	UDP-glucose 6-dehydrogenase	A0A2A6AP47_9BACI	1-6
243	Hymenobacter glacialis	Bacteria	Uncharacterized protein	A0A1G1TBZ3_9BACT	277-284
244	Hymenobacter lapidarius	Bacteria	Uncharacterized protein	A0A1G1TFD6_9BACT	277-284
245	Klebsiella pneumoniae IS22	Bacteria	PhoP/PhoQ regulator MgrB	W1B6K7_KLEPN	1-6
246	Klebsiella sp. RIT-PI-d	Bacteria	PhoPQ regulatory protein	A0A0L0APH5_9ENTR	1-6
247	Kluyvera ascorbata ATCC 33433	Bacteria	PhoP/PhoQ regulator MgrB	A0A085IKE1_9ENTR	1-6
248	Kluyvera georgiana	Bacteria	PhoP/PhoQ regulator MgrB	A0A248KI92_9ENTR	1-6
249	Kluyvera georgiana ATCC 51603	Bacteria	PhoP/PhoQ regulator MgrB	A0A1B7K139_9ENTR	1-6
250	Kluyvera intermedia (Enterobacter intermedius)	Bacteria	PhoP/PhoQ regulator MgrB	A0A1X0XCU6_KLUIN	1-6
251	Lechevalieria fradiae	Bacteria	Cellulose binding domain-containing protein	A0A1G7Z822_9PSEU	1-6
252	Lechevalieria xinjiangensis	Bacteria	Glycosyl hydrolases family 18	A0A1H9QHH0_9PSEU	1-6
253	Lelliottia sp. PFL01	Bacteria	Inner membrane protein	A0A248T0B6_9ENTR	1-6
254	Lentisphaerae bacterium GWF2_44_16	Bacteria	Uncharacterized protein	A0A1G0YQZ4_9BACT	397-403
255	Lentzea albidocapillata	Bacteria	Glycosyl hydrolases family 18	A0A1W2CVS0_9PSEU	1-6
256	Lentzea flaviverrucosa	Bacteria	Glycosyl hydrolases family 18	A0A1H9K1H2_9PSEU	1-6
257	Lentzea violacea	Bacteria	Cellulose binding domain-containing protein	A0A1G9TIW6_9PSEU	1-6
258	Lentzea waywayandensis	Bacteria	Glycosyl hydrolases family 18	A0A1I6FE86_9PSEU	1-6
259	Leptothrix cholodnii (strain ATCC 51168 / LMG 8142 / SP-6) (Leptothrix discophora (strain SP-6))	Bacteria	Uncharacterized protein	B1XX16_LEPCP	1-6
260	Paenibacillus sp. 1_12	Bacteria	Two-component system, sensor histidine kinase YesM	A0A1I4JD84_9BACL	1-6

261	Paenibacillus sp. FSL H7-0331	Bacteria	Uncharacterized protein	A0A1R1C8X1_9BACL	1-6
262	Paenibacillus sp. yr247	Bacteria	Predicted dehydrogenase	A0A1H0C613_9BACL	1-6
263	Parageobacillus caldoxylosilyticus NBRC 107762	Bacteria	Uncharacterized protein	A0A023DIB6_9BACI	1-6
264	Parageobacillus genomosp. 1	Bacteria	Negative regulator of sigma-X activity	A0A023CKI5_9BACI	1-6
265	Parageobacillus toebii	Bacteria	Uncharacterized protein	A0A150N545_9BACI	1-6
266	Photobacterium profundum (strain SS9)	Bacteria	Aspartate/glutamate leucyltransferase	Q6LT11_PHOPR	89-94
267	Photobacterium profundum 3TCK	Bacteria	Aspartate/glutamate leucyltransferase	Q1Z139_9GAMM	89-94
268	Polaromonas sp. 35-63-35	Bacteria	Sodium:proton antiporter	A0A258P8W7_9BURK	1-6
269	Roseburia sp. CAG:380	Bacteria	Putative bacterial extracellular solute-binding protein	R6WNG6_9FIRM	1-6
270	Ruminococcus torques	Bacteria	Ribosomal RNA large subunit methyltransferase Cfr	A0A174XL89_9FIRM	8-14
271	Salmonella agona (strain SL483)	Bacteria	PhoP/PhoQ regulator MgrB	MGRB_SALA4	1-6
272	Salmonella arizonae (strain ATCC BAA-731 / CDC346-86 / RSK2980)	Bacteria	PhoP/PhoQ regulator MgrB	MGRB_SALAR	1-6
273	Salmonella bongori N268-08	Bacteria	PhoP/PhoQ regulator MgrB	S5N950_SALBN	1-6
274	Salmonella bongori serovar 66:z41:- str. SA19983605	Bacteria	PhoP/PhoQ regulator MgrB	A0A248K855_SALBN	1-6
275	Salmonella choleraesuis	Bacteria	PhoP/PhoQ regulator MgrB	A0A0M0PHZ0_SALCE	1-6
276	Salmonella choleraesuis (strain SC-B67)	Bacteria	PhoP/PhoQ regulator MgrB	MGRB_SALCH	1-6
277	Salmonella enterica I	Bacteria	PhoP/PhoQ regulator MgrB	A0A0F7J939_SALET	1-6
278	Salmonella enterica subsp. arizonae serovar 18:z4,z23:- str. CVM N6509	Bacteria	PhoP/PhoQ regulator MgrB	A0A1M3XZH0_SALCE	1-6
279	Salmonella enterica subsp. arizonae serovar 41:z4,z23:-	Bacteria	PhoP/PhoQ regulator MgrB	A0A1X2TN92_SALCE	1-6
280	Salmonella enterica subsp. arizonae serovar 50:r:z	Bacteria	PhoP/PhoQ regulator MgrB	A0A1X2TGL5_SALCE	1-6
281	Salmonella enterica subsp. arizonae serovar 62:z36:- str. RKS2983	Bacteria	PhoP/PhoQ regulator MgrB	A0A089HJH6_SALCE	1-6
282	Salmonella enterica subsp. arizonae serovar 63:g,z51:- str. So 20/20	Bacteria	PhoP/PhoQ regulator MgrB	A0A0V2EBP3_SALCE	1-6

283	Salmonella enterica subsp. diarizonae serovar 50:k:z str. MZ0080	Bacteria	PhoP/PhoQ regulator MgrB	A0A241RCQ3_SALDZ	1-6
284	Salmonella enterica subsp. diarizonae serovar 60:r:e,n,x,z15	Bacteria	PhoP/PhoQ regulator MgrB	A0A232RKS9_SALDZ	1-6
285	Salmonella enterica subsp. diarizonae serovar 65:c:z str. SA20044251	Bacteria	PhoP/PhoQ regulator MgrB	A0A241RZB4_SALDZ	1-6
286	Salmonella enterica subsp. enterica serovar Aberdeen	Bacteria	PhoP regulon feedback inhibition membrane protein MgrB	A0A271GUF2_SALET	1-6
287	Salmonella enterica subsp. enterica serovar Adelaide str. A4-669	Bacteria	PhoP/PhoQ regulator MgrB	G5L875_SALET	1-6
288	Salmonella enterica subsp. enterica serovar Alachua str. R6-377	Bacteria	PhoP/PhoQ regulator MgrB	G5LMX1_SALET	1-6
289	Salmonella enterica subsp. enterica serovar Bareilly	Bacteria	PhoP/PhoQ regulator MgrB	A0A1S1AH73_SALET	1-6
290	Salmonella enterica subsp. enterica serovar Bovismorbificans	Bacteria	PhoP/PhoQ regulator MgrB	A0A0U0WUY1_SALET	1-6
291	Salmonella enterica subsp. enterica serovar Cerro str. CFSAN001590	Bacteria	PhoP/PhoQ regulator MgrB	V7UAQ6_SALET	1-6
292	Salmonella enterica subsp. enterica serovar Cubana str. 76814	Bacteria	PhoP/PhoQ regulator MgrB	V7IPT1_SALET	1-6
293	Salmonella enterica subsp. enterica serovar Cubana str. CFSAN002050	Bacteria	PhoP/PhoQ regulator MgrB	S5HS98_SALET	1-6
294	Salmonella enterica subsp. enterica serovar Gallinarum str. SG9	Bacteria	PhoP/PhoQ regulator MgrB	A0A0G2NLX0_SALGL	1-6
295	Salmonella enterica subsp. enterica serovar Give str. S5-487	Bacteria	PhoP/PhoQ regulator MgrB	G5MHC7_SALET	1-6
296	Salmonella enterica subsp. enterica serovar Heidelberg	Bacteria	PhoP regulon feedback inhibition membrane protein MgrB	A0A265B5M5_SALET	1-6
297	Salmonella enterica subsp. enterica serovar Hvittingfoss str. SA20014981	Bacteria	PhoP/PhoQ regulator MgrB	A0A221YZB0_SALET	1-6
298	Salmonella enterica subsp. enterica serovar India str. SA20085604	Bacteria	PhoP/PhoQ regulator MgrB	A0A1Z3QDI2_SALET	1-6
299	Salmonella enterica subsp. enterica serovar Infantis str. CVM 17958	Bacteria	Transcriptional regulator	A0A2C6FGK2_SALIN	1-6
300	Salmonella enterica subsp. enterica serovar Infantis str. CVM 22577	Bacteria	Transcriptional regulator	A0A2C6HZC3_SALIN	1-6

301	Salmonella enterica subsp. enterica serovar Infantis str. CVM 22582	Bacteria	Transcriptional regulator	A0A2C6I4B5_SALIN	1-6
302	Salmonella enterica subsp. enterica serovar Infantis str. CVM 23697	Bacteria	Transcriptional regulator	A0A2C6HW10_SALIN	1-6
303	Salmonella enterica subsp. enterica serovar Infantis str. CVM 23729	Bacteria	Transcriptional regulator	A0A2C6HE65_SALIN	1-6
304	Salmonella enterica subsp. enterica serovar Infantis str. CVM 818	Bacteria	Transcriptional regulator	A0A2C6H5G5_SALIN	1-6
305	Salmonella enterica subsp. enterica serovar Infantis str. CVM N32590PS	Bacteria	Transcriptional regulator	A0A2C6J8J2_SALIN	1-6
306	Salmonella enterica subsp. enterica serovar Infantis str. CVM N32597PS	Bacteria	Transcriptional regulator	A0A2C6FR64_SALIN	1-6
307	Salmonella enterica subsp. enterica serovar Infantis str. CVM N32599PS	Bacteria	Transcriptional regulator	A0A2C6E8K7_SALIN	1-6
308	Salmonella enterica subsp. enterica serovar Infantis str. CVM N34868PS	Bacteria	Transcriptional regulator	A0A2C6GIB6_SALIN	1-6
309	Salmonella enterica subsp. enterica serovar Infantis str. CVM N35495PS	Bacteria	Transcriptional regulator	A0A2C6GZG5_SALIN	1-6
310	Salmonella enterica subsp. enterica serovar Infantis str. SARB27	Bacteria	PhoP/PhoQ regulator MgrB	G4C2K8_SALIN	1-6
311	Salmonella enterica subsp. enterica serovar Inverness str. R8-3668	Bacteria	PhoP/PhoQ regulator MgrB	G5NBY2_SALET	1-6
312	Salmonella enterica subsp. enterica serovar Johannesburg	Bacteria	PhoP/PhoQ regulator MgrB	A0A0L9F773_SALET	1-6
313	Salmonella enterica subsp. enterica serovar Kentucky	Bacteria	PhoP regulon feedback inhibition membrane protein MgrB	A0A271CSV7_SALET	1-6
314	Salmonella enterica subsp. enterica serovar Kentucky str. SA20030505	Bacteria	PhoP/PhoQ regulator MgrB	A0A221YLJ6_SALET	1-6
315	Salmonella enterica subsp. enterica serovar Macclesfield str. S-1643	Bacteria	PhoP regulon feedback inhibition membrane protein MgrB	A0A2C9NZ28_SALET	1-6
316	Salmonella enterica subsp. enterica serovar Mississippi str. A4-633	Bacteria	PhoP/PhoQ regulator MgrB	G5PM42_SALET	1-6
317	Salmonella enterica subsp. enterica serovar Montevideo str. 609458-2	Bacteria	PhoP/PhoQ regulator MgrB	A0A1C3A297_SALMO	1-6

318	Salmonella enterica subsp. enterica serovar Montevideo str. S5-403	Bacteria	PhoP/PhoQ regulator MgrB	G5Q1K9_SALMO	1-6
319	Salmonella enterica subsp. enterica serovar Muenchen str. baa1594	Bacteria	PhoP/PhoQ regulator MgrB	V1VZ47_SALMU	1-6
320	Salmonella enterica subsp. enterica serovar Rough O:d:1,7	Bacteria	PhoP/PhoQ regulator MgrB	A0A1X2RFL4_SALET	1-6
321	Salmonella enterica subsp. enterica serovar Rubislaw str. A4-653	Bacteria	PhoP/PhoQ regulator MgrB	G5QI15_SALRU	1-6
322	Salmonella enterica subsp. enterica serovar Saintpaul	Bacteria	PhoP/PhoQ regulator MgrB	A0A1S0ZF23_SALET	1-6
323	Salmonella enterica subsp. enterica serovar Saphra	Bacteria	PhoP/PhoQ regulator MgrB	A0A1X2YCS1_SALET	1-6
324	Salmonella enterica subsp. enterica serovar Schwarzengrund	Bacteria	PhoP regulon feedback inhibition membrane protein MgrB	A0A271CJU2_SALET	1-6
325	Salmonella enterica subsp. enterica serovar Seftenburg	Bacteria	PhoP/PhoQ regulator MgrB	A0A1X2UNT4_SALET	1-6
326	Salmonella enterica subsp. enterica serovar Senftenberg str. A4-543	Bacteria	PhoP/PhoQ regulator MgrB	G5QYU8_SALSE	1-6
327	Salmonella enterica subsp. enterica serovar Tennessee str. TXSC_TXSC08-19	Bacteria	PhoP/PhoQ regulator MgrB	X2KJG1_SALET	1-6
328	Salmonella enterica subsp. enterica serovar Typhimurium str. DT104	Bacteria	PhoP/PhoQ regulator MgrB	A0A0U1G4T3_SALTM	1-6
329	Salmonella enterica subsp. enterica serovar Uganda str. R8-3404	Bacteria	PhoP/PhoQ regulator MgrB	G5REM6_SALET	1-6
330	Salmonella enterica subsp. enterica serovar Urbana str. R8-2977	Bacteria	PhoP/PhoQ regulator MgrB	G5RUD1_SALET	1-6
331	Salmonella enterica subsp. enterica serovar Wandsworth str. A4-580	Bacteria	PhoP/PhoQ regulator MgrB	G5SAS6_SALET	1-6
332	Salmonella enterica subsp. indica serovar 6,14,25:z10:1,(2),7 str. 1121	Bacteria	PhoP/PhoQ regulator MgrB	V1GR58_SALCE	1-6
333	Salmonella enterica subsp. salamae	Bacteria	PhoP/PhoQ regulator MgrB	A0A0F5BER6_SALCE	1-6
334	Salmonella enterica subsp. salamae serovar 55:k:z39 str. 1315K	Bacteria	PhoP/PhoQ regulator MgrB	A0A241SEH7_SALCE	1-6
335	Salmonella enterica subsp. salamae serovar 56:z10:e,n,x str. 1369-73	Bacteria	PhoP/PhoQ regulator MgrB	A0A0V2CSS7_SALCE	1-6

336	Salmonella enteritidis	Bacteria	PhoP/PhoQ regulator MgrB	A0A1R2TMC4_SALEN	1-6
337	Salmonella enteritidis PT4 (strain P125109)	Bacteria	PhoP/PhoQ regulator MgrB	MGRB_SALEP	1-6
338	Salmonella gallinarum (strain 287/91 / NCTC 13346)	Bacteria	PhoP/PhoQ regulator MgrB	MGRB_SALG2	1-6
339	Salmonella heidelberg (strain SL476)	Bacteria	PhoP/PhoQ regulator MgrB	MGRB_SALHS	1-6
340	Salmonella houtenae	Bacteria	PhoP/PhoQ regulator MgrB	A0A1J7KM92_SALHO	1-6
341	Salmonella infantis	Bacteria	PhoP regulon feedback inhibition membrane protein MgrB	A0A267NXT3_SALIN	1-6
342	Salmonella newport	Bacteria	PhoP/PhoQ regulator MgrB	A0A0R9ND34_SALNE	1-6
343	Salmonella newport (strain SL254)	Bacteria	PhoP/PhoQ regulator MgrB	MGRB_SALNS	1-6
344	Salmonella paratyphi A (strain AKU_12601)	Bacteria	PhoP/PhoQ regulator MgrB	MGRB_SALPK	1-6
345	Salmonella paratyphi A (strain ATCC 9150 / SARB42)	Bacteria	PhoP/PhoQ regulator MgrB	MGRB_SALPA	1-6
346	Salmonella paratyphi B (strain ATCC BAA-1250 / SPB7)	Bacteria	PhoP/PhoQ regulator MgrB	MGRB_SALPB	1-6
347	Salmonella paratyphi C (strain RKS4594)	Bacteria	PhoP/PhoQ regulator MgrB	MGRB_SALPC	1-6
348	Salmonella schwarzengrund (strain CVM19633)	Bacteria	PhoP/PhoQ regulator MgrB	MGRB_SALSV	1-6
349	Salmonella senftenberg	Bacteria	PhoP regulon feedback inhibition membrane protein MgrB	A0A267SIQ5_SALSE	1-6
350	Salmonella sp. HMSC13B08	Bacteria	PhoP/PhoQ regulator MgrB	A0A1F2JBN1_9ENTR	1-6
351	Salmonella typhi	Bacteria	PhoP/PhoQ regulator MgrB	MGRB_SALTI	1-6
352	Salmonella typhimurium	Bacteria	PhoP/PhoQ regulator MgrB	A0A0J0VVZ8_SALTM	1-6
353	Salmonella typhimurium (strain 14028s / SGSC 2262)	Bacteria	PhoP/PhoQ regulator MgrB	MGRB_SALT1	1-6
354	Salmonella typhimurium (strain 4/74)	Bacteria	PhoP/PhoQ regulator MgrB	E8X999_SALT4	1-6
355	Salmonella typhimurium (strain LT2 / SGSC1412 / ATCC 700720)	Bacteria	PhoP/PhoQ regulator MgrB	MGRB_SALTY	1-6
356	Salmonella typhimurium (strain SL1344)	Bacteria	PhoP/PhoQ regulator MgrB	A0A0H3NDR4_SALTS	1-6
357	Sebaldella termitidis (strain ATCC 33386 / NCTC 11300)	Bacteria	Galactose-binding protein	D1AIH6_SEBTE	1-6
358	Shigella boydii	Bacteria	PhoP/PhoQ regulator MgrB	A0A1Q8MEG0_SHIBO	1-6

359	Shigella boydii 4444-74	Bacteria	PhoP/PhoQ regulator MgrB	I6E5W5_SHIBO	1-6
360	Shigella boydii 5216-82	Bacteria	PhoP/PhoQ regulator MgrB	F3WJ64_9ENTR	1-6
361	Shigella boydii 965-58	Bacteria	PhoP/PhoQ regulator MgrB	I6DGX5_SHIBO	1-6
362	Shigella boydii ATCC 9905	Bacteria	PhoP/PhoQ regulator MgrB	E7SVL3_9ENTR	1-6
363	Shigella boydii serotype 18 (strain CDC 3083-94 / BS512)	Bacteria	PhoP/PhoQ regulator MgrB	MGRB_SHIB3	1-6
364	Shigella boydii serotype 4 (strain Sb227)	Bacteria	PhoP/PhoQ regulator MgrB	MGRB_SHIBS	1-6
365	Shigella dysenteriae	Bacteria	PhoP/PhoQ regulator MgrB	A0A1Q8NRP2_SHIDY	1-6
366	Shigella dysenteriae 1012	Bacteria	PhoP/PhoQ regulator MgrB	B3X699_9ENTR	1-6
367	Shigella dysenteriae 1617	Bacteria	PhoP/PhoQ regulator MgrB	E2XFQ9_SHIDY	1-6
368	Shigella dysenteriae 225-75	Bacteria	PhoP/PhoQ regulator MgrB	I6FV90_SHIDY	1-6
369	Shigella dysenteriae CDC 74-1112	Bacteria	PhoP/PhoQ regulator MgrB	E7SEJ5_SHIDY	1-6
370	Shigella dysenteriae serotype 1 (strain Sd197)	Bacteria	PhoP/PhoQ regulator MgrB	MGRB_SHIDS	1-6
371	Shigella dysenteriae WRSd3	Bacteria	PhoP/PhoQ regulator MgrB	A0A090NX03_SHIDY	1-6
372	Shigella flexneri	Bacteria	PhoP/PhoQ regulator MgrB	MGRB_SHIFL	1-6
373	Shigella flexneri	Bacteria	PhoP/PhoQ regulator MgrB	A0A1W2MJ79_SHIFL	1-6
374	Shigella flexneri 1235-66	Bacteria	PhoP/PhoQ regulator MgrB	I6H4S5_SHIFL	1-6
375	Shigella flexneri 1485-80	Bacteria	PhoP/PhoQ regulator MgrB	K0X9Q9_SHIFL	1-6
376	Shigella flexneri 2850-71	Bacteria	PhoP/PhoQ regulator MgrB	I6BSU2_SHIFL	1-6
377	Shigella flexneri 2a str. 301	Bacteria	PhoP/PhoQ regulator MgrB	A0A226K966_SHIFL	1-6
378	Shigella flexneri 4c	Bacteria	PhoP/PhoQ regulator MgrB	A0A127GKV5_SHIFL	1-6
379	Shigella flexneri 5a str. M90T	Bacteria	PhoP/PhoQ regulator MgrB	A0A0F6MCX8_SHIFL	1-6
380	Shigella flexneri K-227	Bacteria	PhoP/PhoQ regulator MgrB	F5NUD0_SHIFL	1-6
381	Shigella flexneri K-315	Bacteria	PhoP/PhoQ regulator MgrB	I6CV84_SHIFL	1-6
382	Shigella flexneri serotype 5b (strain 8401)	Bacteria	PhoP/PhoQ regulator MgrB	MGRB_SHIF8	1-6

383	Shigella flexneri serotype X (strain 2002017)	Bacteria	PhoP/PhoQ regulator MgrB	D2AEJ6_SHIF2	1-6
384	Shigella flexneri VA-6	Bacteria	PhoP/PhoQ regulator MgrB	F5N2I2_SHIFL	1-6
385	Shigella sonnei	Bacteria	PhoP/PhoQ regulator MgrB	A0A1S9JSK5_SHISO	1-6
386	Shigella sonnei (strain Ss046)	Bacteria	PhoP/PhoQ regulator MgrB	MGRB_SHISS	1-6
387	Shigella sp. FC2928	Bacteria	PhoP/PhoQ regulator MgrB	A0A1E2VFN8_9ENTR	1-6
388	Shigella sp. FC569	Bacteria	PhoP/PhoQ regulator MgrB	A0A1E3N701_9ENTR	1-6
389	Shigella sp. PAMC 28760	Bacteria	PhoP/PhoQ regulator MgrB	A0A142H3S0_9ENTR	1-6
390	uncultured Ruminococcus sp.	Bacteria	Ribosomal RNA large subunit methyltransferase Cfr	A0A1C6I7L3_9FIRM	1-6
391	Variovorax sp. PDC80	Bacteria	Tripartite-type tricarboxylate transporter, receptor component TctC	A0A1I5TAZ8_9BURK	5-11
392	Vibrio neptunius	Bacteria	Lipoprotein	A0A0F4PIN6_9VIBR	1-6
393	Ananas comosus (Pineapple) (Ananas ananas)	Eukaryota	Uncharacterized protein	A0A199VQR6_ANACO	138-144
394	Aspergillus udagawae	Eukaryota	Activator of stress genes 1	A0A0K8LE29_9EURO	536-542
395	Basidiobolus meristosporus CBS 931.73	Eukaryota	Uncharacterized protein	A0A1Y1Y869_9FUNG	471-477
396	Gonapodya prolifera JEL478	Eukaryota	Glycosyltransferase family 90 protein	A0A139AIQ7_GONPR	185-192
397	Hypsibius dujardini (Water bear) (Macrobiotus dujardini)	Eukaryota	Dynein heavy chain, cytoplasmic	A0A1W0XD38_HYPDU	469-475
398	Neosartorya fischeri (strain ATCC 1020 / DSM 3700 / CBS 544.65 / FGSC A1164 / JCM 1740 / NRRL 181 / WB 181) (Aspergillus fischerianus)	Eukaryota	Fungal specific transcription factor, putative	A1DCF3_NEOFI	545-552
399	Papilio xuthus (Asian swallowtail butterfly)	Eukaryota	Facilitated trehalose transporter Tret1	A0A194QJ30_PAPXU	268-274
400	Pseudogymnoascus sp. VKM F-3775	Eukaryota	Uncharacterized protein	A0A094ACU4_9PEZI	323-329
401	Ramazzottius varieornatus (Water bear) (Tardigrade)	Eukaryota	Uncharacterized protein	A0A1D1V658_RAMVA	211-217
402	Ramazzottius varieornatus (Water bear) (Tardigrade)	Eukaryota	Uncharacterized protein	A0A1D1VJZ1_RAMVA	466-472
403	Trichinella britovi (Parasitic roundworm)	Eukaryota	Uncharacterized protein	A0A0V1CJ65_TRIBR	299-305
404	Trichinella murrelli	Eukaryota	Uncharacterized protein	A0A0V0TR46_9BILA	299-305

405	Trichinella nativa	Eukaryota	Uncharacterized protein	A0A1Y3ELC3_9BILA	299-305
406	Trichinella nativa	Eukaryota	Uncharacterized protein	A0A0V1LHN3_9BILA	299-305
407	Trichinella nelsoni	Eukaryota	Uncharacterized protein	A0A0V0S3G2_9BILA	299-305
408	Trichinella papuae	Eukaryota	Uncharacterized protein	A0A0V1MXS9_9BILA	299-305
409	Trichinella patagoniensis	Eukaryota	Uncharacterized protein	A0A0V1AC43_9BILA	299-305
410	Trichinella pseudospiralis (Parasitic roundworm)	Eukaryota	Uncharacterized protein	A0A0V1FGT6_TRIPS	299-305
411	Trichinella pseudospiralis (Parasitic roundworm)	Eukaryota	Uncharacterized protein	A0A0V0Y9P6_TRIPS	299-305
412	Trichinella sp. T6	Eukaryota	Uncharacterized protein	A0A0V0WK83_9BILA	299-305
413	Trichinella sp. T8	Eukaryota	Uncharacterized protein	A0A0V1NSD5_9BILA	299-305
414	Trichinella sp. T9	Eukaryota	Uncharacterized protein	A0A0V0UTC4_9BILA	299-305
415	Trichinella spiralis (Trichina worm)	Eukaryota	Uncharacterized protein	E5SRB6_TRISP	299-305
416	Trichinella zimbabwensis	Eukaryota	Uncharacterized protein	A0A0V1HLL3_9BILA	299-305
417	marine sediment metagenome		Uncharacterized protein	A0A0F9K866_9ZZZZ	15-22
