# Supplementary material for: Bacterial MgrB peptide activates chemoreceptor Fpr3 in mouse accessory olfactory system and drives avoidance behaviour
Source: Nat Commun. 2019 Oct 25;10:4889. doi: 10.1038/s41467-019-12842-x (PMC6814738; doi:10.1038/s41467-019-12842-x)
Supplement: Supplementary file 5 — Supplementary Data 3 [file 41467_2019_12842_MOESM5_ESM.pdf]

Supplementary Data 3 | List of organisms (n=350, UniProt) with a full-length MgrB protein.

	Strain	Accession	Biosafety level	Complete MgrB protein sequence	Length
1	Cedecea davisae DSM 4568	S3IR56	2	MRKYRWVIVL VIVVACLLLW TQMINVMCDQ DVQFFSGICT INKFIPW	47
2	Citrobacter amalonaticus Y19	M1KGH3	2	MKKFRWIILV MVVLVCLLLW AQVFNIMCDQ DVQFFSGICA INKFIPW	47
3	Citrobacter braakii	A0A1R0FT00	2	MKKIRWVVLI VVVLVCILMW AQVFNIMCDQ DVQFFSGICA FNKFIPW	47
4	Citrobacter farmeri	A0A223JSW8	2	MKKFRWIILV VVVLVCLLLW AQVFNIMCDQ DVQFFSGICA INKFIPW	47
5	Citrobacter koseri (Citrobacter diversus)	A0A1Z3Y021	2	MKKFRWVILI IVALVCLLLW AQVFNIMCDQ DVQFFNGICA INKFIPW	47
6	Citrobacter koseri (strain ATCC BAA-895 / CDC 4225-83 / SGSC4696)	A8AFM8	2	MKKFRWVILI IVALVCLLLW AQVFNIMCDQ DVQFFNGICA INKFIPW	47
7	Citrobacter pasteurii	A0A0A1RC41	2	MKKFRWVVLI VVVLVCILMW AQVFNIMCDQ DVQFFSGICA INKFIPW	47
8	Cronobacter sakazakii (Enterobacter sakazakii)	A0A0F6VUC5	2	MKKFRWAILL AVLVACLLLW MQTLNVMCDQ DVQFFSGICT INKFIPW	47
9	Cronobacter sakazakii (strain ATCC BAA-894) (Enterobacter sakazakii)	A7MNL7	2	MKKFRWAILL AVLVACLLLW MQTLNVMCDQ DVQFFSGICT INKFIPW	47
10	Cronobacter sakazakii 696	K8D9J2	2	MKKFRWAILL AVLVACLLLW MQTLNVMCDQ DVQFFSGICT INKFIPW	47
11	Cronobacter sakazakii 701	K8CDK0	2	MKKFRWAILL AVLVACLLLW MQTLNVMCDQ DVQFFSGICT INKFIPW	47
12	Cronobacter turicensis (strain DSM 18703 / LMG 23827 / z3032)	C9XUH3	2	MKKFRWAILL AVLVACLLLW TQTINVMCDQ DVQFFSGICT INKFIPW	47
13	Cronobacter turicensis 564	K8B7R0	2	MKKFRWAILL AVLVACLLLW TQTINVMCDQ DVQFFSGICT INKFIPW	47
14	Cronobacter universalis NCTC 9529	K8DBV6	1	MKKFRWAILL AVLVACLLLW TQTINVMCDQ DVQFFSGICT INKFIPW	47
15	Enterobacter asburiae	A0A0D9H1Q3	2	MKKIRWVILI IVLIACVVLW TQTINVMCDQ DVQFFSGVCA INKFIPW	47
16	Enterobacter asburiae (strain LF7a)	G2S8L6	2	MKKIRWVILV IVLIACVVLW TQTINVMCDQ DVQFFSGVCA INKFIPW	47
17	Enterobacter cancerogenus	A0A1V6M7X4	2	MKKIRWVILI IVLIACVVLW TQTINVMCDQ DVQFFSGVCA INKFIPW	47
18	Enterobacter cancerogenus ATCC 35316	D2ZDJ7	2	MKKIRWVILI IVLIACVVLW TQTINVMCDQ DVQFFSGVCA INKFIPW	47
19	Enterobacter cloacae	A0A0A6EEV1	2	MKKIRWVILI IVLIACVVLW TQTINVMCDQ DVQFFSGICS INKFIPW	47
20	Enterobacter cloacae	A0A1B3F289	2	MKKIRWVILV IVLIACVVLW TQTINVMCDQ DVQFFSGVCA INKFIPW	47

21	Enterobacter cloacae	A0A144H4A4	**2**	MKKIRWVILI IVLIACVVLW TQTINVMCDQ DVQFFSGVCA INKFIPW	47
22	Enterobacter cloacae	A0A0M3F5S4	**2**	MKKLRWVILV VVLIACVVMW TQMINVMCDQ DVQFFSGICS INKFIPW	47
23	Enterobacter cloacae	A0A176WX09	**2**	MKKIRWVILV IVLIACVVLW TQTINVMCDQ DVQFFSGICS INKFIPW	47
24	Enterobacter cloacae	A0A155YV94	**2**	MKKIRWVVLI VVVLVCILMW AQVFNIMCDQ DVQFFSGICA VNKFIPW	47
25	Enterobacter cloacae	A0A1T1G664	**2**	MKKIRWVILI VVLIACVVLW TQTINVMCDQ DVQFFSGVCA INKFIPW	47
26	Enterobacter cloacae complex 'Hoffmann cluster IV'	A0A167PNN6	**2**	MKKIRWVILI IVLIACVVLW TQTINVMCDQ DVQFFSGVCA INKFIPW	47
27	Enterobacter cloacae complex sp. CIDEIMsCOL9	A0A0F4BFW7	**2**	MKKIRWVILI IVLIACVVLW TQTINVMCDQ DVQFFSGVCA INKFIPW	47
28	Enterobacter cloacae complex sp. GN02468	A0A0X6Z7Y0	**2**	MKKIRWVILI IVLIACVVLW TQTINVMCDQ DVQFFSGVCA INKFIPW	47
29	Enterobacter cloacae complex sp. GN04363	A0A0X4ECZ3	**2**	MKKIRWVILI IVLIACVVLW TQTINVMCDQ DVQFFSGVCA INKFIPW	47
30	Enterobacter cloacae complex sp. GN04787	A0A167JEK4	**2**	MKKIRWVILI IVLIACVVLW TQTINVMCDQ DVQFFSGVCA INKFIPW	47
31	Enterobacter cloacae complex sp. GN06232	A0A162TV43	**2**	MKKIRWVILI IVLIACVVLW TQTINVMCDQ DVQFFSGICS INKFIPW	47
32	Enterobacter cloacae complex sp. SMART_763	A0A0W2HGZ5	**2**	MKKIRWVILI IVLIACVVLW TQTINVMCDQ DVQFFSGVCA INKFIPW	47
33	Enterobacter cloacae EcWSU1	G8LG91	**2**	MKKIRWVILI IVLVACVVLW TQTINVMCDQ DVQFFSGVCA INKFIPW	47
34	Enterobacter cloacae P101	W0BI86	**2**	MKKIRWVILI IVLVACVVLW TQTINVMCDQ DVQFFSGVCA INKFIPW	47
35	Enterobacter cloacae S611	V5ABZ6	**2**	MRKFRWLLLI AVVVVCLLLW TQMINVMCDQ DVQFFSGICV INKFIPW	47
36	Enterobacter cloacae subsp. cloacae	A0A0F0TLM8	**2**	MKKIRWVILV IVLIACVVLW TQTINVMCDQ DVQFFSGVCA INKFIPW	47
37	Enterobacter cloacae subsp. cloacae	A0A246PDZ8	**2**	MKKIRWVILI VVLIACVVLW TQTINVMCDQ DVQFFSGVCA INKFIPW	47
38	Enterobacter cloacae subsp. cloacae (strain ATCC 13047 / DSM 30054 / NBRC 13535 / NCDC 279-56)	A0A0H3CID4	**2**	MKKIRWVILV IVLIACVVLW TQTINVMCDQ DVQFFSGVCA INKFIPW	47
39	Enterobacter cloacae subsp. dissolvens SDM	A0A0M1R669	**2**	MKKIRWVILV IVLIACVVLW TQTINVMCDQ DVQFFSGVCA INKFIPW	47
40	Enterobacter cloacae UCICRE 3	A0A0M2BYV2	**2**	MKKIRWVILI IVLIACVVLW TQTINVMCDQ DVQFFSGICS INKFIPW	47
41	Enterobacter hormaechei	A0A246LH38	**2**	MKKIRWVILI IVLIACVVLW TQTINVMCDQ DVQFFSGVCA INKFIPW	47
42	Enterobacter hormaechei ATCC 49162	F5RYS1	**2**	MKKIRWVILI IVLIACVVLW TQTINVMCDQ DVQFFSGICS INKFIPW	47

43	Enterobacter hormaechei subsp. oharae	A0A0W1WVY1	**2**	MKKIRWVILI IVLIACVVLW TQTINVMCDQ DVQFFSGICS INKFIPW	47
44	Enterobacter hormaechei subsp. steigerwaltii	A0A0E1JEQ8	**2**	MKKIRWVILI IVLIACVVLW TQTINVMCDQ DVQFFSGICS INKFIPW	47
45	Enterobacter kobei	A0A0F0Y7A0	**2**	MKKIRWVILI VVLIACVVLW TQTINVMCDQ DVQFFSGVCA INKFIPW	47
46	Enterobacter kobei	A0A242VUR2	**2**	MKKIRWVILI IVLIACVVLW TQTINVMCDQ DVQFFSGVCA INKFIPW	47
47	Enterobacter kobei	A0A1N6WEK5	**2**	MRKFRWLIVI AALVACLLLW TQMLNVMCDQ DVQFFSGICV INKFIPW	47
48	Enterobacter mori	A0A229HZG0	**1**	MKKIRWVILV IVLVACVVLW TQTINVMCDQ DVQFFSGVCA INKFIPW	47
49	Enterobacter soli ATCC BAA-2102	A0A198GFR2	**?**	MKKIRWVILV IVLIACVVLW TQTINVMCDQ DVQFFSGVCA INKFIPW	47
50	Enterobacter sp. (strain 638)	A4WBI4	**2**	MKKIRWVILI IVLIACVILW TQTINVMCDQ DVQFFSGICA INQFIPW	47
51	Enterobacter sp. 50588862	A0A0V9ECL0	**2**	MKKIRWVILI IVLIACVVLW TQTINVMCDQ DVQFFSGVCA INKFIPW	47
52	Enterobacter sp. Ag1	J1GJG0	**2**	MRKYRWVIVL VIVVTCLLLW TQMINVMCDQ DVQFFSGICT INKFIPW	47
53	Enterobacter sp. GN02600	A0A0J0IYM1	**2**	MKKIRWVVLI VVVLVCILMW AQVFNIMCDQ DVQFFSGICA FNKFIPW	47
54	Enterobacter sp. ku-bf2	A0A1E5MWS5	**2**	MKKIRWVILI IVLIACVVLW TQTINVMCDQ DVQFFSGVCA INKFIPW	47
55	Enterobacter sp. NFIX03	A0A1K2DDQ1	**2**	MRKYKWLILI VVLVGCLLLW TQMLNVMCDQ DVQFFSGICI VNKFIPW	47
56	Enterobacteriaceae bacterium (strain FGI 57)	L0M508	**2**	MKKFRWVLLI VVLAACLLLW TQTLNVLCDQ DVQFFNGICS INKFIPW	47
57	Escherichia albertii	A0A0S3NKU5	**2**	MKKFRWVVLA VAVLACLLLW MQVFSMMCDQ DVPFFSGICA INQLIPW	47
58	Escherichia albertii (strain TW07627)	B1EQ07	**2**	MKKFRWVVLA VAVLACLLLW MQVFSMMCDQ DVPFFSGICA INQLIPW	47
59	Escherichia coli	C3T687	**?**	MKKFRWVVLV VVVLACLLLW AQVFNMMCDQ DVQFFSGICA INQFIPW	47
60	Escherichia coli	A0A0T5XJZ7	**?**	MKKIRWVILI IVLIACVVLW TQTINVMCDQ DVQFFSGICS INKFIPW	47
61	Escherichia coli	E2QN28	**?**	MKKFRWVALV VVVLACLLLW AQVFNMMCDQ DVQFFSGICA INQFIPW	47
62	Escherichia coli	A0A0B1MU87	**?**	MKKFRWVVLV VVVLACLLLW VQVFNMMCDQ DVQFFSGICA INQFIPW	47
63	Escherichia coli	A0A245NPM2	**?**	MKKFRWVALV VVVLACLLLW AQVFNMMCDQ DVQFFSGICA LNQFIPW	47
64	Escherichia coli	C3T690	**?**	MKKFRWVVLV VVVLACLLLW AQVFNMMCDQ DVQFFSGICA INQFISW	47
65	Escherichia coli	A0A166SZZ7	**2**	MKKFRWGVLV VAVLACLLLW AQVFNMMCDQ DVQFFNGICT INQFIPW	47
66	Escherichia coli (strain 55989 / EAEC)	B7L6V5	**2**	MKKFRWVVLV VVVLACLLLW AQVFNMMCDQ DVQFFSGICA INQFIPW	47
67	Escherichia coli (strain ATCC 8739 / DSM 1576 / Crooks)	B1J0R3	**1**	MKKFRWVVLV VVVLACLLLW AQVFNMMCDQ DVQFFSGICA INQFIPW	47

68	Escherichia coli (strain ATCC 9637 / CCM 2024 / DSM 1116 / NCIMB 8666 / NRRL B-766 / W)	E8PVX6	**1**	MKKFRWVVLV VVVLACLLLW AQVFNMMCDQ DVQFFSGICA INQFIPW	47
69	Escherichia coli (strain B / BL21-DE3)	A0A140N8W7	**1**	MKKFRWVVLV VVVLACLLLW AQVFNMMCDQ DVQFFSGICA INQFIPW	47
70	Escherichia coli (strain K12 / DH10B)	B1XH93	**1**	MKKFRWVVLV VVVLACLLLW AQVFNMMCDQ DVQFFSGICA INQFIPW	47
71	Escherichia coli (strain K12 / MC4100 / BW2952)	C4ZZI2	**1**	MKKFRWVVLV VVVLACLLLW AQVFNMMCDQ DVQFFSGICA INQFIPW	47
72	Escherichia coli (strain K12)	P64512	**1**	MKKFRWVVLV VVVLACLLLW AQVFNMMCDQ DVQFFSGICA INQFIPW	47
73	Escherichia coli (strain SE11)	B6IBQ3	**?**	MKKFRWVVLV VVVLACLLLW AQVFNMMCDQ DVQFFSGICA INQFIPW	47
74	Escherichia coli (strain SMS-3-5 / SECEC)	B1LD47	**?**	MKKFRWVVLV VVVLACLLLW AQVFNMMCDQ DVQFFSGICA INQFIPW	47
75	Escherichia coli (strain UTI89 / UPEC)	Q1RAW2	**2**	MKKFRWVALV VVVLACLLLW AQVFNMMCDQ DVQFFSGICA LNQFIPW	47
76	Escherichia coli 1.2264	I2SPL6	**?**	MKKFRWVVLV VVVLACLLLW AQVFNMMCDQ DVQFFSGICA INQFIPW	47
77	Escherichia coli 1.2741	I2RJX3	**?**	MKKFRWVVLV VVVLACLLLW AQVFNMMCDQ DVQFFSGICA INQFIPW	47
78	Escherichia coli 110957	U9YSH3	**?**	MKKFRWVVLV VVVLACLLLW AQVFNMMCDQ DVQFFSGICA INQFIPW	47
79	Escherichia coli 1-110-08_S3_C1	A0A125X2S3	**?**	MKKFRWVVLV VVVLACLLLW AQVFNMMCDQ DVQFFSGICA INQFIPW	47
80	Escherichia coli 113290	U9Z5T2	**?**	MKKFRWVVLV VVVLACLLLW AQVFNMMCDQ DVQFFSGICA INQFIPW	47
81	Escherichia coli 113303	U9XWI6	**?**	MKKFRWVVLV VVVLACLLLW AQVFNMMCDQ DVQFFSGICA INQFIPW	47
82	Escherichia coli 1-176-05_S3_C2	A0A017III5	**?**	MKKFRWGVLV VAVLACLLLW AQVFNMMCDQ DVQFFNGICT INQFIPW	47
83	Escherichia coli 1-250-04_S3_C2	A0A080J2Z5	**?**	MKKFRWVVLV VVVLACLLLW AQVFNMMCDQ DVQFFSGICA INQFIPW	47
84	Escherichia coli 1303	A0A0E1M053	**?**	MKKFRWVVLV VVVLACLLLW AQVFNMMCDQ DVQFFSGICA INQFIPW	47
85	Escherichia coli 1-392-07_S4_C1	A0A074HXH8	**?**	MKKFRWVVLV VVVLACLLLW AQVFNMMCDQ DVQFFSGICA INQFIPW	47
86	Escherichia coli 1-392-07_S4_C3	A0A080FSW3	**?**	MKKFRWVVLV VVVLACLLLW AQVFNMMCDQ DVQFFSGICA INQFIPW	47
87	Escherichia coli 2.3916	I2XER0	**?**	MKKFRWVVLV VVVLACLLLW AQVFNMMCDQ DVQFFSGICA INQFIPW	47
88	Escherichia coli 2-005-03_S4_C2	A0A029IT43	**?**	MKKFRWVALV VVVLACLLLW AQVFNMMCDQ DVQFFSGICA INQFIPW	47
89	Escherichia coli 2-005-03_S4_C3	A0A029HZ23	**?**	MKKFRWVALV VVVLACLLLW AQVFNMMCDQ DVQFFSGICA INQFIPW	47
90	Escherichia coli 2-011-08_S1_C1	A0A062Y005	**?**	MKKFRWVVLV VVVLACLLLW AQVFNMMCDQ DVQFFSGICA INQFIPW	47
91	Escherichia coli 2-177-06_S3_C2	A0A070V2T7	**?**	MKKFRWVVLV VVVLACLLLW AQVFNMMCDQ DVQFFSGICA INQFIPW	47

92	Escherichia coli 2-210-07_S3_C3	A0A070SW86	**?**	MKKFRWVVLV VVVLACLLLW AQVFNMMCDQ DVQFFSGICA INQFIPW	47
93	Escherichia coli 2362-75	E3XP38	**?**	MKKFRWVALV VVVLACLLLW AQVFNMMCDQ DVQFFSGICA INQFIPW	47
94	Escherichia coli 2-427-07_S4_C3	A0A073FWP5	**?**	MKKFRWVVLV VVVLACLLLW AQVFNMMCDQ DVQFFSGICA INQFIPW	47
95	Escherichia coli 3-267-03_S4_C1	A0A073GBN8	**?**	MKKFRWVVLV VVVLACLLLW AQVFNMMCDQ DVQFFSGICA INQFIPW	47
96	Escherichia coli 3-373-03_S4_C2	A0A069XI43	**?**	MKKFRWVVLV VVVLACLLLW AQVFNMMCDQ DVQFFSGICA INQFIPW	47
97	Escherichia coli 4.0522	I2UJ85	**?**	MKKFRWVVLV VVVLACLLLW AQVFNMMCDQ DVQFFSGICA INQFNPW	47
98	Escherichia coli 4.0967	I2WU26	**?**	MKKFRWVVLV VVVLACLLLW AQVFNMMCDQ DVQFFSGICA INQFIPW	47
99	Escherichia coli 53638	A0A0E1T0C7	**?**	MKKFRWVVLV VVVLACLLLW AQVFNMMCDQ DVQFFSGICA INQFIPW	47
100	Escherichia coli 5-366-08_S1_C1	A0A073USV5	**?**	MKKFRWVVLV VVVLACLLLW AQVFNMMCDQ DVQFFSGICA INQFIPW	47
101	Escherichia coli 5-366-08_S1_C3	A0A073HHE3	**?**	MKKFRWVVLV VVVLACLLLW AQVFNMMCDQ DVQFFSGICA INQFIPW	47
102	Escherichia coli 541-15	I4SWD7	**?**	MKKFRWVVLV VVVLACLLLW AQVFNMMCDQ DVQFFSGICA INQFIPW	47
103	Escherichia coli 6-537-08_S1_C3	A0A080FEG9	**?**	MKKFRWVVLV VVVLACLLLW AQVFNMMCDQ DVQFFSGICA INQFIPW	47
104	Escherichia coli 9.0111	I2W471	**?**	MKKFRWVVLV VVVLACLLLW AQVFNMMCDQ DVQFFSGICA INQFIPW	47
105	Escherichia coli 907672	V0S9T0	**?**	MKKFRWVVLV VVVLACLLLW AQVFNMMCDQ DVQFFSGICA INQFIPW	47
106	Escherichia coli 907713	U9Z453	**?**	MKKFRWVVLV VVVLACLLLW AQVFNMMCDQ DVQFFSGICA INQFIPW	47
107	Escherichia coli 908519	V0VLI9	**?**	MKKFRWVALV VVVLACLLLW AQVFNMMCDQ DVQFFSGICA INQFIPW	47
108	Escherichia coli 908525	V0Y445	**?**	MKKFRWVVLV VVVLACLLLW AQVFNMMCDQ DVQFFSGICA INQFIPW	47
109	Escherichia coli 908573	V0ZF78	**?**	MKKFRWVVLV VVVLACLLLW AQVFNMMCDQ DVQFFSGICA INQFIPW	47
110	Escherichia coli 909945-2	U9ZGG3	**?**	MKKFRWVVLV VVVLACLLLW AQVFNMMCDQ DVQFFSGICA INQFIPW	47
111	Escherichia coli 97.0246	I2S468	**?**	MKKFRWVVLV VVVLACLLLW AQVFNMMCDQ DVQFFSGICA INQFIPW	47
112	Escherichia coli 99.0741	V6FR77	**?**	MKKFRWVVLV VVVLACLLLW AQVFNMMCDQ DVQFFSGICA INQFIPW	47
113	Escherichia coli ATCC BAA-2209	V8FM95	**2**	MKKFRWVVLV VVVLACLLLW AQVFNMMCDQ DVQFFSGICA INQFNPW	47
114	Escherichia coli B354	D6JAA5	**?**	MKKFRWVVLV VVVLACLLLW AQVFNMMCDQ DVQFFSGICA INQFIPW	47
115	Escherichia coli chi7122	J7QM34	**?**	MKKFRWVVLV VVVLACLLLW AQVFNMMCDQ DVQFFSGICA INQFIPW	47
116	Escherichia coli D6-113.11	A0A024KXF8	**?**	MKKFRWVVLV VVVLACLLLW AQVFNMMCDQ DVQFFSGICA INQFIPW	47
117	Escherichia coli DEC1B	H4IBV5	**2**	MKKFRWVALV VVVLACLLLW AQVFNMMCDQ DVQFFSGICA INQFIPW	47
118	Escherichia coli DEC1C	H4ISR4	**2**	MKKFRWVALV VVVLACLLLW AQVFNMMCDQ DVQFFSGICA INQFIPW	47
119	Escherichia coli DEC1D	H4J911	**2**	MKKFRWVALV VVVLACLLLW AQVFNMMCDQ DVQFFSGICA INQFIPW	47

120	Escherichia coli DEC2C	H4KHU7	**2**	MKKFRWVALV VVVLACLLLW AQVFNMMCDQ DVQFFSGICA INQFIPW	47
121	Escherichia coli DEC2E	H4LCR2	**2**	MKKFRWVALV VVVLACLLLW AQVFNMMCDQ DVQFFSGICA INQFIPW	47
122	Escherichia coli DEC6A	H4UI03	**2**	MKKFRWVVLV VVVLACLLLW AQVFNMMCDQ DVQFFSGICA INQFIPW	47
123	Escherichia coli E1114	A0A1X3IN00	**?**	MKKFRWVVLV VVVLACLLLW AQVFNMMCDQ DVQFFSGICA INQFIPW	47
124	Escherichia coli E1118	A0A1X3MBX5	**?**	MKKFRWVVLV VAVLACLLLW AQVFNMMCDQ DVQFFSGICA INQFIPW	47
125	Escherichia coli G3/10	A0A0A0FHQ3	**1**	MKKFRWVVLV VVVLACLLLW AQVFNMMCDQ DVQFFSGICA INQFIPW	47
126	Escherichia coli H386	A0A1X3JGR7	**?**	MKKFRWVVLV VVVLACLLLW AQVFNMMCDQ DVQFFSGICA INQFIPW	47
127	Escherichia coli H461	A0A1X3KBM9	**?**	MKKFRWVALV VVVLACLLLW AQVFNMMCDQ DVQFFSGICA LNQFIPW	47
128	Escherichia coli H591	F4VG73	**?**	MKKFRWVVLV VVVLACLLLW AQVFNMMCDQ DVQFFSGICA INQFIPW	47
129	Escherichia coli H605	A0A1X3KRT6	**?**	MKKFRWGVLV VAVLACLLLW AQVFNMMCDQ DVQFFNGICT INQFIPW	47
130	Escherichia coli H736	F4SKX9	**?**	MKKFRWVVLV VVVLACLLLW AQVFNMMCDQ DVQFFSGICA INQFIPW	47
131	Escherichia coli ISC7	W1EVS7	**?**	MKKFRWVVLV VVVLACLLLW AQVFNMMCDQ DVQFFSGICA INQFIPW	47
132	Escherichia coli LAU-EC10	V8KE55	**?**	MKKFRWVVLV VVVLACLLLW AQVFNMMCDQ DVQFFSGICA INQFIPW	47
133	Escherichia coli M056	A0A1X3I7C9	**?**	MKKFRWVVLV VVVLACLLLW AQVFNMMCDQ DVQFFSGICA INQFIPW	47
134	Escherichia coli M605	F4SZ56	**?**	MKKFRWVALV VVVLACLLLW AQVFNMMCDQ DVQFFSGICA INQFIPW	47
135	Escherichia coli M718	F4TFF4	**?**	MKKFRWVVLV VVVLACLLLW AQVFNMMCDQ DVQFFSGICA INQFIPW	47
136	Escherichia coli M8	A0A1S6TBH1	**?**	MKKFRWVVLV VVVLACLLLW AQVFNMMCDQ DVQFFSGICA INQFIPW	47
137	Escherichia coli M863	E9YP17	**?**	MKKFRWVVLV VVVLACLLLW AQVFNMMCDQ DVQFFSGICA INQFIPW	47
138	Escherichia coli MP021561.2	M9H086	**?**	MKKFRWVVLV VVVLACLLLW AQVFNMMCDQ DVQFFSGICA INQFIPW	47
139	Escherichia coli MS 119-7	D8EC48	**?**	MKKFRWVVLV VVVLACLLLW AQVFNMMCDQ DVQFFSGICA INQFIPW	47
140	Escherichia coli MS 124-1	E1JC52	**?**	MKKFRWVVLV VVVLACLLLW AQVFNMMCDQ DVQFFSGICA INQFIPW	47
141	Escherichia coli MS 145-7	E1IML7	**?**	MKKFRWVVLV VVVLACLLLW AQVFNMMCDQ DVQFFSGICA INQFIPW	47
142	Escherichia coli MS 85-1	E6BEP1	**?**	MKKFRWVVLV VVVLACLLLW AQVFNMMCDQ DVQFFSGICA INQFIPW	47
143	Escherichia coli O1:K1 / APEC	P0C7B2	**2**	MKKFRWVALV VVVLACLLLW AQVFNMMCDQ DVQFFSGICA LNQFIPW	47
144	Escherichia coli O103:H2 (strain 12009 / EHEC)	C8UA84	**3**	MKKFRWVVLV VVVLACLLLW AQVFNMMCDQ DVQFFSGICA INQFIPW	47
145	Escherichia coli O104:H4 (strain 2011C-3493)	A0A0E0XY13	**2**	MKKFRWVVLV VVVLACLLLW AQVFNMMCDQ DVQFFSGICA INQFIPW	47

146	Escherichia coli O111:H- (strain 11128 / EHEC)	C8UBS5	**3**	MKKFRWVVLV VVVLACLLLW AQVFNMMCDQ DVQFFSGICA INQFNPW	47
147	Escherichia coli O111:H11 str. CVM9455	K4WG83	**2**	MKKFRWVVLV VVVLACLLLW AQVFNMMCDQ DVQFFSGICA INQFIPW	47
148	Escherichia coli O111:H8 str. CVM9634	K4V6M8	**2**	MKKFRWVVLV VVVLACLLLW AQVFNMMCDQ DVQFFSGICA INQFNPW	47
149	Escherichia coli O111:NM str. K6722	A0A027TT17	**2**	MKKFRWVVLV VVVLACLLLW AQVFNMMCDQ DVQFFSGICA INQFNPW	47
150	Escherichia coli O118:H16 str. 2009C-4446	A0A028E2J5	**2**	MKKFRWVVLV VVVLACLLLW AQVFNMMCDQ DVQFFSGICA INQFIPW	47
151	Escherichia coli O121:H19 str. 2010C-3609	A0A0E2TSV4	**2**	MKKFRWVVLV VVVLACLLLW AQVFNMMCDQ DVQFFSGICA INQFIPW	47
152	Escherichia coli O127:H6 (strain E2348/69 / EPEC)	B7USK2	**2**	MKKFRWVALV VVVLACLLLW AQVFNMMCDQ DVQFFSGICA INQFIPW	47
153	Escherichia coli O128:H2 str. 2011C-3317	A0A070ETA6	**2**	MKKFRWVVLV VVVLACLLLW AQVFNMMCDQ DVQFFSGICA INQFIPW	47
154	Escherichia coli O139:H28 (strain E24377A / ETEC)	A7ZMU8	**2**	MKKFRWVVLV VVVLACLLLW AQVFNMMCDQ DVQFFSGICA INQFIPW	47
155	Escherichia coli O145:H25 str. 07-3858	A0A070D363	**3**	MKKFRWVVLV VVVLACLLLW AQVFNMMCDQ DVQFFSGICA INQFIPW	47
156	Escherichia coli O145:H28 str. RM12581	A0A023YXY9	**3**	MKKFRWVVLV VVVLACLLLW AQVFNMMCDQ DVQFFSGICA INQFIPW	47
157	Escherichia coli O145:NM str. 2010C-3526	A0A025CNG5	**2**	MKKFRWVVLV VVVLACLLLW AQVFNMMCDQ DVQFFSGICA INQFIPW	47
158	Escherichia coli O146:H21 str. 2010C-3325	A0A070CRX4	**2**	MKKFRWVVLV VVVLACLLLW AQVFNMMCDQ DVQFFSGICA INQFIPW	47
159	Escherichia coli O157	A0A1Z3UR72	**3**	MKKFRWVVLV VVVLACLLLW AQVFNMMCDQ DVQFFSGICA INQFIPW	47
160	Escherichia coli O157:H7	P64513	**3**	MKKFRWVVLV VVVLACLLLW AQVFNMMCDQ DVQFFSGICA INQFIPW	47
161	Escherichia coli O157:H7 (strain EC4115 / EHEC)	B5YQW8	**3**	MKKFRWVVLV VVVLACLLLW AQVFNMMCDQ DVQFFSGICA INQFIPW	47
162	Escherichia coli O157:H7 str. SS52	A0A0F6F7I3	**3**	MKKFRWVVLV VVVLACLLLW AQVFNMMCDQ DVQFFSGICA INQFIPW	47
163	Escherichia coli O17:K52:H18 (strain UMN026 / ExPEC)	B7NBH3	**2**	MKKFRWVVLV VVVLACLLLW AQVFNMMCDQ DVQFFSGICA INQFIPW	47
164	Escherichia coli O174:H8 str. 04-3038	A0A026V3K9	**?**	MKKFRWVVLV VVVLACLLLW AQVFNMMCDQ DVQFFSGICA INQFIPW	47
165	Escherichia coli O25b:H4	A0A192C884	**?**	MKKFRWVALV VVVLACLLLW AQVFNMMCDQ DVQFFSGICA INQFIPW	47

166	Escherichia coli O25b:H4-ST131	W8ZJQ3	**2**	MKKFRWVALV VVVLACLLLW AQVFNMMCDQ DVQFFSGICA INQFIPW	47
167	Escherichia coli O45:K1 (strain S88 / ExPEC)	B7MBN2	**2**	MKKFRWVALV VVVLACLLLW AQVFNMMCDQ DVQFFSGICA LNQFIPW	47
168	Escherichia coli O55:H7 (strain CB9615 / EPEC)	D3QVU2	**2**	MKKFRWVVLV VVVLACLLLW AQVFNMMCDQ DVQFFSGICA INQFISW	46
169	Escherichia coli O6:H1 (strain CFT073 / ATCC 700928 / UPEC)	Q8FGT7	**2**	MKKFRWVALV VVVLACLLLW AQVFNMMCDQ DVQFFSGICA INQFIPW	47
170	Escherichia coli O6:K15:H31 (strain 536 / UPEC)	P0C7B3	**2**	MKKFRWVALV VVVLACLLLW AQVFNMMCDQ DVQFFSGICA INQFIP	47
171	Escherichia coli O69:H11 str. 08-4661	A0A027ZLA9	**?**	MKKFRWVVLV VVVLACLLLW AQVFNMMCDQ DVQFFSGICA INQFIPW	47
172	Escherichia coli O7:K1 str. CE10	A0A0E0V651	**?**	MKKFRWVVLV VVVLACLLLW AQVFNMMCDQ DVQFFSGICA INQFIPW	47
173	Escherichia coli O78:H11 (strain H10407 / ETEC)	E3PNL1	**2**	MKKFRWVVLV VVVLACLLLW AQVFNMMCDQ DVQFFSGICA INQFIPW	47
174	Escherichia coli O8 (strain IAI1)	B7M2A1	**1**	MKKFRWVVLV VVVLACLLLW AQVFNMMCDQ DVQFFSGICA INQFIPW	47
175	Escherichia coli O81 (strain ED1a)	B7MVV5	**1**	MKKFRWVALV VVVLACLLLW AQVFNMMCDQ DVQFFSGICA INQFIPW	47
176	Escherichia coli O83:H1 (strain NRG 857C / AIEC)	A0A0H3EIC2	**2**	MKKFRWVALV VVVLACLLLW AQVFNMMCDQ DVQFFSGICA INQFIPW	47
177	Escherichia coli O9:H4 (strain HS)	A8A123	**?**	MKKFRWVVLV VVVLACLLLW AQVFNMMCDQ DVQFFSGICA INQFIPW	47
178	Escherichia coli P0301867.5	N4P199	**?**	MKKFRWVVLV VVVLACLLLW AQVFNMMCDQ DVQFFSGICA INQFIPW	47
179	Escherichia coli PCN033	A0A0G3K4U9	**?**	MKKFRWVVLV VVVLACLLLW AQVFNMMCDQ DVQFFSGICA INQFIPW	47
180	Escherichia coli TA249	A0A1X3LYF4	**?**	MKKFRWVVLV VVVLACLLLW AQVFNMMCDQ DVQFFSGICA INQFIPW	47
181	Escherichia coli TA280	F4V2X8	**?**	MKKFRWGVLV VVVLACLLLW AQVFNMMCDQ DVQFFSGICA INQFIPW	47
182	Escherichia coli TW10509	E9XJ20	**?**	MKKFRWVVLV VVVLACLLLW AQVFNMMCDQ DVQFFSGICA INQFIPW	47
183	Escherichia coli UMNF18	G0F6U3	**?**	MKKFRWVVLV VVVLACLLLW AQVFNMMCDQ DVQFFSGICA INQFIPW	47
184	Escherichia coli UMNK88	A0A0E0TZ74	**?**	MKKFRWVVLV VVVLACLLLW AQVFNMMCDQ DVQFFSGICA INQFIPW	47
185	Escherichia coli W26	I0VLI4	**?**	MKKFRWVVLV VVVLACLLLW AQVFNMMCDQ DVQFFSGICA INQFIPW	47
186	Escherichia coli Xuzhou21	A0A0F6C5B9	**3**	MKKFRWVVLV VVVLACLLLW AQVFNMMCDQ DVQFFSGICA INQFIPW	47
187	Escherichia fergusonii	A0A0W3CUM4	**2**	MKKFRWVALV VVVLACLLLW AQVFNMMCDQ DVQFFSGICA INQFIPW	47
188	Escherichia fergusonii (strain ATCC 35469 / DSM 13698 / CDC 0568-73)	B7LPM0	**2**	MKKFRWLVLI VVVLACLVLW AQVINIMCDQ DVQFFSGICA INKFIPW	47

189	Franconibacter pulveris	A0A0J8VIN5	**2**	MKKFRWAILL AVLVACLLLW TQMLNVMCDQ DVQFFSGICT INKFIPW	47
190	Klebsiella aerogenes (Enterobacter aerogenes)	A0A145NJA0	**2**	MKKIRWVILI IVLIACVVLW TQTINVMCDQ DVQFFSGICS INKFIPW	47
191	Klebsiella aerogenes (Enterobacter aerogenes)	A0A0B2IKY1	**2**	MKKLRWVLLI VVIAGCLLLW TQMLNVMCDQ DVQFFSGICA INKFIPW	47
192	Klebsiella aerogenes (strain ATCC 13048 / DSM 30053 / JCM 1235 / KCTC 2190 / NBRC 13534 / NCIMB 10102 / NCTC 10006) (Enterobacter aerogenes)	A0A0H3G2N0	**2**	MKKLRWVLLI VVIAGCLLLW TQMLNVMCDQ DVQFFSGICA INKFIPW	47
193	Klebsiella michiganensis	A0A1Q8YNF7	**2**	MKKLRWVLLI VIIAGCLLLW TQMLNVMCDQ DVQFFSGICT INKFIPW	47
194	Klebsiella oxytoca	A0A0G3S8M8	**2**	MKKLRWVLLI VIIAGCLLLW TQMLNVMCDQ DVQFFSGICT INKFIPW	47
195	Klebsiella oxytoca (strain ATCC 8724 / DSM 4798 / JCM 20051 / NBRC 3318 / NRRL B-199 / KCTC 1686)	A0A0H3HIQ0	**2**	MKKLRWVLLI VIIAGCLLLW TQMLNVMCDQ DVQFFSGICT INKFIPW	47
196	Klebsiella oxytoca KONIH1	A0A068HKC3	**2**	MKKLRWVLLI VIIAGCLLLW TQMLNVMCDQ DVQFFSGICT INKFIPW	47
197	Klebsiella pneumoniae	A0A0E4AX29	**2**	MKKLRWVLLI VIIAGCLLLW TQMLNVMCDQ DVQFFSGICT INKFIPW	47
198	Klebsiella pneumoniae	A0A097F1M8	**2**	MKKLRWVLLI VIISGCLLLW TQMLNVMCDQ DVQFFSGICT INKFIPW	47
199	Klebsiella pneumoniae	A0A1X6DDH5	**2**	MKKLRWVLLI VIIAGCLLLW TQMLNVMCDQ DVQFFSGICT INKFISW	47
200	Klebsiella pneumoniae	A0A1C6ZWB0	**2**	MKKLRWVLLI VIIAGCLLLW TQMLNVMCDQ DVQFFSGICT IDKFIPW	47
201	Klebsiella pneumoniae	A0A0E4AX28	**2**	MKKLRWVLLI VIIAGCLLLW TQMLNVMCDQ DVQFFSGICT INKFIPWYDI FLPTDFLLRR GW	47
202	Klebsiella pneumoniae	A0A0E4AWC5	**2**	MKKLRWVLLI VIIAGCLLLW TQMLNVMCDQ DVQFFSGICT INKFIPWYDI FLTTDFLLRR GW	47
203	Klebsiella pneumoniae	A0A097F1P8	**2**	MKKLRWVLLI VIIAGCLLLW TQMLNVMFDQ DVQFFSGICT INKFIPW	47
204	Klebsiella pneumoniae (strain 342)	B5XQ45	**2**	MKKLRWVLLI VIIAGCLLLW TQMLNVMCDQ DVQFFSGICT INKFIPW	47
205	Klebsiella pneumoniae 909957	V0B1N8	**2**	MKKLRWVLLI VIIAGCLLLW TQMLNVMCDQ DVQFFSGICT INKFIPW	47
206	Klebsiella pneumoniae IS22	W1B6K7	**2**	MKKFRWVVLV VVVLACLLLW AQVFNMMCDQ DVQFFSGICA INQFIPW	47
207	Klebsiella pneumoniae IS39	W1HVP3	**2**	MKKLRWVLLI VIIAGCLLLW TQMLNVMCDQ DVQFFSGICT INKFIPW	47
208	Klebsiella pneumoniae IS46	W1E5D7	**2**	MKKLRWVLLI VIIAGCLLLW TQMLNVMCDQ DVQFFSGICT INKFIPW	47

209	Klebsiella pneumoniae subsp. pneumoniae	A0A075QZ34	**2**	MKKLRWVLLI VIIAGCLLLW TQMLNVMCDQ DVQFFSGICT INKFIRW	47
210	Klebsiella pneumoniae subsp. pneumoniae	A0A075QQ95	**2**	MKKLRWVLLI VIIAGCLLLW TQMHNVMCDQ DVQFFSGICT INKFIPW	47
211	Klebsiella pneumoniae subsp. pneumoniae	A0A075QUV4	**2**	MKKLRWVLLI VIIAGCLLLW TQMLNVMCDQ DVQFFSSICT INKFIPW	47
212	Klebsiella pneumoniae subsp. pneumoniae	A0A1Y0Q1N0	**2**	MKKLRWVLLI VIIAGCLLLW TQMLNVMCDQ DVQFFSGICT INKFIPW	47
213	Klebsiella pneumoniae subsp. rhinoscleromatis ATCC 13884	C8T3F0	**2**	MKKLRWVLLI VIIAGCLLLW TQMLNVMCDQ DVQFFSGICT INKFIPW	47
214	Klebsiella quasipneumoniae	A0A1C1F5A7	**2**	MKKLRWVLLI VIIAGCLLLW TQMLNVMCDQ DVQFFSGICT INKFIPW	47
215	Klebsiella quasivariicola	A0A223U9L9	**?**	MKKLRWVLLI VIIAGCLLLW TQMLNVMCDQ DVQFFSGICT INKFIPW	47
216	Klebsiella sp. HMSC16C06	A0A1F2LXV1	**?**	MKKLRWVLLI VIIAGCLLLW TQMLNVMCDQ DVQFFSGICT INKFIPW	47
217	Klebsiella sp. LTGPAF-6F	A0A1D8JVR0	**2**	MKKLRWVLLI VIIAGCLLLW TQMLNVMCDQ DVQFFSGICT INKFIPW	47
218	Klebsiella sp. OBRC7	J6IIL4	**2**	MKKLRWVLLI VIIAGCLLLW TQMLNVMCDQ DVQFFSGICT INKFIPW	47
219	Kluyvera ascorbata ATCC 33433	A0A085IKE1	**2**	MKKFRWVLLI LVIAACLLLW TQMINVMCDQ DVQFFSGICS INRFIPW	47
220	Kluyvera georgiana	A0A248KI92	**1**	MKKFRWVLLI IVIAACLLLW TQMINVMCDQ DVQFFSGVCS INRFIPW	47
221	Kluyvera georgiana ATCC 51603	A0A1B7K139	**1**	MKKFRWVLLI LVIAACLLLW TQMINVMCDQ DVQFFSGVCS INRFIPW	47
222	Kluyvera intermedia (Enterobacter intermedius)	A0A1X0XCU6	**2**	MKKFRWLILI AVLVVCLLLW TQMLNVMCDQ DVQFFSGICV INKYIPW	47
223	Kosakonia arachidis	A0A1I7D940	**1**	MRKYKWLILI VVLVGCLLLW TQMLNVMCDQ DVQFFSGICI VNKFIPW	47
224	Kosakonia cowanii	A0A1U7E6G5	**2**	MRKFRWLLLI AVVVVCLLLW TQMINVMCDQ DVQFFSGICV INKFIPW	47
225	Kosakonia oryzae	A0A1T5HKY3	**?**	MRKYKWLILI VVLVGCLLLW TQMLNVMCDQ DVQFFSGICI VNKFIPW	47
226	Kosakonia radicincitans DSM 16656	A0A1V0LCU2	**1**	MRKYKWLILI VVLVGCLLLW TQMLNVMCDQ DVQFFSGICI VNKFIPW	47
227	Leclercia adecarboxylata	A0A1V2Z264	**2**	MKKIRWVILV LVLLACVLLW TQTINVMCDQ DVQFFSGICS VNKFIPW	47
228	Leclercia sp. LK8	A0A0J6MKQ4	**2**	MKKIRWVILI IVLIACVVLW TQTINVMCDQ DVQFFSGVCA INKFIPW	47
229	Lelliottia amnigena (Enterobacter amnigenus)	A0A1A9FDE2	**2**	MKKIRWVILI IVLVACAFLW TQTINVMCDQ DVQFFSGICA INKFIPW	47
230	Lelliottia amnigena CHS 78	A0A066NIN8	**2**	MKKIRWVILI IVLIACVVLW TQTINVMCDQ DVQFFSGICS INKFIPW	47

231	Lelliottia nimipressuralis	A0A1J5VZJ2	**1**	MKKIRWVILI IVLIACVVLW TQTINVMCDQ DVQFFSGVCA INKFIPW	47
232	Pantoea sesami	A0A1M5IUR2	**?**	MKKIRWVILI IVLIACVVLW TQTINVMCDQ DVQFFSGVCA INKFIPW	47
233	Proteus sp. HMSC10D02	A0A1F2HEE0	**2**	MKKLRWVLLI VIIAGCLLLW TQMLNVMCDQ DVQFFSGICT INKFIPW	47
234	Raoultella ornithinolytica (Klebsiella ornithinolytica)	A0A0B5IIP7	**2**	MKKLRWVLLI VVIAGCLLLW TQMLNVMCDQ DVQFFSGICT INKFIPW	47
235	Raoultella ornithinolytica (Klebsiella ornithinolytica)	A0A225UAF4	**2**	MKKVRWVLLI VIIAGCLLLW TQMLNVMCDQ DVQFFSGICT INKFIPW	47
236	Raoultella terrigena (Klebsiella terrigena)	A0A1V2BHW5	**1**	MKKLRWVLLI VVIAGCLLLW TQMLNVMCDQ DVQFFSGICT INKFIPW	47
237	Salmonella enterica subsp. enterica serovar agona (strain SL483)	B5F3Q2	**2**	MKKFRWVVLG IVVVVCLLLW AQVFNIMCDQ DVQFFSGICA INKFIPW	47
238	Salmonella enterica subsp. arizonae (strain ATCC BAA-731 / CDC346-86 / RSK2980)	A9MNI4	**2**	MKKFRWVVLG IVVVVCLLLW AQVFNIMCDQ DVQFFSGICA INKFIPW	47
239	Salmonella bongori N268-08	S5N950	**2**	MKKFRWVVLG IVVVVCLLLW AQVFNIMCDQ DVQFFSGICA INKFIPW	47
240	Salmonella bongori serovar 66:z41:- str. SA19983605	A0A248K855	**2**	MKKFRWVVLG IVVVVCLLLW AQVFNIMCDQ DVQFFSGICA INKFIPW	47
241	Salmonella enterica subsp. enterica serovar choleraesuis	A0A0M0PHZ0	**2**	MKKFRWVVLG IVVVVCLLLW AQVFNIMCDQ DVQFFSGICA INKFIPW	47
242	Salmonella choleraesuis	A0A117HVJ7	**2**	MKKIRWVVLI VVVLVCILMW AQVFNIMCDQ DVQFFSGICA INKFIPW	47
243	Salmonella choleraesuis (strain SC-B67)	Q57NG9	**2**	MKKFRWVVLG IVVVVCLLLW AQVFNIMCDQ DVQFFSGICA INKFIPW	47
244	Salmonella enterica subsp. enterica serovar dublin (strain CT_02021853)	B5FTJ3	**2**	MKTFRWVVLG IVVVVCLLLW AQVFNIMCDQ DVQFFSGICA INKFIPW	47
245	Salmonella enterica I	A0A0F7J939	**2**	MKKFRWVVLG IVVVVCLLLW AQVFNIMCDQ DVQFFSGICA INKFIPW	47
246	Salmonella enterica subsp. arizonae serovar 18:z4,z23:- str. CVM N6509	A0A1M3XZH0	**2**	MKKFRWVVLG IVVVVCLLLW AQVFNIMCDQ DVQFFSGICA INKFIPW	47
247	Salmonella enterica subsp. arizonae serovar 41:z4,z23:-	A0A1X2TN92	**2**	MKKFRWVVLG IVVVVCLLLW AQVFNIMCDQ DVQFFSGICA INKFIPW	47
248	Salmonella enterica subsp. arizonae serovar 50:r:z	A0A1X2TGL5	**2**	MKKFRWVVLG IVVVVCLLLW AQVFNIMCDQ DVQFFSGICA INKFIPW	47

249	Salmonella enterica subsp. arizonae serovar 62:z36:- str. RKS2983	A0A089HJH6	**2**	MKKFRWVVLG IVVVVCLLLW AQVFNIMCDQ DVQFFSGICA INKFIPW	47
250	Salmonella enterica subsp. arizonae serovar 63:g,z51:- str. So 20/20	A0A0V2EBP3	**2**	MKKFRWVVLG IVVVVCLLLW AQVFNIMCDQ DVQFFSGICA INKFIPW	47
251	Salmonella enterica subsp. diarizonae serovar 50:k:z str. MZ0080	A0A241RCQ3	**2**	MKKFRWVVLG IVVVVCLLLW AQVFNIMCDQ DVQFFSGICA INKFIPW	47
252	Salmonella enterica subsp. diarizonae serovar 60:r:e,n,x,z15	A0A232RKS9	**2**	MKKFRWVVLG IVVVVCLLLW AQVFNIMCDQ DVQFFSGICA INKFIPW	47
253	Salmonella enterica subsp. diarizonae serovar 65:c:z str. SA20044251	A0A241RZB4	**2**	MKKFRWVVLG IVVVVCLLLW AQVFNIMCDQ DVQFFSGICA INKFIPW	47
254	Salmonella enterica subsp. enterica serovar Adelaide str. A4-669	G5L875	**2**	MKKFRWVVLG IVVVVCLLLW AQVFNIMCDQ DVQFFSGICA INKFIPW	47
255	Salmonella enterica subsp. enterica serovar Alachua str. R6-377	G5LMX1	**2**	MKKFRWVVLG IVVVVCLLLW AQVFNIMCDQ DVQFFSGICA INKFIPW	47
256	Salmonella enterica subsp. enterica serovar Bareilly	A0A1S1AH73	**2**	MKKFRWVVLG IVVVVCLLLW AQVFNIMCDQ DVQFFSGICA INKFIPW	47
257	Salmonella enterica subsp. enterica serovar Bovismorbificans	A0A0U0WUY1	**2**	MKKFRWVVLG IVVVVCLLLW AQVFNIMCDQ DVQFFSGICA INKFIPW	47
258	Salmonella enterica subsp. enterica serovar Cerro str. CFSAN001590	V7UAQ6	**2**	MKKFRWVVLG IVVVVCLLLW AQVFNIMCDQ DVQFFSGICA INKFIPW	47
259	Salmonella enterica subsp. enterica serovar Cubana str. 76814	V7IPT1	**2**	MKKFRWVVLG IVVVVCLLLW AQVFNIMCDQ DVQFFSGICA INKFIPW	47
260	Salmonella enterica subsp. enterica serovar Cubana str. CFSAN002050	S5HS98	**2**	MKKFRWVVLG IVVVVCLLLW AQVFNIMCDQ DVQFFSGICA INKFIPW	47
261	Salmonella enterica subsp. enterica serovar Dublin str. UC16	M7S840	**2**	MKTFRWVVLG IVVVVCLLLW AQVFNIMCDQ DVQFFSGICA INKFIPW	47
262	Salmonella enterica subsp. enterica serovar Gallinarum str. SG9	A0A0G2NLX0	**2**	MKKFRWVVLG IVVVVCLLLW AQVFNIMCNQ DVQFFSGICA INKFIPW	47

263	Salmonella enterica subsp. enterica serovar Give str. S5-487	G5MHC7	**2**	MKKFRWVVLG IVVVVCLLLW AQVFNIMCDQ DVQFFSGICA INKFIPW	47
264	Salmonella enterica subsp. enterica serovar Hvittingfoss str. SA20014981	A0A221YZB0	**2**	MKKFRWVVLG IVVVVCLLLW AQVFNIMCDQ DVQFFSGICA INKFIPW	47
265	Salmonella enterica subsp. enterica serovar India str. SA20085604	A0A1Z3QDI2	**2**	MKKFRWVVLG IVVVVCLLLW AQVFNIMCDQ DVQFFSGICA INKFIPW	47
266	Salmonella enterica subsp. enterica serovar Infantis str. SARB27	G4C2K8	**2**	MKKFRWVVLG IVVVVCLLLW AQVFNIMCDQ DVQFFSGICA INKFIPW	47
267	Salmonella enterica subsp. enterica serovar Inverness str. R8-3668	G5NBY2	**2**	MKKFRWVVLG IVVVVCLLLW AQVFNIMCDQ DVQFFSGICA INKFIPW	47
268	Salmonella enterica subsp. enterica serovar Johannesburg	A0A0L9F773	**2**	MKKFRWVVLG IVVVVCLLLW AQVFNIMCDQ DVQFFSGICA INKFIPW	47
269	Salmonella enterica subsp. enterica serovar Kentucky str. SA20030505	A0A221YLJ6	**2**	MKKFRWVVLG IVVVVCLLLW AQVFNIMCDQ DVQFFSGICA INKFIPW	47
270	Salmonella enterica subsp. enterica serovar Mississippi str. A4-633	G5PM42	**2**	MKKFRWVVLG IVVVVCLLLW AQVFNIMCDQ DVQFFSGICA INKFIPW	47
271	Salmonella enterica subsp. enterica serovar Montevideo str. 609458-2	A0A1C3A297	**2**	MKKFRWVVLG IVVVVCLLLW AQVFNIMCDQ DVQFFSGICA INKFIPW	47
272	Salmonella enterica subsp. enterica serovar Montevideo str. S5-403	G5Q1K9	**2**	MKKFRWVVLG IVVVVCLLLW AQVFNIMCDQ DVQFFSGICA INKFIPW	47
273	Salmonella enterica subsp. enterica serovar Muenchen str. baa1594	V1VZ47	**2**	MKKFRWVVLG IVVVVCLLLW AQVFNIMCDQ DVQFFSGICA INKFIPW	47
274	Salmonella enterica subsp. enterica serovar Rough O:d:1,7	A0A1X2RFL4	**2**	MKKFRWVVLG IVVVVCLLLW AQVFNIMCDQ DVQFFSGICA INKFIPW	47
275	Salmonella enterica subsp. enterica serovar Rubislaw str. A4-653	G5QI15	**2**	MKKFRWVVLG IVVVVCLLLW AQVFNIMCDQ DVQFFSGICA INKFIPW	47
276	Salmonella enterica subsp. enterica serovar Saintpaul	A0A1S0ZF23	**2**	MKKFRWVVLG IVVVVCLLLW AQVFNIMCDQ DVQFFSGICA INKFIPW	47

277	Salmonella enterica subsp. enterica serovar Saphra	A0A1X2YCS1	**2**	MKKFRWVVLG IVVVVCLLLW AQVFNIMCDQ DVQFFSGICA INKFIPW	47
278	Salmonella enterica subsp. enterica serovar Seftenburg	A0A1X2UNT4	**2**	MKKFRWVVLG IVVVVCLLLW AQVFNIMCDQ DVQFFSGICA INKFIPW	47
279	Salmonella enterica subsp. enterica serovar Senftenberg str. A4-543	G5QYU8	**2**	MKKFRWVVLG IVVVVCLLLW AQVFNIMCDQ DVQFFSGICA INKFIPW	47
280	Salmonella enterica subsp. enterica serovar Tennessee str. TXSC_TXSC08-19	X2KJG1	**2**	MKKFRWVVLG IVVVVCLLLW AQVFNIMCDQ DVQFFSGICA INKFIPW	47
281	Salmonella enterica subsp. enterica serovar Typhimurium str. DT104	A0A0U1G4T3	**2**	MKKFRWVVLG IVVVVCLLLW AQVFNIMCDQ DVQFFSGICA INKFIPW	47
282	Salmonella enterica subsp. enterica serovar Uganda str. R8-3404	G5REM6	**2**	MKKFRWVVLG IVVVVCLLLW AQVFNIMCDQ DVQFFSGICA INKFIPW	47
283	Salmonella enterica subsp. enterica serovar Urbana str. R8-2977	G5RUD1	**2**	MKKFRWVVLG IVVVVCLLLW AQVFNIMCDQ DVQFFSGICA INKFIPW	47
284	Salmonella enterica subsp. enterica serovar Wandsworth str. A4-580	G5SAS6	**2**	MKKFRWVVLG IVVVVCLLLW AQVFNIMCDQ DVQFFSGICA INKFIPW	47
285	Salmonella enterica subsp. indica serovar 6,14,25:z10:1,(2),7 str. 1121	V1GR58	**2**	MKKFRWVVLG IVVVVCLLLW AQVFNIMCDQ DVQFFSGICA INKFIPW	47
286	Salmonella enterica subsp. salamae	A0A0F5BER6	**2**	MKKFRWVVLG IVVVVCLLLW AQVFNIMCDQ DVQFFSGICA INKFIPW	47
287	Salmonella enterica subsp. salamae serovar 55:k:z39 str. 1315K	A0A241SEH7	**2**	MKKFRWVVLG IVVVVCLLLW AQVFNIMCDQ DVQFFSGICA INKFIPW	47
288	Salmonella enterica subsp. salamae serovar 56:z10:e,n,x str. 1369-73	A0A0V2CSS7	**2**	MKKFRWVVLG IVVVVCLLLW AQVFNIMCDQ DVQFFSGICA INKFIPW	47
289	Salmonella enteritidis	A0A1R2TMC4	**2**	MKKFRWVVLG IVVVVCLLLW AQVFNIMCDQ DVQFFSGICA INKFIPW	47
290	Salmonella enterica subsp. enterica serovar enteritidis PT4 (strain P125109)	B5R2T1	**2**	MKKFRWVVLG IVVVVCLLLW AQVFNIMCDQ DVQFFSGICA INKFIPW	47

291	Salmonella enterica subsp. enterica serovar gallinarum (strain 287/91 / NCTC 13346)	B5R8W2	**2**	MKKFRWVVLG IVVVVCLLLW AQVFNIMCNQ DVQFFSGICA INKFIPW	47
292	Salmonella enterica subsp. enterica serovar Heidelberg (strain SL476)	B4TKH1	**2**	MKKFRWVVLG IVVVVCLLLW AQVFNIMCDQ DVQFFSGICA INKFIPW	47
293	Salmonella enterica subsp. houtenae	A0A1J7KM92	**2**	MKKFRWVVLG IVVVVCLLLW AQVFNIMCDQ DVQFFSGICA INKFIPW	47
294	Salmonella enterica subsp. enterica serovar newport	A0A0R9ND34	**?**	MKKFRWVVLG IVVVVCLLLW AQVFNIMCDQ DVQFFSGICA INKFIPW	47
295	Salmonella newport (strain SL254)	B4SV80	**?**	MKKFRWVVLG IVVVVCLLLW AQVFNIMCDQ DVQFFSGICA INKFIPW	47
296	Salmonella enterica subsp. enterica serovar paratyphi A (strain AKU_12601)	B5BHB6	**2**	MKKFRWVVLG IVVVVCLLLW AQVFNIMCDQ DVQFFSGICA INKFIPW	47
297	Salmonella paratyphi A (strain ATCC 9150 / SARB42)	Q5PNK5	**2**	MKKFRWVVLG IVVVVCLLLW AQVFNIMCDQ DVQFFSGICA INKFIPW	47
298	Salmonella paratyphi B (strain ATCC BAA-1250 / SPB7)	A9MV69	**2**	MKKFRWVVLG IVVVVCLLLW AQVFNIMCDQ DVQFFSGICA INKFIPW	47
299	Salmonella paratyphi C (strain RKS4594)	C0Q2Z5	**2**	MKKFRWVVLG IVVVVCLLLW AQVFNIMCDQ DVQFFSGICA INKFIPW	47
300	Salmonella enterica subsp. enterica serovar schwarzengrund (strain CVM19633)	B4TY09	**?**	MKKFRWVVLG IVVVVCLLLW AQVFNIMCDQ DVQFFSGICA INKFIPW	47
301	Salmonella sp. HMSC13B08	A0A1F2JBN1	**?**	MKKFRWVILI IVALVCLLLW AQVFNIMCDQ DVQFFNGICA INKFIPW	47
302	Salmonella enterica subsp. enterica serovar typhi	Q8XG71	**3**	MKKFRWVVLG IVVVVCLLLW AQVFNIMCDQ DVQFFSGICA INKFIPW	47
303	Salmonella enterica subsp. enterica serovar typhimurium	A0A0J0VVZ8	**2**	MKKFRWVVLG IVVVVCLLLW AQVFNIMCDQ DVQFFSGICA INKFIPW	47
304	Salmonella typhimurium (strain 14028s / SGSC 2262)	D0ZK39	**2**	MKKFRWVVLG IVVVVCLLLW AQVFNIMCDQ DVQFFSGICA INKFIPW	47
305	Salmonella typhimurium (strain 4/74)	E8X999	**2**	MKKFRWVVLG IVVVVCLLLW AQVFNIMCDQ DVQFFSGICA INKFIPW	47
306	Salmonella typhimurium (strain LT2 / SGSC1412 / ATCC 700720)	Q7CQD5	**2**	MKKFRWVVLG IVVVVCLLLW AQVFNIMCDQ DVQFFSGICA INKFIPW	47
307	Salmonella typhimurium (strain SL1344)	A0A0H3NDR4	**2**	MKKFRWVVLG IVVVVCLLLW AQVFNIMCDQ DVQFFSGICA INKFIPW	47

308	Shigella boydii	A0A1Q8MEG0	**2**	MKKFRWVVLV VVVLACLLLW AQVFNMMCDQ DVQFFSGICA INQFIPW	47
309	Shigella boydii 4444-74	I6E5W5	**2**	MKKFRWVVLV VVVLACLLLW AQVFNMMCDQ DVQFFSGICA INQFIPW	47
310	Shigella boydii 5216-82	F3WJ64	**2**	MKKFRWVVLV VVVLACLLLW AQVFNMMCDQ DVQFFSGICA INQFIPW	47
311	Shigella boydii 965-58	I6DGX5	**2**	MKKFRWVVLV VVVLACLLLW AQVFNMMCDQ DVQFFSGICA INQFIPW	47
312	Shigella boydii ATCC 9905	E7SVL3	**2**	MKKFRWVVLV VVVLACLLLW AQVFNMMCDQ DVQFFSGICA INQFIPW	47
313	Shigella boydii serotype 18 (strain CDC 3083-94 / BS512)	B2U467	**2**	MKKFRWVVLV VVVLACLLLW AQVFNMMCDQ DVQFFSGICA INQFIPW	47
314	Shigella boydii serotype 4 (strain Sb227)	Q321Z1	**2**	MKKFRWVVLV VVVLACLLLW AQVFNMMCDQ DVQFFSGICA INQFIPW	47
315	Shigella dysenteriae	A0A1Q8NRP2	**3**	MKKFRWVVLV VVVLACLLLW AQVFNMMCDQ DVQFFSGICA INQFIPW	47
316	Shigella dysenteriae 1012	B3X699	**3**	MKKFRWVVLV VVVLACLLLW AQVFNMMCDQ DVQFFSGICA INQFIPW	47
317	Shigella dysenteriae 1617	E2XFQ9	**3**	MKKFRWVVLV VVVLACLLLW AQVFNMMCDQ DVQFFSGICA INQFIPW	47
318	Shigella dysenteriae 225-75	I6FV90	**3**	MKKFRWVVLV VVVLACLLLW AQVFNMMCDQ DVQFFSGICA INQFIPW	47
319	Shigella dysenteriae CDC 74-1112	E7SEJ5	**3**	MKKFRWVVLV VVVLACLLLW AQVFNMMCDQ DVQFFSGICA INQFIPW	47
320	Shigella dysenteriae serotype 1 (strain Sd197)	Q32F32	**3**	MKKFRWVVLV VVVLACLLLW AQVFNMMCDQ DVQFFSGICA INQFIPW	47
321	Shigella dysenteriae WRSd3	A0A090NX03	**2**	MKKFRWVVLV VVVLACLLLW AQVFNMMCDQ DVQFFSGICA INQFIPW	47
322	Shigella flexneri	P64514	**2**	MKKFRWVVLV VVVLACLLLW AQVFNMMCDQ DVQFFSGICA INQFIPW	47
323	Shigella flexneri	A0A1W2MJ79	**2**	MKKFRWVVLV VVVLACLLLW AQVFNMMCDQ DVQFFSGICA INQFIPW	47
324	Shigella flexneri 1235-66	I6H4S5	**2**	MKKFRWVVLV VVVLACLLLW AQVFNMMCDQ DVQFFSGICA INQFIPW	47
325	Shigella flexneri 1485-80	K0X9Q9	**2**	MKKFRWVVLV VVVLACLLLW AQVFNMMCDQ DVQFFSGICA INQFIPW	47
326	Shigella flexneri 2850-71	I6BSU2	**2**	MKKFRWVVLV VVVLACLLLW AQVFNMMCDQ DVQFFSGICA INQFIPW	47
327	Shigella flexneri 2a str. 301	A0A226K966	**2**	MKKFRWVVLV VVVLACLLLW AQVFNMMCDQ DVQFFSGICA INQFIPW	47
328	Shigella flexneri 4c	A0A127GKV5	**2**	MKKFRWVVLV VVVLACLLLW AQVFNMMCDQ DVQFFSGICA INQFIPW	47
329	Shigella flexneri 5a str. M90T	A0A0F6MCX8	**2**	MKKFRWVVLV VVVLACLLLW AQVFNMMCDQ DVQFFSGICA INQFIPW	47
330	Shigella flexneri K-227	F5NUD0	**2**	MKKFRWVVLV VVVLACLLLW AQVFNMMCDQ DVQFFSGICA INQFIPW	47
331	Shigella flexneri K-315	I6CV84	**2**	MKKFRWVVLV VVVLACLLLW AQVFNMMCDQ DVQFFSGICA INQFIPW	47

332	Shigella flexneri serotype 5b (strain 8401)	Q0T519	**2**	MKKFRWVVLV VVVLACLLLW AQVFNMMCDQ DVQFFSGICA INQFIPW	47
333	Shigella flexneri serotype X (strain 2002017)	D2AEJ6	**2**	MKKFRWVVLV VVVLACLLLW AQVFNMMCDQ DVQFFSGICA INQFIPW	47
334	Shigella flexneri VA-6	F5N2I2	**2**	MKKFRWVVLV VVVLACLLLW AQVFNMMCDQ DVQFFSGICA INQFIPW	47
335	Shigella sonnei	A0A1S9JSK5	**2**	MKKFRWVVLV VVVLACLLLW AQVFNMMCDQ DVQFFSGICA INQFIPW	47
336	Shigella sonnei (strain Ss046)	Q3Z2G6	**2**	MKKFRWVVLV VVVLACLLLW AQVFNMMCDQ DVQFFSGICA INQFIPW	47
337	Shigella sp. FC2928	A0A1E2VFN8	**?**	MKKFRWVVLV VVVLACLLLW AQVFNMMCDQ DVQFFSGICA INQFIPW	47
338	Shigella sp. FC569	A0A1E3N701	**?**	MKKFRWVALV VVVLACLLLW AQVFNMMCDQ DVQFFSGICA INQFIPW	47
339	Shigella sp. PAMC 28760	A0A142H3S0	**?**	MKKFRWVVLV VVVLACLLLW AQVFNMMCDQ DVQFFSGICA INQFIPW	47
340	Trabulsiella guamensis ATCC 49490	A0A085ARL1	**1**	MKKYRWVILA VILLLCVLLW TQTINVMCDQ DVQFFSGICT INKFIPW	47
341	Trabulsiella odontotermitis	A0A0L0GYS9	**1**	MKKYRWVILT VILLLCVLLW TQTINVMCDQ DVQFFSGICT INKFIPW	47
342	uncultured Citrobacter sp.	A0A212IN55	**2**	MKKIRWVVLI VVVLVCILMW AQVFNIMCDQ DVQFFSGICA INKFIPW	47
343	Yersinia enterocolitica	A0A1W9F9C3	**2**	MNIKKLVATV GIIAVCCLFY LLALDSYCDQ GGTFSTGICA ITSIIPW	47
344	Yersinia enterocolitica subsp. palearctica serotype O:3 (strain DSM 13030 / CIP 106945 / Y11)	A0A0H3NZR3	**2**	MNIKKLVATV GIIAVCCLFY LLALDSYCDQ GGTFSTGMCT ITSIIPW	47
345	Yersinia enterocolitica W22703	F4MZ75	**1**	MLNKVPDLNI KKLVATVGII AVCCLFYLLA LDSYCDQGGT FSTGICTITS IIPW	47
346	Yersinia entomophaga	A0A210TKN0	**1**	MNIKKLVATV ILIAVCCLFY LLALDSYCDQ GGNFSNGICS ITAIVPW	47
347	Yersinia frederiksenii	A0A209AFG9	**2**	MNIKKLVATV GIIAVCCLFY LLALDSYCDQ GGTFSTGICT ITSIIPW	47
348	Yersinia intermedia	A0A209A4K4	**2**	MNIKKLVTAV GIIAVCCLFY LLALDSYCDQ GGTFSTGICA ITSIIPW	47
349	Yersinia kristensenii	A0A209A3E7	**2**	MNIKKLVAAV VIIAVCCLFY LLALDSYCDQ GGTFSMGICT ITSIIPW	47
350	Yersinia pestis subsp. microtus	A0A208S5N8	**3**	MNITKLVTTV VIIAACCLFY LLALDSYCDQ GGTFSTGICA ITTIVPW	47
