# Supplementary material for: Bacterial MgrB peptide activates chemoreceptor Fpr3 in mouse accessory olfactory system and drives avoidance behaviour
Source: Nat Commun. 2019 Oct 25;10:4889. doi: 10.1038/s41467-019-12842-x (PMC6814738; doi:10.1038/s41467-019-12842-x)
Supplement: Supplementary file 6 — Supplementary Data 4 [file 41467_2019_12842_MOESM6_ESM.pdf]

Supplementary Data 4 | Clinical relevance of the most frequent MgrB-encoding bacterial species.

Bacteria with *mgrB* gene	Complete MgrB sequence	Pathogenicity of the most frequent MgrB containing bacterial species
Salmonella sp. **(71)**<br>*S. enterica* (43)<br>*S.* Typhimurium (5)<br>*S.* Paratyphi (4)	**MKKFRWVVL**GIVVVVCLLLWAQVFNI MCDQDVQFFSGICAINKFIPW	The genus *Salmonella* contains two species, *S. bongori* and *S. enterica*, with the latter species being subdivided into seven subspecies and approximately 2500 serovars. A number of *S. enterica* serovars such as Enteritidis, Typhi, Typhimurium, and Paratyphi are obligate pathogens capable of causing diseases such as gastroenteritis, sepsis, and typhoid fever. While some serovars such as Typhi are only infectious for humans, others have the potential to infect humans and other mammals, including mice.
Shigella sp. **(32)**<br>*S. flexneri* (13)<br>*S. boydii* (7)<br>*S. dysenteriae* (7)<br>*S. sonnei* (2)	**MKKFRWVVL**VVVVLACLLLWAQVFN MMCDQDVQFFSGICAINQFIPW	The genus *Shigella* encompasses four species, which are all pathogenic to humans. They are a major source of foodborne illness and usually require few bacteria to initiate disease. The main clinical symptoms caused by these species are diarrhea, fever, and abdominal pain. A very severe form of dysentery (diarrhea with blood) is caused by *S. dysenteriae* (a risk group 3 organism) due to the production of shiga toxin, a potent inhibitor of protein synthesis.
Escherichia sp. **(132)**<br>*E. coli* (128)<br>EHEC (6)<br>DEC (6)<br>*E. albertii* (2)<br>*E. fergusonii* (2)	**MKKFRWVVL**VVVVLACLLLWAQVFN MMCDQDVQFFSGICAINQFIPW	The genus *Escherichia* with its type species *E. coli* covers a large and diverse group of bacteria. Most *E. coli* strains are harmless commensals and major components of the gut microflora in mammals, but may induce illness outside of the intestinal tract. A small number of highly adapted *E. coli* clones, often referred to as diarrheagenic *E. coli*, have developed the ability to cause severe diseases even in healthy humans. These pathogenic *E. coli* variants can be divided into at least six different categories with corresponding distinct pathogenic schemes. Of particular clinical relevance are enterohemorrhagic *E. coli* (EHEC), which are classified as risk group 3 organisms, and require a very small infectious dose (as little as 10 bacterial cells) to cause disease in humans.
Enterobacter sp. **(42)**<br>*E. cloacae* (22)<br>*E. kobei* (4)<br>*E. hormaechei* (3)	**MKKIRWVIL**VIVLIACVVLWTQTINVM CDQDVQFFSGVCAINKFIPW	The genus *Enterobacter* comprises 58 species, some of which may cause opportunistic infections in immunocompromised hosts. The clinical most important species from this genus is *E. cloacae*, which is a member of the gut microbiome of healthy humans, but also a common cause for urinary and respiratory tract infections in hospitalized patients.
Klebsiella sp. **(29)**<br>*K. pneumoniae* (17)<br>*K. oxytoca* (3)<br>*K. aerogenes* (3)	**MKKLRWVLL**IVIIAGCLLLWTQMLNV MCDQDVQFFSGICTINKFIPW	The genus *Klebsiella* encompasses a group of about 17 species. Most of them are commensal bacteria found in the gut microbiomes of mammals, but some may cause disease in humans and animals. Clinically relevant species are *K. aerogenes* (associated with bacteremia in hospitalized patients), *K. oxytoca* (associated with respiratory diseases, gastro-intestinal infection, and sepsis), and *K. pneumoniae* (a major cause of nosocomial pneumonia and urinary tract infections). The latter species is also ubiquitous in nature, and found in vegetation, soil, and surface waters.
